# Supplementary material for: Understanding responsibility for health inequalities in children’s hospitals in England: a qualitative study with hospital staff
Source: BMJ Open. 2024 Apr 10;14(4):e081056. doi: 10.1136/bmjopen-2023-081056 (PMC11015292; doi:10.1136/bmjopen-2023-081056)
Supplement: Supplementary data [file bmjopen-2023-081056supp001.pdf]

FULL/LONG TITLE OF THE STUDY

Children's Hospitals Inequalities Research Project

SHORT STUDY TITLE / ACRONYM

CHIRP

PROTOCOL VERSION NUMBER AND DATE

V1.0 21 April 2022

RESEARCH REFERENCE NUMBERS

**IRAS Number:**	315113
**FUNDERS Number:**	SCH5628 Sheffield Children's Hospitals NHS Foundation Trust (SCH)<br>National Paediatric Accelerator

STUDY PROTOCOL

Children's Hospitals Inequalities Research Project

1 BACKGROUND

This research will investigate how the ten children's hospitals in the Children's Hospital Alliance (England) view their responsibility to reduce socio-economic health inequalities (e.g. income, location, employment, education, cultural and religious barriers) and how these translate into actions. As part of the National Paediatric Accelerator Programme, we have been asked to look at organisational policies (collect documents) and talk to different groups of staff (in interviews and focus groups) about inequalities experienced by patients, how these affect care (e.g. by being able to attend appointments) and to identify recommendations to develop good practice to ultimately reduce the number of 'Was not Broughts' (the children's equivalent of "Did not attend"), and improve care.

This study will collect qualitative data via interviews and focus groups, which will take place online or at the participants' workplaces. NHS staff participants will be recruited via their organisations. They will include members of the senior leadership team (e.g. executive/ non-executive director plus any other relevant senior leader depending on the structure of the organisation), doctors with an interest in health inequalities, nursing and allied health professional staff (e.g. physiotherapists, occupational therapists), and professional, administrative and support staff (e.g. hospital teachers, play leaders, secretaries, receptionists, porters, security, cleaners).

2 RATIONALE

The ten children's hospitals involved in the study have been collaborating through the National Paediatric Accelerator Programme to support elective recovery, share best practice and reduce health inequalities. As a group, the organisations are seeking to develop understanding as to how acute trusts working together, can improve access to quality paediatric healthcare. The work has included looking at the inequitable impact that the SARS-CoV-2/COVID pandemic has had on the health and wellbeing of children from the poorest backgrounds. In particular, focus has been on the disproportionate impact that deprivation has had on the ability of children from different socioeconomic backgrounds to access healthcare. Children from the most deprived decile in the index of multiple deprivation are more than twice as likely to not be brought to their outpatients' appointments (Edge Health, 2021) than those from the least deprived decile.

It is relatively new for NHS trusts to think about, and seek to take action on, health inequalities relating to deprivation. In considering its role in inequalities, the NHS is most familiar with its statutory role with regards to some specific equality domains. The Equality Act 2010 defines several protected characteristics (i.e. age, sex, race, disability, sexual orientation, gender reassignment, marriage and civil partnerships, pregnancy and maternity, religion or belief discrimination). By comparison with the protected characteristics, there is no specific duty on NHS organisations to intervene with regard to deprivation. However, the Health and Social Care Act 2012 introduced the first legal duties about health inequalities. Health inequalities are also part of several national strategies, such as the NHS Long Term Plan and the NHS People Plan. Initial analysis suggests that that socio-economic factors can have significant impacts on the ability of children and their parents, and recent analysis suggests that the situation has worsened during the pandemic (Marmot et al, 2020).

This research is part of the National Paediatric Accelerator Programme, which includes the remit to reduce the barriers to families preventing them from attending appointments with a view to ultimately reducing the number of 'Was not Broughts', which will support the elective recovery plan for Paediatrics. The research project will explore the roles that these ten trusts can play in reducing socio-economic barriers to paediatric care, and in understanding and developing best practice to do this.

Literature review

As part of the research project, we will conduct a literature-based analysis of the variation in governance approaches and policies around inequalities. This will include documents e.g. policies

Brewster L, *et al. BMJ Open* 2024; 14:e081056. doi: 10.1136/bmjopen-2023-081056

from the ten partner hospitals, published literature including how inequalities are experienced by children and young people in the UK, regional variation, impact of the SARS-CoV-2/COVID19 pandemic, grey literature around inequalities experienced by CYP, including identification of any global exemplars of how to reduce health inequalities.

3 RESEARCH QUESTION/AIM(S)

- How do the ten children's hospitals in the Children's Hospital Alliance (England) view their responsibility to reduce socio-economic health inequalities (e.g. income, location, employment, education, cultural and religious barriers)?

- How does this view of the potential responsibility to reduce socio-economic health inequalities translate into actions in the ten children's hospitals in the Children's Hospital Alliance (England)?

- Is there variation within governance approaches and policies across the ten children's hospitals in the Children's Hospital Alliance (England)?

- Are there areas where there are unmet needs for initiatives to reduce socio-economic health inequalities, either generally or due to changes in service delivery since the pandemic?

- How do the ten children's hospitals in the Children's Hospital Alliance (England) understand their role in relation to socio-economic inequality (e.g. inequalities in income, location, employment, education) where this impacts healthcare access and quality?

- How do the ten children's hospitals in the Children's Hospital Alliance (England) seek to mitigate the impacts of socio-economic inequality on healthcare?

- What are the barriers and enablers to initiatives that mitigate the impacts of socio-economic inequality on healthcare?

- What is perceived as best practice in attempts to mitigate the impacts of socio-economic inequality on healthcare?

Objectives

The objectives identified for the project are:

- Identify variation within governance approaches and policies across the ten children's hospitals in the Children's Hospital Alliance (England), summarising to help facilitate shared learning, by analysing organisational policies.
- Identify areas where there is unmet need for initiatives to reduce socio-economic health inequalities, either generally or due to changes in service delivery since the pandemic, by analysing organisational policies.
- Explore how the ten children's hospitals in the Children's Hospital Alliance (England) understand their role in relation to socio-economic inequality (e.g. inequalities in income, location, employment, education) where this impacts healthcare access and quality, through interviews and/or focus groups with various groups of staff.
- Consider how the ten children's hospitals in the Children's Hospital Alliance (England) are seeking to mitigate the impacts of socio-economic inequality on healthcare, including exploring what barriers and enablers exist, and what is perceived as best practice, through interviews and/or focus groups with various groups of staff.

Brewster L, et al. BMJ Open 2024; 14:e081056. doi: 10.1136/bmjopen-2023-081056

Outcome

The primary outcome will be a report to the funder, which contains practical recommendations about the roles that the ten children's hospitals in the Children's Hospital Alliance (England) can play in reducing socio-economic barriers to paediatric care, and in understanding and developing best practice to do this.

4 STUDY DESIGN and METHODS of DATA COLLECTION AND DATA ANALYIS

This project will collect qualitative data via interviews and focus groups, and conduct a scoping review of literature. We will work across the ten partner children's hospitals in the Children's Hospital Alliance (England). We aim to recruit participants as described from each site, as per the funder's aim, but are aware that this may not be possible within the available time. Our aim is to work across all ten locations, with a view to accessing some participants from each category in a minimum of six locations.

The qualitative approach has been selected in order to describe participants' understanding of current policy and practice around socio-economic inequalities and their impact on healthcare, rather than hypothesising about or predicting responses (Kvale & Brinkman, 2014). The qualitative study will gather information and key themes by using semi-structured interviews and focus groups to explore the participants' knowledge of how socio-economic inequalities are considered and managed within their organisation. Rapid Research Evaluation and Appraisal (RREAL) methodologies (Vindrola-Padros, 2021) will be used to analyse data; these methodologies emerge from a qualitative research tradition that recognises the need for rapid analysis of data to produce meaningful recommendations for practice in a timely manner.

Scoping Review: an analysis of the variation in governance approaches and policies around inequalities in children's hospitals to identify areas of best practice.

Academic and grey literature will be accessed by a rapid scoping review. Scoping reviews are used "to map rapidly the key concepts underpinning a research area and the main sources and types of evidence available" (Arksey & O'Malley, 2005) and can be particularly useful when the literature is disparate and has not been previously mapped (Daudt, van Mossel, & Scott, 2013). Recent work has clarified best practices for scoping reviews (Khalil et al., 2016; Peters et al., 2017). From this review, key works will be identified which will be used in analysis of data. This will include key policy documents from the trusts, alongside a grey literature search for national and international secondary healthcare providers to identify areas of best practice.

Qualitative approach: interviews

One interview of approximately 30-40 minutes will take place with each participant (members of the senior leadership team and doctors with an interest in reducing health inequalities) to address the research questions. Informed consent will be given at the start of the interview. We will aim to interview a minimum of six members of the senior leadership team and a minimum of six doctors. We will interview a maximum of thirty members of the senior leadership team and a maximum of thirty doctors. We expect a realistic sample size to be 10-15 members of the senior leadership team and 10-15 doctors.

The interviews will be conducted via telephone or online via Microsoft Teams. If preferred by participants and feasible for the research team, interviews may be conducted face to face. All participants taking part in the interviews must be able and willing to give informed consent.

A draft topic guide for the interviews has been created to guide discussion and maximise relevance and appropriateness. The topic guide will be used in the interviews to provide an initial structure and allow for comparability between respondents, while also allowing for flexibility and individuality in the responses given.

Brewster L, et al. BMJ Open 2024; 14:e081056. doi: 10.1136/bmjopen-2023-081056

Interviews will be recorded, and RAP (Rapid Appraisal) sheets will be completed electronically by the researcher undertaking the interview, and one other member of the research team.

Qualitative approach: focus groups

The aim will be to run two focus groups in each children's hospital, each lasting approx. 40-60 minutes. One focus group will be with 4-8 nurses and allied health professionals (e.g. physiotherapists, occupational therapists) and one with 4-8 members of professional, administrative and support staff (e.g. hospital teachers, play leaders, secretaries, receptionists, porters, security, cleaners). Again, our aim is to work across all ten locations, with a view to accessing some participants from each category in a minimum of six locations. This means that we will recruit a minimum of 24 and a maximum of 80 nurses and allied health professionals, and a minimum of 24 and a maximum of 80 professional, administrative and support staff to join focus groups and address the research questions.

The focus groups will be conducted face to face, unless prevented by pandemic infection prevention and control protocols. In this instance, focus groups will be conducted online via Microsoft Teams. All participants taking part in the focus groups must be able and willing to give informed consent.

A draft topic guide for the focus groups has been created to guide discussion and maximise relevance and appropriateness. Focus groups will be recorded, and RAP (Rapid Appraisal) sheets will be completed electronically by the researcher undertaking the interview, and one other member of the research team.

Data Analysis

Rapid assessment procedures aim to produce a rapid but rigorous qualitative analysis (Vindrola-Padros et al, 2020). It is predominantly team based and collaborative in nature, and conducted as an iterative process alongside data collection (Vindrola-Padros, 2021). RAP sheets will be used as the basis for debriefing sessions. Depending on team capacity, one or two RAP sheets will be completed for each interview or focus group (the aim will be two for each where possible). These standardised and structured data collection forms will guide our reflexive analysis process. In group debriefing sessions, we will focus on identifying key themes from these RAP sheets, creating brief summaries of data in tables that will then be used iteratively for cross-case comparison.

Using multiple population groups (senior leaders, doctors, nurses, allied health professionals and professional/ support staff), multiple data sources (published and grey literature, interviews and focus groups) and multiple experienced researchers means that we will be able to triangulate insights (Morse, 2015). Our aim is to produce a targeted analysis that leads to actionable recommendations and enables cross-case comparison between sites to identify good practices (Silverman, 2015).

5 STUDY SETTING

This is a multi-centre research project.

The ten children's hospitals in the Children's Hospital Alliance (England) have been collaborating through the National Paediatric Accelerator Programme to support elective recovery, share best-practice and reduce health inequalities. As a group, these organisations are seeking to develop understanding as to how acute trusts working together, can improve access to quality paediatric healthcare.

6 SAMPLE AND RECRUITMENT

Eligibility Criteria

All participants will have a current role in one of the ten children's hospitals in the Children's Hospital Alliance (England).

Inclusion criteria for the interviews:

1) A member of the senior leadership team (e.g. executive/ non-executive director plus any other relevant senior leader depending on the structure of the organisation) or a doctor with an interest in health inequalities.

2) Over 18 years.

3) Able and willing to give informed consent at the time of the interview.

Inclusion criteria for the focus groups:

1) Nursing and allied health professional staff (e.g. physiotherapists, occupational therapists), professional, administrative and support staff (e.g. hospital teachers, play leaders, secretaries, receptionists, porters, security, cleaners).

2) Over 18 years.

3) Able and willing to give informed consent at the time of the interview.

Exclusion criteria

1) Age <18 years.

2) Does not have a current role as listed above in one of the ten children's hospitals.

4) Unable or unwilling to give informed consent.

Sampling technique

We will work across ten children's hospitals. We aim to recruit participants as described from each site, as per the funder's aim, but are aware that this may not be possible within the available time. Our aim is to work across all ten locations, with a view to accessing some participants from each category in a minimum of six locations. In each organisation, we will sample:

1. 1-3 members of the senior leadership team (one executive and one non-executive director plus any other relevant senior leader depending on the structure of the organisation). All participants will be over 18 years old and recruited based on their professional role. Because of their professional roles, all participants will be English speaking. It is not proposed to exclude on any further criteria. We will recruit a minimum of six and a maximum of 30 people.

2. 1-3 doctors with an interest in reducing health inequalities. All participants will be over 18 years old and recruited based on their professional role. Because of their professional roles, all participants will be English speaking. It is not proposed to exclude on any further criteria. We will recruit a minimum of six and a maximum of 30 people.

Brewster L, *et al. BMJ Open* 2024; 14:e081056. doi: 10.1136/bmjopen-2023-081056

3. 4-8 nurses and allied health professionals (e.g. physiotherapists, occupational therapists). These potential participants will be recruited via their organisations. All participants will be over 18 years old and recruited based on their professional role. Because of their professional roles, all participants will be English speaking. It is not proposed to exclude on any further criteria. We will recruit a minimum of 24 and a maximum of 80 people.

4. 4-8 members of professional, administrative and support staff (e.g. hospital teachers, play leaders, secretaries, receptionists, porters, security, cleaners). These potential participants will be recruited via their organisations. All participants will be over 18 years old and recruited based on their professional role. Because of their professional roles, all participants will be English speaking. It is not proposed to exclude on any further criteria. We will recruit a minimum of 24 and a maximum of 80 people.

Recruitment

Senior leadership team: senior leaders will be identified via our gatekeeper in the National Paediatric Accelerator, who will provide organisational email addresses to the PI. Potential participants will then be provided with a detailed participant information sheet about the study and a copy of the consent form via email, and then asked to participate by Prof Isba as PI, on behalf of the research team. It will be made clear to potential participants that participation is voluntary.

Doctors: Doctors will be identified via our gatekeeper in the National Paediatric Accelerator who will consult each organisation to identify relevant individuals and provide organisational email addresses to the research team. Potential participants will then be provided with a detailed participant information sheet about the study and a copy of the consent form via email, and then asked to participate by one of the research team. It will be made clear to potential participants that participation is voluntary.

Nurses, allied health professionals, professional, administrative and support staff: These staff will be recruited via their organisations. Each children's hospital will be provided with information about the study, and asked to circulate a call for volunteers to participate via their preferred channel (e.g. email lists, newsletters). Potential participants will be asked to email the research team. The research team will then provide a detailed participant information sheet about the study and a copy of the consent form to all interested participants.

No payment will be made to participants.

Consent

Formal written consent supported by a participant information sheet will be sought for the recorded interviews and focus groups. This will be received by the researcher conducting the interview or focus group. On initial agreement by the participant to conduct the interview or focus group they will be provided with a copy of the consent form and participant information sheet. Due to the logistics of the study there will be a period of time between initial agreement and the interview or focus group taking place. This will allow enough time to ensure the participant is entirely happy to be involved and has time to ask questions. Given the study populations, no problems with understanding the consent process are anticipated.

6 ETHICAL AND REGULATORY CONSIDERATIONS

Assessment and management of risk

The research team have all undertaken Good Clinical Practice training, and completed Disclosure and Barring Service checks. As experienced researchers in healthcare, they are experienced in managing safeguarding issues. Limits to confidentiality will be outlined in the participant information sheets,

Brewster L, *et al. BMJ Open* 2024; 14:e081056. doi: 10.1136/bmjopen-2023-081056

stating that if the participant or someone else is at significant risk of harm, the researcher will break confidentiality and speak to a member of the National Paediatric Accelerator Programme. If possible, the researcher will inform the participant before they do this.

All participants will be given a numeric reference/ pseudonym. For the focus groups and interviews, we will anonymise the data (using pseudonyms) in RAP sheets and onwards. Any identifying details (names/ details of incidents) will be anonymised in the report/ any future publications.

Information about confidentiality and its limitations will be included in each Participant Information Sheet, and will be explained to each participant before starting the interview/ focus group. We will verbally remind participants not to share personally identifiable data in focus groups, and not to share focus group content outside the session.

The interviews and focus groups do not cover areas that are potentially sensitive, but the research team are experienced in conducting interviews and focus groups on sensitive topics. We do not anticipate risks to participating in the study. Although participants may find participating interesting, there are no direct benefits in taking part. A Lancaster University Health and Safety assessment has been completed to ensure safety of the research team and participants.

Research Ethics Committee (REC) and other Regulatory review & reports

This study does not require NHS REC approval; however it does require HRA approval via the IRAS system and ethical approval from the Lancaster Faculty of Health and Medicine Ethics Committee. This has been confirmed via the HRA queries helpline.

Before the start of the study, a favourable opinion will be sought from the Lancaster FHM REC for the study protocol, informed consent forms and other relevant documents e.g. advertisements.

Substantial amendments that require review by the REC will not be implemented until that review is in place and other mechanisms are in place to implement at site.

All correspondence with the REC will be retained.

If the study is ended prematurely, the Chief Investigator will notify the REC, including the reasons for the premature termination.

Amendments

Amendments will be managed via the FHM REC application for amendment to previously approved research form. Following approval each site will be informed of the amendment via the research and development manager in each trust.

Amendments will be discussed with the funder and in quarterly oversight meetings.

Amendments will be tracked by keeping an electronic trail of applications and responses to applications for amendments in an access controlled OneDrive folder.

The decision to make an amendment will be taken by the CI with input from the research team.

Protocol compliance

Protocol deviations will be reported in writing to the sponsor (via the Faculty of Health and Medicine REC). Protocol violation will lead to an internal review of protocol with the research team. Recurring protocol deviations will pause the study and lead to substantive changes to the protocol and re-review by the Faculty of Health and Medicine REC.

End of study

The end of the study is defined as the submission of the final report to the funder.

Data protection and participant confidentiality

All investigators and study site staff will comply with the requirements of the General Data Protection Regulations and the Data Protection Act 2018. All use of personal information during the course of the project will be in accordance with the General Data Protection Regulations and the Data Protection Act 2018. We do not expect to collect any sensitive data.

Personal data (names, job roles, email addresses and phone numbers) will be given by participants when they contact the research team to arrange interviews/ focus groups. This information will only be used to arrange interviews and focus groups, and will be stored in a password-protected spreadsheet in an access-controlled OneDrive folder for the duration of the project. This information will be held separately from the data set. Personal data will only be accessible to the research team. Consent forms will be stored electronically in an access-controlled OneDrive folder, until the end of the project. If consent is taken in paper form, these forms will be scanned and uploaded to the access controlled OneDrive folder, as soon as possible after consent is taken, and originals will be securely shredded.

Lancaster University will be the data controller. For further information about how Lancaster University processes personal data for research purposes, please visit: www.lancaster.ac.uk/research/data-protection

Three sources of data will be gathered and analysed:

1. Interview video recordings.

Because interviews will be conducted via Microsoft Teams, these interviews will be video recorded. It is not possible to only audio record in Teams, so we will need to store video, though these will not be used for analysis. Video recordings will be accessed by another member of the research team, who will listen to the interview and complete a RAP sheet (see 3 below), which will then be stored digitally. Video recordings made on Teams are automatically stored on Lancaster University's Microsoft Stream platform. Only the meeting owner (the researcher/ project administrator) has access to the recording, but will grant access to another member of the research team for analysis (see above). All video recordings will be deleted by the meeting owner within six months of the end of the project (June 2023). From June 2022, automatic storage infrastructure will change, and videos will automatically be stored in OneDrive. The same access restrictions and plans for deletion will apply.

2. Focus group audio recordings. If pandemic protocols mean that focus groups have to be conducted remotely, video recording storage/ collection will apply (see 1 above).

Focus group audio recordings will be copied to the access controlled OneDrive folder and deleted from the capture device on the day of creation or as soon as possible afterwards. This prevents accidental data disclosure and guarantees that the data are backed-up. Digital recordings will be identified with a participant number only. Audio recordings of focus groups will be destroyed within six months of the end of the project. Audio and video recordings will not be transcribed in full, but anonymised quotations identified with a participant number may be transcribed into RAP sheets.

3. RAP sheets, completed by the research team.

RAP (Rapid Appraisal) sheets will be completed electronically in Microsoft Word and stored on the access-controlled OneDrive folder. They will constitute the main source of data from human participants to be retained and will be stored for ten years as per university policy. RAP and RREAL methodologies emerge from a qualitative research tradition that recognises the need for rapid analysis of data to produce meaningful recommendations for practice in a timely manner (Vindrola-Padros et al, 2020). RAP sheets will be used for team debriefs and analysis.

The researchers may take written notes as an aide-memoire of these focus groups, and of their general impressions and reflections on any informal encounters while onsite, but these notes will not constitute a formal source of data or contain identifiable data. These notes will be destroyed (shredded) following the completion of RAP sheets for the session.

Participants will be informed that they may withdraw from the study at any time before or during the interview/ focus group, and up to two weeks following their interview/ focus group participation without giving any reason. If the request to withdraw comes after data have been anonymised, and incorporated into themes, it might not be possible for it to be withdrawn, though every attempt will be made to extract the data, up to the point of delivering the report to the funder.

Lancaster University will act as the data custodian. Day-to-day data management will be undertaken by the PI. Written/ digital data will be stored in a Onedrive folder with access limited to the project team for ten years after the end of the project. After this, it will be deleted and responsibility for data management will lie with Professor Rachel Isba as principal investigator.

Indemnity

Lancaster University legal liability cover will apply:

- to meet the potential legal liability of the sponsor(s) for harm to participants arising from the management of the research
- to meet the potential legal liability of the sponsor(s) or employer(s) for harm to participants arising from the design of the research
- to meet the potential legal liability of investigators/collaborators arising from harm to participants in the conduct of the research

No arrangement for payment of compensation in the event of harm to the research participants where no legal liability arises has been made.

Personal research devises (laptop, mobile telephone) are insured by the researcher personally.

7 DISSEMINATION POLICY

As a mandatory clause in the consultancy contract, ownership of all intellectual property and data rights for the project will rest with Sheffield Children's Hospital (SCH) (on behalf of all the Trusts participating in the project) and we will not publish any aspect of the research without permission from Sheffield Children's Hospital. SCH agree that permission will not be unreasonably withheld, but data not be disseminated or shared for reuse.

We will produce a report and presentation of findings for the funder. We will discuss possible wider dissemination of selected elements of work via publishing in an academic journal or presenting at a conference with the funder after delivering this report.

8 REFERENCES

Arksey, H., & O'Malley, L. (2005). Scoping studies: towards a methodological framework. International Journal of Social Research Methodology, 8(1), 19–32. https://doi.org/10.1080/1364557032000119616

Daudt, H. M., van Mossel, C., & Scott, S. J. (2013). Enhancing the scoping study methodology: a large, inter-professional team's experience with Arksey and O'Malley's framework. BMC Medical Research Methodology, 13(1), 48. https://doi.org/10.1186/1471-2288-13-48

Edge Health (2021) "Analysis of inequalities and "was not broughts" for outpatients appointments". (Analysis prepared for the Paediatric Accelerator inequalities group). Unpublished

Khalil, H., Peters, M., Godfrey, C. M., McInerney, P., Soares, C. B., & Parker, D. (2016). An Evidence-Based Approach to Scoping Reviews. Worldviews on Evidence-Based Nursing, 13(2), 118–123. https://doi.org/10.1111/wvn.12144

Kvale, S., & Brinkmann, S. (2014). InterViews. SAGE Publications.

Marmot, M., Allen, J., Goldblatt, P., Herd, E., Morrison, J. (2020). Build Back Fairer: The COVID-19 Marmot Review. The Pandemic, Socioeconomic and Health Inequalities in England. London: Institute of Health Equity

Morse, J. M. (2015). Critical Analysis of Strategies for Determining Rigor in Qualitative Inquiry. Qualitative Health Research, 25(9), 1212–1222. https://doi.org/10.1177/1049732315588501

Peters, M. D., Godfrey, C., McInerney, P., Soares, C. B., Khalil, H., & Parker, D. (2017). Chapter 11: Scoping Reviews. In M. Z. Aromataris E (Ed.), Joanna Briggs Institute Reviewer's Manual. The Joanna Briggs Institute. Retrieved from https://reviewersmanual.joannabriggs.org/

Silverman, D. (2015). Interpreting Qualitative Data. SAGE.

Vindrola-Padros, C., Chisnall, G., Cooper, S., Dowrick, A., Djellouli, N., Symmons, S.M., Martin, S., Singleton, G., Vanderslott, S., Vera, N. and Johnson, G.A., (2020). Carrying out rapid qualitative research during a pandemic: emerging lessons from COVID-19. Qualitative health research, 30(14), pp.2192-2204

Vindrola-Padros, C., (2021) Doing Rapid Qualitative Research. SAGE.

9. APPENDICES

Appendix 1- Required documentation

- CV R Isba
- CV J Lunn
- CV L Brewster
- CV L Brennan
- Participant Information Sheet interviews
- Participant Information Sheet focus groups
- Consent Form interviews
- Consent Form focus groups
- Email interview invitation
- Focus groups promotional material
- Interview Guide
- Focus group guide
- RAP sheet

Appendix 2 – Schedule of Procedures

- HRA Schedule of Events
- HRA Organisation Information Document
- HRA Is My Study Research Result
- HRA Does My Study Need NHS REC Approval Result

Appendix 3 – Amendment History

No amendments – first submission

Amendment No.	Protocol version no.	Date issued	Author(s) of changes	Details of changes made
