# Supplementary material for: Understanding responsibility for health inequalities in children’s hospitals in England: a qualitative study with hospital staff
Source: BMJ Open. 2024 Apr 10;14(4):e081056. doi: 10.1136/bmjopen-2023-081056 (PMC11015292; doi:10.1136/bmjopen-2023-081056)
Supplement: Supplementary data [file bmjopen-2023-081056supp002.pdf]

Children's Hospitals Inequalities Research Project

Lancaster University

Interview Schedule

Senior leaders, NEDs and doctors

Welcome and introduction - Seek consent to continue, reminder of audio-recording of interview (/video recording if on Teams), re-cap of project and plan for interview.

The interviews will explore your views on the Trust's policies and practices around health inequalities. We are interested in your perceptions, on the basis of your knowledge and experience. If you do not feel you are able to comment on any area please say so. Do you have any questions before we start?

1. What does the term 'the socio-economic inequalities in paediatric care' mean to you?
    - Prompt: an example of a socio-economic inequality in health is that children from the most deprived or poorest areas have a shorter life expectancy than people in the wealthiest areas.

2. In your experience, does deprivation have an impact on children's health?
    - Prompt: by deprivation we mean things like people living in areas with lower levels of income, employment, higher crime, less education

3. In your experience, does deprivation have an impact on whether or not children access care?
    - Prompt: What stops people from deprived areas from bringing their children to hospital for appointments and care?

4. Do you see health inequalities in the patients using this hospital?
    - What are these? How do they affect care (if not outlined above)

5. What do you see as your role in regard to health inequalities?

6. <u>Doctors only:</u> Are there any processes in place to help you, if you see health inequalities?
    - Prompt: have you received training, are there systems for flagging concerns?
    - Are there any barriers to this? Prompt: E.g. costs of care for patients, timings/ locations of appointments.

7. Does the Trust have a shared organisational view on its role in health inequalities?
    - Prompt: Are you aware of a statement by the board?

8. Are there any organisational policies in place relating to socio-economic barriers to accessing healthcare?

9. Do you think that COVID has affected socioeconomic inequalities?
    - (if yes) What is the Trust doing to address the impacts of COVID?
    - What impact, if any, has COVID had on access and quality at the Trust?

CHIRP – interview schedule – v2 - 2022-06-16

10. What does the Trust do to reduce health inequalities?
    - What could they do better/ more of?
    - Are there any barriers to this?

11. What would 'best practice' in addressing the socio-economic barriers to paediatric care look like?
    - Prompt: Have you seen evidence from elsewhere in the UK or internationally of what works well?

12. Senior leaders only: As an employer, does the Trust have a role in considering in wider society and socio-economic inequalities?

13. Senior leaders only: Any specific questions about hospital policies that have been reviewed for clarification.

Anything not covered?

Is there anything that we haven't covered in the interview that you think we should know or think about?

Closing and thanks - Thank for their time and contribution.
