# Supplementary material for: Understanding responsibility for health inequalities in children’s hospitals in England: a qualitative study with hospital staff
Source: BMJ Open. 2024 Apr 10;14(4):e081056. doi: 10.1136/bmjopen-2023-081056 (PMC11015292; doi:10.1136/bmjopen-2023-081056)
Supplement: Supplementary data [file bmjopen-2023-081056supp003.pdf]

Children's Hospitals Inequalities Research Project

Lancaster University

Focus Group Schedule

Nurses, Allied Health Professionals

Professional, administrative and support staff

Welcome and introduction - Seek consent to continue, reminder of audio-recording of the group, establish ground rules, re-cap of project and plan for focus group

The focus group will explore your views on the Trust's policies and practices around health inequalities. We are interested in your perceptions, on the basis of your knowledge and experience. Please try not to use any details which might lead someone in the group to be able to identify another staff member/ patient (e.g. their name). If you do not feel you are able to comment on any area please say so. Does anyone have any questions before we start?

1. In your experience, does deprivation have an impact on children's health?
    - Prompt: by deprivation we mean things like people living in areas with lower levels of income, employment, higher crime, less education

2. In your experience, does deprivation have an impact on whether or not children access care?
    - Prompt: What stops people from deprived areas from bringing their children to hospital for appointments and care?

3. Do you see health inequalities in the patients using this hospital?
    - What are these? How do they affect care (if not outlined above)

4. What do you see as your role in regard to health inequalities?

5. Do you know if the Trust has a policy, or a view, on its role in health inequalities?
    - (If yes) What is it? Can you say more?
    - (If no) Why do you think it does not have a policy/ view?

6. Are there any processes in place to help you, if you see health inequalities?
    - Prompt: have you received training, are there systems for flagging concerns?
    - Are there any barriers to this? Prompt: E.g. costs of care for patients, timings/ locations of appointments.

7. What does the Trust do to reduce health inequalities?
    - What could they do better/ more of?

Anything not covered?

Is there anything that we haven't covered in the focus group that you think we should know or think about?

Closing and thanks

Thank for their time and contribution.

CHIRP – focus group schedule – v2 – 2022-06-16
