# Supplementary material for: Understanding responsibility for health inequalities in children’s hospitals in England: a qualitative study with hospital staff
Source: BMJ Open. 2024 Apr 10;14(4):e081056. doi: 10.1136/bmjopen-2023-081056 (PMC11015292; doi:10.1136/bmjopen-2023-081056)
Supplement: Supplementary data [file bmjopen-2023-081056supp004.pdf]

Children's Hospitals Inequalities Research Project

Lancaster University

Study: Children's Hospitals Inequalities Research Project (CHIRP).

Researcher name	
Study location (name of hospital)	
Interview/ focus group (delete as applicable)	If focus group: number of participants =
Participant group (leadership, clinician, other)	
Examples of socio-economic inequalities	
Deprivation: impact on children's health	
Deprivation: impact on access to care	
Deprivation: visibility in their context (hospital)	
Perceived/ outlined role in health inequalities	
Awareness of policies/ policies and organisational views	
Processes in place for support	

Impact of Covid	
What the Trust currently does	
What the Trust might do more of/ better	
What stops the Trust doing more/ better	
Examples of best practice	
Clarification points (policies; SL only)	
Miscellaneous	
Other comments	
