# Supplementary material for: Understanding responsibility for health inequalities in children’s hospitals in England: a qualitative study with hospital staff
Source: BMJ Open. 2024 Apr 10;14(4):e081056. doi: 10.1136/bmjopen-2023-081056 (PMC11015292; doi:10.1136/bmjopen-2023-081056)
Supplement: Author's manuscript [file bmjopen-2023-081056.draft_revisions.pdf]

BMJ Open

BMJ Open is committed to open peer review. As part of this commitment we make the peer review history of every article we publish publicly available.

When an article is published we post the peer reviewers' comments and the authors' responses online. We also post the versions of the paper that were used during peer review. These are the versions that the peer review comments apply to.

The versions of the paper that follow are the versions that were submitted during the peer review process. They are not the versions of record or the final published versions. They should not be cited or distributed as the published version of this manuscript.

BMJ Open is an open access journal and the full, final, typeset and author-corrected version of record of the manuscript is available on our site with no access controls, subscription charges or pay-per-view fees (http://bmjopen.bmj.com).

If you have any questions on BMJ Open's open peer review process please email info.bmjopen@bmj.com

BMJ Open

Understanding responsibility for health inequalities in children's hospitals in England: a qualitative study with hospital staff

Journal:	*BMJ Open*
Manuscript ID	bmjopen-2023-081056
Article Type:	Original research
Date Submitted by the Author:	17-Oct-2023
Complete List of Authors:	Brewster, Liz; Lancaster University, Lancaster Medical School<br>Brennan, Louise; Lancaster University Faculty of Health and Medicine<br>Hindocha, Avni; Manchester University NHS Foundation Trust, Royal Manchester Children's Hospital; Lancaster University, Lancaster Medical School<br>Lunn, Judith; Lancaster University<br>Isba, Rachel; Lancaster University Faculty of Health and Medicine
Keywords:	Hospitals, Health Equity, PAEDIATRICS, PUBLIC HEALTH, QUALITATIVE RESEARCH

SCHOLARONE™
Manuscripts

I, the Submitting Author has the right to grant and does grant on behalf of all authors of the Work (as defined in the below author licence), an exclusive licence and/or a non-exclusive licence for contributions from authors who are: i) UK Crown employees; ii) where BMJ has agreed a CC-BY licence shall apply, and/or iii) in accordance with the terms applicable for US Federal Government officers or employees acting as part of their official duties; on a worldwide, perpetual, irrevocable, royalty-free basis to BMJ Publishing Group Ltd ("BMJ") its licensees and where the relevant Journal is co-owned by BMJ to the co-owners of the Journal, to publish the Work in this journal and any other BMJ products and to exploit all rights, as set out in our licence.

The Submitting Author accepts and understands that any supply made under these terms is made by BMJ to the Submitting Author unless you are acting as an employee on behalf of your employer or a postgraduate student of an affiliated institution which is paying any applicable article publishing charge ("APC") for Open Access articles. Where the Submitting Author wishes to make the Work available on an Open Access basis (and intends to pay the relevant APC), the terms of reuse of such Open Access shall be governed by a Creative Commons licence – details of these licences and which Creative Commons licence will apply to this Work are set out in our licence referred to above.

Other than as permitted in any relevant BMJ Author's Self Archiving Policies, I confirm this Work has not been accepted for publication elsewhere, is not being considered for publication elsewhere and does not duplicate material already published. I confirm all authors consent to publication of this Work and authorise the granting of this licence.

For peer review only

UNDERSTANDING RESPONSIBILITY FOR HEALTH INEQUALITIES IN CHILDREN'S HOSPITALS IN ENGLAND: A QUALITATIVE STUDY WITH HOSPITAL STAFF

ABSTRACT

Objectives: To understand how children's hospitals in England view their responsibility to reduce health inequalities for the children and young people who access their services.

Design: Exploratory qualitative study.

Setting: Nine children's hospitals in England.

Participants: 217 members of staff contributed via interviews and focus groups. Staff were represented at all levels of the organisations, and all staff who volunteered to contribute were included in the study.

Results: While reducing health inequalities was seen as something that should be 'everyone's business', the deeply entrenched and intersectional nature of health inequalities sometimes meant that they were complex to tackle, with no clear impetus to specific actions, and could be framed as 'no-one's responsibility'. Within a complex health and social care system, there were many potential actors who could take responsibility for reducing health inequalities, and staff often questioned whether it was the role of a children's hospital to lead these initiatives. However, all of the children's hospitals were taking some action to reduce health inequalities. Many staff felt there was an obligation to intervene to ensure that children and young people receiving hospital treatment (and their caregivers) were not further disadvantaged by, e.g. food poverty.

Conclusions: Broadly speaking, senior leaders were clear about their organisational role in reducing health inequalities where they impacted on access and quality of care, but there was some uncertainty about the perceived boundaries of responsibility. This led to fragility in the sustainability of activity, and a lack of joined-up intervention. Most hospitals were forging ahead with activity, considering that it was more important to work to overcome health inequalities rather than debate whose job it was.

STRENGTHS AND WEAKNESSES:

- This is the first study to investigate staff's views on health inequalities in children's hospitals in England.
- The sample size of over 200 participants created a substantial dataset for qualitative analysis, which indicates that the findings are robust and transferrable.
- Participants were self-selecting and so may have volunteered to participate because they had a particular interest in sharing views on health inequalities.

For peer review only

UNDERSTANDING RESPONSIBILITY FOR HEALTH INEQUALITIES IN CHILDREN'S HOSPITALS IN ENGLAND: A QUALITATIVE STUDY WITH HOSPITAL STAFF

INTRODUCTION

Definitions of health inequalities vary, but there is broad agreement that these inequalities are unfair and avoidable (1). These inequalities are also systemic or structural rather than purely individual (2,3). Health inequalities are experienced on a social gradient, with those who are more socioeconomically disadvantaged facing greater health challenges, in experiences of illness and access to healthcare (4). In the UK, Michael Marmot and the Institute of Health Equity have been key contributors to driving forward an agenda that recognises the impact of health inequalities on children and young people (5,6). In 2010, the publication of the Marmot Review 'Fair Society, Healthy Lives' (7) set out a framework for action that emphasised how these inequalities accumulate across the lifespan. The recommendations prioritised children as a population group requiring early and meaningful intervention to prevent health inequalities becoming entrenched. This review was updated in 2020, presenting evidence that the gap in health status and outcomes between affluent and socio-economically deprived areas had increased rather than decreased (8).

Children living in areas of high deprivation are more likely to have worse health outcomes (9) and long-term conditions like asthma (10). Access to care also differs, with those from the most deprived areas being less likely to be brought to outpatient appointments (11). Health inequalities therefore represent a tangible issue that needs to be addressed internationally, and have a direct impact on the effectiveness of health service provision.

In the UK, healthcare is publicly-funded and provided free at the point of use, via the National Health Service (NHS). NHS organisations have a statutory duty with regards to specific equality domains defined by the Equality Act 2010 (12), which outlined protected characteristics (e.g. age, sex, race) but there had not been a specific duty on NHS organisations to intervene with regard to health inequalities caused by socio-economic deprivation until the Health and Social Care Act 2012 (13), which introduced the first legal duties relating to health inequalities. Health inequalities are also part of several subsequent national strategies, e.g. the NHS Long Term Plan (14) and the NHS People Plan (15). Both socio-economic and health inequalities are thought to have increased since the SARS-CoV-2/COVID-19 pandemic, impacting health outcomes, with longer waiting times for elective care. Greater attention has been given to reducing inequalities because of this increase, and the impact on children and young people has been identified as a particular focus for intervention, via initiatives such as NHS England's 'Core20PLUS5' approach (16). This recognises that inequalities are intersectional, focusing on the most deprived 20% of the population, prioritising inclusion, e.g. of people from ethnic minority communities, and identifying clinical areas of focus, e.g. asthma, diabetes, and epilepsy.

Within the NHS, children's hospitals provide specialist paediatric care to children and young people, typically up to the age of 16. This provision can be as a stand-alone hospital for children, or as part of a wider organisational structure (NHS trust). There is great diversity amongst service provision because of these organisational structures, but in the main children's hospitals offer services (e.g. emergency and acute general medicine, community and outpatient care) to their local population, and then specialist services to patients with more complex medical needs (e.g. paediatric cardiac surgery) across a regional or national catchment. Children and young people and their caregivers may therefore travel a great distance to access these more specialist services.

For peer review only

This study aimed to understand how children's hospitals in England view their responsibility to reduce health inequalities for the children and young people they serve.

METHODS

Qualitative data were collected via interviews and focus groups, working across nine children's hospitals in England. The qualitative approach was selected in order to describe participants' understanding of current policy and practice around socio-economic inequalities and their impact on healthcare, rather than hypothesising about, or predicting, responses (17).

Rapid Research Evaluation and Appraisal (RREAL) methodologies (18) were used to collect and analyse data; these methodologies emerge from a qualitative research tradition that recognises the need for rapid data analysis to produce meaningful recommendations for practice in a timely manner. The full process had four stages, in which data were collected, recorded, synthesised, and analysed to produce key recommendations. Using multiple population groups (senior leaders, doctors, nurses, allied health professionals, and professional/ support staff), multiple data sources (interviews and focus groups), and multiple experienced researchers, allowed us to ensure rigour by triangulating insights (19).

Recruitment and participants

Hospitals sampled are all members of the Children's Hospital Alliance, which commissioned a broader research project to exploring the roles that their member organisations can play in reducing socio-economic barriers to paediatric care, and in understanding and developing best practice to do this. The hospitals varied in geographic location, percentage of local population living in the most 20% of deprived communities, organisational size and structure, and services offered to the paediatric population.

Recruitment was organised by the nine participating organisations. The diversity of organisations meant that we were guided by each one as to the most appropriate method to recruit participants. In total, 217 members of staff were included across the sites (table 1).

Organisation	Interviews	Focus Groups/ conversations	Total number of participants per site
Site A	9	4	28
Site B	4	4	33
Site C	7	2	15
Site D	10	4	22
Site E	4	2	19
Site F	11	15	53
Site G	6	1	11
Site H	5	2	20
Site I	5	2	16
**TOTAL**	**61**	**39**	**217**

Table 1: Staff participants across nine NHS organisations

Staff were represented at all levels from senior executives to housekeeping staff. To maintain anonymity when presenting data we refer to broad three categories of staff:

- Leadership - executive and non-executive directors, anyone involved in the management of initiatives concerned with health inequalities;

For peer review only

- Clinical - doctors, nurses, and allied health professionals (e.g. physiotherapists, occupational therapists);
- Professional and support staff – e.g. receptionists, porters, security, housekeeping.

Data collection

The research team consisted of two academics with expertise in applied qualitative health research, and three research-active public health specialists (one consultant, one registrar and one speciality trainee). We conducted semi-structured interviews with senior leaders and doctors with an interest in health inequalities across nine organisations. Interviews lasted between 30 and 60 minutes. A structured topic guide (see supplementary materials) was used to provide an initial focus and allow for comparability between respondents, while also allowing for flexibility and individuality in the responses given. Interviews were recorded via Microsoft Teams. In total, 61 interviews were completed, and number of interviews per organisation ranged from 4-11.

We conducted focus groups and had informal conversations with clinical, professional and support staff. The majority of focus groups were audio recorded, but for informal conversations, brief written notes with no identifiable details were made and then team members audio-recorded a debrief session to provide an overview of the conversations. One or two focus groups were conducted per organisation, lasting around 60 minutes per group. Number of participants ranged from 3-10 members of staff. A structured topic guide (see supplementary materials) provided focus for discussion. One site advised that informal 'walk around' conversations were more appropriate to engage staff; these discussions were also structured using the same topic guide. All nine organisations were visited for one or two days, meaning additional debrief recordings were often comprehensive and detailed. In total, 39 focus groups and informal discussions were completed. Data were collected between January and June 2023.

Ethical approvals

Ethical approval was granted by FHM Research Ethics Committee Lancaster University on 16 June 2022 (ref: FHM-2022-0844-RECR-3). Health Research Authority approval was granted on 24 August 2022 (ref: IRAS315113 and 22/HRA/3123) and capacity and capability to participate was confirmed by each organisation.

Data analysis

Data analysis was conducted in several stages, informed by the RREAL methodology for rapid assessment procedures (RAP) (20).The RAP process is considered an appropriate method to conduct rigorous and robust qualitative data analysis in a short amount of time. It is predominantly team-based and collaborative in nature, and conducted as an iterative process alongside data collection. Data are independently reviewed by multiple team members, before being brought together in a group session for further analysis. RAP is particularly suitable for this type of research, which aims to synthesise diverse perspectives on a defined topic, and is focused on generating useful learning that can be actioned. While it was felt that 'data saturation' (21) was being reached at organisations, it is evident that results are intrinsically linked with the sample of people that we spoke to at each hospital.

Following the first stage of data collection, three further stages (multiple RAP sheet generation, individual consolidation and group confirmation) completed the RREAL process. After each interview, focus group, or debrief, a structured RAP sheet (supplementary materials) outlining the main themes was completed by the researcher leading the data collection. One or more members of the research team then listened to the recording, and

For peer review only

independently completed a RAP sheet. A minimum of two standardised RAP sheets was completed for each interview/focus group, and used to guide our reflexive analysis process. In total, 171 RAP sheets were generated. Researchers then read and reviewed all the RAP sheets generated, and completed independent informal notes to support synthesis. Two members of the team independently generated further 'site-specific' RAP sheets, providing an overview of key themes at each organisation. This synthesis process focused on considering the specific context of each organisation as well as the broader project research questions.

We then conducted day-long group debriefing and analysis sessions, in which we focused on identifying key themes from RAP sheets. This process led to the creation of brief data summaries that were then used iteratively for cross-case comparison. The whole team contributed to these sessions, which aimed to establish consensus, while still being sensitive to diversity of views and experiences in different organisations. Each analysis session ended with confirmation and agreement of key themes.

RESULTS

From our qualitative dataset, including the perspectives of 217 staff across nine organisations, two main themes were identified that help to understand how children's hospitals in England view their responsibility to reduce health inequalities for the children and young people using their services. The first was that staff saw reducing health inequalities as 'everyone's business' and that no child should experience inequalities in care or outcomes because of their socio-economic status. This was particularly visible in terms of the activities that hospitals were undertaking in this space, regardless of national steer and guidance.

However, the second theme – whilst also recognising that health inequalities were deeply entrenched and intersectional – suggested that these inequalities were seen as 'no-one's responsibility' or 'not part of core business' for a children's hospital. Within a complex health and social care system, there were many potential actors who could take responsibility for reducing health inequalities, and staff often questioned whether it was the role of a children's hospital to lead these initiatives rather than treating the presenting illness. This led to some fragility in the sustainability of initiatives.

Nevertheless, all of the children's hospitals were taking some action to reduce health inequalities. Many staff felt there was an obligation to intervene to ensure that children and young people receiving hospital treatment (and their caregivers) were not further disadvantaged by, e.g. food poverty.

Health inequalities as 'everyone's business'

The theme of preventing health inequalities as something that should be 'everyone's business' was observed in discussion of the strategy and day-to-day activities of each organisation. All participants were asked about their knowledge of organisational policies and strategies that related to health inequalities. Over half of the nine organisations already had a health inequalities strategy in place, although for larger organisations who also offered adult provision, this was an organisation-wide strategy rather than child-focused. The remaining organisations all discussed making progress towards a strategy. The majority of organisations had a health inequalities steering group and/or forum. More than half had a data dashboard, or made reference to data analysis activity, and this was used to try to identify patients at risk of not attending appointments. This activity was led at an organisational level, rather than linked to national strategy.

For peer review only

Some participants were clear about the organisational strategy around health inequalities, but others were less certain what the overall vision was, or what activities connected to it. In some organisations, there was a named individual with responsibility for health inequalities, but this was not universal, and often, people working on the 'shop floor' were less familiar with the strategic direction. We found that awareness varied in terms of staff knowledge of strategy and policy around health inequalities. While this finding was heavily dependent upon the sample asked, each site had some staff who were unaware of strategy or activity. It became clear that there was a lack of clear communication about work on health inequalities.

> *'It's frustrating as a clinician… I asked my leads and they said they didn't know, there's all these layers of people who didn't know… eventually I found out about a steering group. But it's not affecting my practice what they're doing [about reducing inequalities].* (Clinical, site B)

This lack of communication was one example of how work to reduce health inequalities was fragile. It was often led by keen individuals, and so while health inequalities were seen as something that should be a focus for activities ('everyone's business'), this could be more at the level of a vision that informed activities rather than something with concrete key performance indicators (KPIs).

Consideration of reduction of health inequalities was clearly visible in interventions led by the organisations. There were several common forms of support across organisations, almost all of these focused on meeting day-to-day basic needs. These included cheap food offers, free food for breastfeeding mothers, free sanitary products, parking/travel reimbursement and hospital transport for specific identified groups, accommodation, and interpreters. While available in the majority of organisations, these basic offers were not universal across all sites and specialties. Other initiatives which occurred less frequently included food vouchers, a universal patient transport offer, and free food on wards for household members.

As well as strategic direction and organisationally-initiated formal support for families, a second, more hidden layer of support was present in many of the organisations. This informal system was largely driven by individuals, often at nursing or domestic support level, and showed how reducing health inequalities permeated into every level of work. Stories of housekeeping staff taking washing from families, buying caregivers coffees and food, and giving leftover food to families were frequent. Similarly, nursing staff discussed ways to order additional food when they noticed someone was in need. In some hospitals, ward managers had access to a budget that funded food vouchers and taxis. This support was partly dependent upon motivated and caring individuals.

> *'I think the people that I speak to, and I speak to a broad range, it's actually part of their core value rather than something that they think would make a good headline. And I think that resonates throughout the entire organization that they would be doing this even if no one was watching them do it.'* (Leadership, site F)

Some staff felt that there needed to be KPIs around health inequalities to encourage prioritisation of resource to this area of activity. With no current metrics around health inequalities, it becomes one of the 'nice to haves' rather than an essential.

> *'We don't have standards for the level of support for our families and we should do shouldn't we?'* (Leadership, site D)

For peer review only

'We can't allow health inequalities to become a luxury activity for organisations that happen to have the resources.' (Leadership, site G)

Some staff explained that while health inequalities were not new, the pandemic had highlighted inequalities in outcomes and provided evidence (and therefore understanding) of the health inequalities that passionate members of staff had been trying to explain previously.

'I'm sure if you'd have talked about some of this stuff five years ago people wouldn't have understood as much as they do now.' (Clinical, site A)

It was felt that the pandemic had also shone a focus on inequalities, kickstarting projects such as analysis of data on potential inequalities.

'There's variation in how long certain groups are waiting for care… we've observed in our analysis those with the lowest income wait on average six years longer. Children and young people with a learning disability wait longer to complete their treatment. Both of those factors were there before Covid, but now we have done the analysis and are looking at how we can address it.' (Leadership, site B)

Most of the leadership and clinical staff we interviewed were vocal about the way that health inequalities were their business, and affected their day-to-day provision of healthcare.

'Because if they don't have a good start, they're not gonna have a long and happy life. So they will have poor diet. Doesn't matter what people tell you. It is quite expensive to eat well, so they will have increased diabetes. They will have increased obesity. They'll have all the pitfalls of living in a cold, damp house. So they'll have worse asthma.' (Leadership, site F)

'Making people realise that being curious about why a certain child keeps coming back is just as much my responsibility as a doctor as it is to prescribe the Ventolin Inhaler.' (Clinical, site G)

This idea of 'prevention rather than cure' was epitomised in several examples offered of activity to reduce health inequalities. For example, one clinician discussed how the impacts of having a domestic violence prevention service piloted in their Paediatric Emergency Department might have wide-ranging impacts for children and young people attending the department, but that those effects might be seen in educational attainment in a generation's time rather than being immediately visible.

Health inequalities as 'no-one's responsibility'

Although, broadly speaking, members of leadership teams were clear about their role in overcoming socio-economic inequalities where they impacted on access and quality of care, there was some uncertainty about the perceived boundaries of responsibility with other institutions (e.g. local government, other healthcare services). Some staff saw that leading the health inequalities agenda was not the role of the hospital, and there was an awareness that other services such as local authorities, public health, schools, community care, and general practice also had a role to play.

'So whose responsibility is it to do this? One answer is everyone's responsibility. Another answer is not ours. And our job is here to see and treat people in front of us. That is one response. And you think well, there are public health teams around. There's lots of stuff. Should we be out

For peer review only

there in the city centre running our campaigns about how to brush teeth? Or should we put a dentist in theatre doing the dental extractions? That's the choice.' (Leadership, site F)

Staff at all levels felt that a key barrier to addressing health inequalities action was the lack of national and local priority given to the subject. Senior executives were honest that priority was often given to the national targets such as waiting lists and finances.

'It's not the Government's priority. No government is interested in long-term gain. They're interested in waiting lists and how they got better during their jurisdiction.' (Leadership, site D)

Although some senior staff spoke about working as part of a system, staff members at all levels, and across all organisations, spoke about their frustrations in not feeling part of the system. This was seen as a barrier to advancing the inequalities agenda for children and young people.

'It's being holistic as a system that we're not very good at doing. Because we are separate to social services and we're separate to schools, we don't think of 'children's' as a system yet.' (Leadership, site D)

This was compounded by the inability of IT systems to link up the health and social care system.

'No one has time or capacity to make these sort of links happen. It is just not at the top of their agenda to try and get everyone equitable access in one area. You can see a massive discrepancy across the region and actually it does have an impact on death rates in our service.' (Clinical, site I)

For some organisations, their status within the community as a 'trusted brand' meant that they felt it was within their remit to intervene, regardless of their 'responsibility.' Children's hospitals were seen as well-placed to lead on and communicate upon health inequalities to the wider public.

'We've got a trusted brand and … it's well known, it's well understood. And if we went walking up and down the high street with donation boxes, people go oh yeah, I'll give money to the children's hospital.' (Leadership, Site F)

The picture was more mixed in terms of the views of the broader staff cohort. Some staff saw support for overcoming health inequalities as part of their role, but others felt it was not a priority, or felt less confident in asking questions about deprivation and need. Most health inequalities work was being driven by passionate individuals, making it unsustainable if these people leave. These champions were a positive, in terms of driving action, but their impact was limited by the way in which health inequalities were not part of core business or communicated within their organisations, and often went unnoticed by staff at leadership level. The lack of embeddedness meant that even if the aim was for every member of staff to take responsibility for tackling the health inequalities they encountered, they were not held responsible for delivering this outcome.

A smaller number of staff were concerned about the overall expense of initiatives such as free food and transport, particularly if they were promoted widely. They did not see support for patients as a key role for the organisations, and highlighted the potential for initiatives to

For peer review only

be inappropriately accessed by people who did not need the support. They also recognised the impact of increased workload, for example if families were fed alongside patients, and noted that this could be time-consuming. It became clear that for inequalities to become part of core business, a significant shift was required.

DISCUSSION

This paper has presented findings from a large-scale qualitative study aiming to understand how children's hospitals in England view their responsibility to reduce health inequalities for the children and young people who access their services. Key findings were that while reducing health inequalities was broadly seen as important to these organisations, there were a number of barriers to activity, including a lack of agreement about the scope and remit of intervention, and whose priority it was to lead on these interventions. There was little national guidance to support decisions, but organisations could see the consequences of a lack of intervention on a daily basis. These discussions about when, where, and how to intervene often centred on questions around best use of available resource, and how impact of resource use could not be evidenced or tied to organisational key priorities.

Strengths of this study are that it is, to our knowledge, the first study to investigate staff's views on health inequalities in children's hospitals in England or elsewhere. The sample size of over 200 participants created a substantial dataset for qualitative analysis, which indicates that the findings are robust and transferrable. Our dataset can therefore be considered a rich and robust representation of views within the nine organisations we worked with. Weaknesses include that participants were self-selecting and so may have volunteered to participate because they had a particular interest in sharing views on health inequalities.

Rapid qualitative methods were used, rather than full transcription. This may be considered a weakness, but has been accounted for in the rigorous analysis process. There is a growing consensus that full transcription of audio-recordings does not necessarily increase robustness of analyses. Due to the remit of the funding for this research, it was not possible to involve patients or the public in the design, conduct, reporting, or dissemination plans of our research. This means we were also not able to speak to children, young people and families as part of this research, meaning their voices are represented by others within this dataset. Future research should address these questions around how health inequalities impact on healthcare from the perspective of children and young people in particular.

As identified in a recent scoping review protocol (22), there are few academic journal papers that outline activities and interventions conducted by children's hospitals to reduce health inequalities, and more evidence can be found in the grey literature (defined as sources of literature that have not been formally published). Despite the acknowledged importance of reducing health inequalities for children and young people, little research has been conducted in this area, and this is the first that has sought to understand how NHS organisations and staff perceive their role. This paper therefore makes an important contribution to understanding activities to reduce health inequalities and how hospital staff at all levels may contribute to these activities. A relevant recent review (23) aimed to outline 'the importance of healthcare workers advocating for structural and high-level policy change to address the deep-rooted societal problems that cause child poverty' by proposing a framework to address health inequalities experienced by children. Its broad conclusions support our more detailed account of the entrenched and intersectional nature of health inequalities and how they might be addressed within a children's hospital context.

For peer review only

CONCLUSION AND RECOMMENDATIONS

While there is some clarity for organisations about their role in reducing health inequalities where they impacted on access and quality of care, there remains uncertainty about tackling health inequalities around the wider determinants of health, and the perceived boundaries of responsibility with other organisations within the wider health and social care system. In this study, most hospitals were forging ahead with activity, considering that it was more important to work to overcome health inequalities rather than debate whose job it was. While this is laudable, and the contribution of passionate individuals should not be underestimated, it does not position health inequalities clearly as part of children's hospitals everyday work and so the sustainability of interventions is not guaranteed. The needs of children and young people require much greater attention and stronger, clearer policy at a national level.

For peer review only

REFERENCES

1. McCartney G, Popham F, McMaster R, Cumbers A. Defining health and health inequalities. Public Health [Internet]. 2019;172:22–30. Available from: https://www.sciencedirect.com/science/article/pii/S0033350619301076
2. Whitehead M. The concepts and principles of equity and health. Int J Health Serv. 1992;22(3):429–45.
3. Graham H. Social Determinants and Their Unequal Distribution: Clarifying Policy Understandings. Milbank Q [Internet]. 2004 Mar 1;82(1):101–24. Available from: https://doi.org/10.1111/j.0887-378X.2004.00303.x
4. Donkin AJM. Social Gradient. In: The Wiley Blackwell Encyclopedia of Health, Illness, Behavior, and Society [Internet]. 2014. p. 2172–8. Available from: https://doi.org/10.1002/9781118410868.wbehibs530
5. Marmot M. The health gap: The challenge of an unequal world. London: Bloomsbury; 2015.
6. Marmot M. Social determinants of health inequalities. Lancet. 2005;365(9464):1099–104.
7. Marmot M, Allen J, Goldblatt P, Boyce T, McNeish D, Grady M, et al. Fair society, healthy lives: The Marmot Review [Internet]. Vol. 0. London; 2010. Available from: http://dx.doi.org/10.1016/j.puhe.2012.05.014
8. Marmot M, Allen J, Boyce T, Goldblatt P, Morrison J. Health Equity in England : The Marmot Review Ten Years on. London; 2020.
9. Lai ETC, Wickham S, Law C, Whitehead M, Barr B, Taylor-Robinson D. Poverty dynamics and health in late childhood in the UK: evidence from the Millennium Cohort Study. Arch Dis Child [Internet]. 2019 Nov 1;104(11):1049 LP – 1055. Available from: http://adc.bmj.com/content/104/11/1049.abstract
10. Wickham S, Anwar E, Barr B, Law C, Taylor-Robinson D. Poverty and child health in the UK: using evidence for action. Arch Dis Child. 2016 Aug;101(8):759–66.
11. French LR, Turner KM, Morley H, Goldsworthy L, Sharp DJ, Hamilton-Shield J. Characteristics of children who do not attend their hospital appointments, and GPs' response: a mixed methods study in primary and secondary care. Br J Gen Pract. 2017 Jul;67(660):e483–9.
12. HM Government. Equality Act [Internet]. 2010. Available from: https://www.legislation.gov.uk/ukpga/2010/15/contents

13. HM Government. Health and Social Care Act 2012 [Internet]. 2012. Available from: https://www.legislation.gov.uk/ukpga/2012/7/contents/enacted

14. NHS England. The NHS Long Term Plan [Internet]. 2019. Available from: https://www.longtermplan.nhs.uk/publication/nhs-long-term-plan/

15. NHS England. WE ARE THE NHS: People Plan for 2020/2021 - action for us all [Internet]. London; 2020. Available from: https://www.england.nhs.uk/ournhspeople/

16. NHS England. Core20PLUS5 – An approach to reducing health inequalities for children and young people [Internet]. 2023 [cited 2023 Aug 9]. Available from: https://www.england.nhs.uk/about/equality/equality-hub/national-healthcare-inequalities-improvement-programme/core20plus5/core20plus5-cyp/

17. Kvale S, Brinkmann S. InterViews: learning the craft of qualitative research interviewing [2nd edition]. London: Sage; 2009.

18. Vindrola-Padros C, Chisnall G, Cooper S, Dowrick A, Djellouli N, Symmons SM, et al. Carrying Out Rapid Qualitative Research During a Pandemic: Emerging Lessons From COVID-19. Qual Health Res. 2020 Dec;30(14):2192–204.

19. Morse JM. Critical Analysis of Strategies for Determining Rigor in Qualitative Inquiry. Qual Health Res. 2015 Sep;25(9):1212–22.

20. Vindrola-Padros C. Doing Rapid Qualitative Research. London: Sage Publication; 2021.

21. Charmaz K. Constructing Grounded Theory: a practical guide through qualitative analysis. London: Sage; 2006.

22. Brennan L, Brewster L, Lunn J, Egboko F, Stres DP, Patel P, et al. How do hospitals address health inequalities experienced by children and young people: a grey literature scoping review protocol. BMJ Open [Internet]. 2023 Apr 1;13(4):e071682. Available from: http://bmjopen.bmj.com/content/13/4/e071682.abstract

23. Lee AR, Kingdon CC, Davie M, Hawcutt D, Sinha IP. Child poverty and health inequalities in the UK: A guide for paediatricians. Arch Dis Child. 2022;108(2):94–101.

For peer review only

FULL/LONG TITLE OF THE STUDY

Children's Hospitals Inequalities Research Project

SHORT STUDY TITLE / ACRONYM

CHIRP

PROTOCOL VERSION NUMBER AND DATE

V1.0 21 April 2022

RESEARCH REFERENCE NUMBERS

**IRAS Number:**	315113
**FUNDERS Number:**	SCH5628 Sheffield Children's Hospitals NHS Foundation Trust (SCH)<br>National Paediatric Accelerator

For peer review only - http://bmjopen.bmj.com/site/about/guidelines.xhtml

STUDY PROTOCOL

Children's Hospitals Inequalities Research Project

1 BACKGROUND

This research will investigate how the ten children's hospitals in the Children's Hospital Alliance (England) view their responsibility to reduce socio-economic health inequalities (e.g. income, location, employment, education, cultural and religious barriers) and how these translate into actions. As part of the National Paediatric Accelerator Programme, we have been asked to look at organisational policies (collect documents) and talk to different groups of staff (in interviews and focus groups) about inequalities experienced by patients, how these affect care (e.g. by being able to attend appointments) and to identify recommendations to develop good practice to ultimately reduce the number of 'Was not Broughts' (the children's equivalent of "Did not attend"), and improve care.

This study will collect qualitative data via interviews and focus groups, which will take place online or at the participants' workplaces. NHS staff participants will be recruited via their organisations. They will include members of the senior leadership team (e.g. executive/ non-executive director plus any other relevant senior leader depending on the structure of the organisation), doctors with an interest in health inequalities, nursing and allied health professional staff (e.g. physiotherapists, occupational therapists), and professional, administrative and support staff (e.g. hospital teachers, play leaders, secretaries, receptionists, porters, security, cleaners).

2 RATIONALE

The ten children's hospitals involved in the study have been collaborating through the National Paediatric Accelerator Programme to support elective recovery, share best practice and reduce health inequalities. As a group, the organisations are seeking to develop understanding as to how acute trusts working together, can improve access to quality paediatric healthcare. The work has included looking at the inequitable impact that the SARS-CoV-2/COVID pandemic has had on the health and wellbeing of children from the poorest backgrounds. In particular, focus has been on the disproportionate impact that deprivation has had on the ability of children from different socioeconomic backgrounds to access healthcare. Children from the most deprived decile in the index of multiple deprivation are more than twice as likely to not be brought to their outpatients' appointments (Edge Health, 2021) than those from the least deprived decile.

It is relatively new for NHS trusts to think about, and seek to take action on, health inequalities relating to deprivation. In considering its role in inequalities, the NHS is most familiar with its statutory role with regards to some specific equality domains. The Equality Act 2010 defines several protected characteristics (i.e. age, sex, race, disability, sexual orientation, gender reassignment, marriage and civil partnerships, pregnancy and maternity, religion or belief discrimination). By comparison with the protected characteristics, there is no specific duty on NHS organisations to intervene with regard to deprivation. However, the Health and Social Care Act 2012 introduced the first legal duties about health inequalities. Health inequalities are also part of several national strategies, such as the NHS Long Term Plan and the NHS People Plan. Initial analysis suggests that that socio-economic factors can have significant impacts on the ability of children and their parents, and recent analysis suggests that the situation has worsened during the pandemic (Marmot et al, 2020).

This research is part of the National Paediatric Accelerator Programme, which includes the remit to reduce the barriers to families preventing them from attending appointments with a view to ultimately reducing the number of 'Was not Broughts', which will support the elective recovery plan for Paediatrics. The research project will explore the roles that these ten trusts can play in reducing socio-economic barriers to paediatric care, and in understanding and developing best practice to do this.

Literature review

As part of the research project, we will conduct a literature-based analysis of the variation in governance approaches and policies around inequalities. This will include documents e.g. policies

For peer review only - http://bmjopen.bmj.com/site/about/guidelines.xhtml

from the ten partner hospitals, published literature including how inequalities are experienced by children and young people in the UK, regional variation, impact of the SARS-CoV-2/COVID19 pandemic, grey literature around inequalities experienced by CYP, including identification of any global exemplars of how to reduce health inequalities.

3 RESEARCH QUESTION/AIM(S)

- How do the ten children's hospitals in the Children's Hospital Alliance (England) view their responsibility to reduce socio-economic health inequalities (e.g. income, location, employment, education, cultural and religious barriers)?
- How does this view of the potential responsibility to reduce socio-economic health inequalities translate into actions in the ten children's hospitals in the Children's Hospital Alliance (England)?
- Is there variation within governance approaches and policies across the ten children's hospitals in the Children's Hospital Alliance (England)?
- Are there areas where there are unmet needs for initiatives to reduce socio-economic health inequalities, either generally or due to changes in service delivery since the pandemic?
- How do the ten children's hospitals in the Children's Hospital Alliance (England) understand their role in relation to socio-economic inequality (e.g. inequalities in income, location, employment, education) where this impacts healthcare access and quality?
- How do the ten children's hospitals in the Children's Hospital Alliance (England) seek to mitigate the impacts of socio-economic inequality on healthcare?
- What are the barriers and enablers to initiatives that mitigate the impacts of socio-economic inequality on healthcare?
- What is perceived as best practice in attempts to mitigate the impacts of socio-economic inequality on healthcare?

Objectives

The objectives identified for the project are:

- Identify variation within governance approaches and policies across the ten children's hospitals in the Children's Hospital Alliance (England), summarising to help facilitate shared learning, by analysing organisational policies.
- Identify areas where there is unmet need for initiatives to reduce socio-economic health inequalities, either generally or due to changes in service delivery since the pandemic, by analysing organisational policies.
- Explore how the ten children's hospitals in the Children's Hospital Alliance (England) understand their role in relation to socio-economic inequality (e.g. inequalities in income, location, employment, education) where this impacts healthcare access and quality, through interviews and/or focus groups with various groups of staff.
- Consider how the ten children's hospitals in the Children's Hospital Alliance (England) are seeking to mitigate the impacts of socio-economic inequality on healthcare, including exploring what barriers and enablers exist, and what is perceived as best practice, through interviews and/or focus groups with various groups of staff.

For peer review only - http://bmjopen.bmj.com/site/about/guidelines.xhtml

Outcome

The primary outcome will be a report to the funder, which contains practical recommendations about the roles that the ten children's hospitals in the Children's Hospital Alliance (England) can play in reducing socio-economic barriers to paediatric care, and in understanding and developing best practice to do this.

4 STUDY DESIGN and METHODS of DATA COLLECTION AND DATA ANALYIS

This project will collect qualitative data via interviews and focus groups, and conduct a scoping review of literature. We will work across the ten partner children's hospitals in the Children's Hospital Alliance (England). We aim to recruit participants as described from each site, as per the funder's aim, but are aware that this may not be possible within the available time. Our aim is to work across all ten locations, with a view to accessing some participants from each category in a minimum of six locations.

The qualitative approach has been selected in order to describe participants' understanding of current policy and practice around socio-economic inequalities and their impact on healthcare, rather than hypothesising about or predicting responses (Kvale & Brinkman, 2014). The qualitative study will gather information and key themes by using semi-structured interviews and focus groups to explore the participants' knowledge of how socio-economic inequalities are considered and managed within their organisation. Rapid Research Evaluation and Appraisal (RREAL) methodologies (Vindrola-Padros, 2021) will be used to analyse data; these methodologies emerge from a qualitative research tradition that recognises the need for rapid analysis of data to produce meaningful recommendations for practice in a timely manner.

Scoping Review: an analysis of the variation in governance approaches and policies around inequalities in children's hospitals to identify areas of best practice.

Academic and grey literature will be accessed by a rapid scoping review. Scoping reviews are used "to map rapidly the key concepts underpinning a research area and the main sources and types of evidence available" (Arksey & O'Malley, 2005) and can be particularly useful when the literature is disparate and has not been previously mapped (Daudt, van Mossel, & Scott, 2013). Recent work has clarified best practices for scoping reviews (Khalil et al., 2016; Peters et al., 2017). From this review, key works will be identified which will be used in analysis of data. This will include key policy documents from the trusts, alongside a grey literature search for national and international secondary healthcare providers to identify areas of best practice.

Qualitative approach: interviews

One interview of approximately 30-40 minutes will take place with each participant (members of the senior leadership team and doctors with an interest in reducing health inequalities) to address the research questions. Informed consent will be given at the start of the interview. We will aim to interview a minimum of six members of the senior leadership team and a minimum of six doctors. We will interview a maximum of thirty members of the senior leadership team and a maximum of thirty doctors. We expect a realistic sample size to be 10-15 members of the senior leadership team and 10-15 doctors.

The interviews will be conducted via telephone or online via Microsoft Teams. If preferred by participants and feasible for the research team, interviews may be conducted face to face. All participants taking part in the interviews must be able and willing to give informed consent.

A draft topic guide for the interviews has been created to guide discussion and maximise relevance and appropriateness. The topic guide will be used in the interviews to provide an initial structure and allow for comparability between respondents, while also allowing for flexibility and individuality in the responses given.

For peer review only - http://bmjopen.bmj.com/site/about/guidelines.xhtml

Interviews will be recorded, and RAP (Rapid Appraisal) sheets will be completed electronically by the researcher undertaking the interview, and one other member of the research team.

Qualitative approach: focus groups

The aim will be to run two focus groups in each children's hospital, each lasting approx. 40-60 minutes. One focus group will be with 4-8 nurses and allied health professionals (e.g. physiotherapists, occupational therapists) and one with 4-8 members of professional, administrative and support staff (e.g. hospital teachers, play leaders, secretaries, receptionists, porters, security, cleaners). Again, our aim is to work across all ten locations, with a view to accessing some participants from each category in a minimum of six locations. This means that we will recruit a minimum of 24 and a maximum of 80 nurses and allied health professionals, and a minimum of 24 and a maximum of 80 professional, administrative and support staff to join focus groups and address the research questions.

The focus groups will be conducted face to face, unless prevented by pandemic infection prevention and control protocols. In this instance, focus groups will be conducted online via Microsoft Teams. All participants taking part in the focus groups must be able and willing to give informed consent.

A draft topic guide for the focus groups has been created to guide discussion and maximise relevance and appropriateness. Focus groups will be recorded, and RAP (Rapid Appraisal) sheets will be completed electronically by the researcher undertaking the interview, and one other member of the research team.

Data Analysis

Rapid assessment procedures aim to produce a rapid but rigorous qualitative analysis (Vindrola-Padros et al, 2020). It is predominantly team based and collaborative in nature, and conducted as an iterative process alongside data collection (Vindrola-Padros, 2021). RAP sheets will be used as the basis for debriefing sessions. Depending on team capacity, one or two RAP sheets will be completed for each interview or focus group (the aim will be two for each where possible). These standardised and structured data collection forms will guide our reflexive analysis process. In group debriefing sessions, we will focus on identifying key themes from these RAP sheets, creating brief summaries of data in tables that will then be used iteratively for cross-case comparison.

Using multiple population groups (senior leaders, doctors, nurses, allied health professionals and professional/ support staff), multiple data sources (published and grey literature, interviews and focus groups) and multiple experienced researchers means that we will be able to triangulate insights (Morse, 2015). Our aim is to produce a targeted analysis that leads to actionable recommendations and enables cross-case comparison between sites to identify good practices (Silverman, 2015).

5 STUDY SETTING

This is a multi-centre research project. The sites are:

- Sheffield Children's NHS Foundation Trust
- Alder Hey Children's Hospital
- Great Ormond Street Hospital
- Birmingham Women's and Children's NHS Foundation Trust
- Royal Manchester Children's Hospital
- Leeds Children's Hospital
- Bristol Royal Hospital for Children
- Evelina London Children's Hospital
- Southampton Children's Hospital
- The Oxford Children's Hospital

For peer review only - http://bmjopen.bmj.com/site/about/guidelines.xhtml

The ten children's hospitals in the Children's Hospital Alliance (England) have been collaborating through the National Paediatric Accelerator Programme to support elective recovery, share best-practice and reduce health inequalities. As a group, these organisations are seeking to develop understanding as to how acute trusts working together, can improve access to quality paediatric healthcare.

6 SAMPLE AND RECRUITMENT

Eligibility Criteria

All participants will have a current role in one of the ten children's hospitals in the Children's Hospital Alliance (England).

Inclusion criteria for the interviews:

1) A member of the senior leadership team (e.g. executive/ non-executive director plus any other relevant senior leader depending on the structure of the organisation) or a doctor with an interest in health inequalities.

2) Over 18 years.

3) Able and willing to give informed consent at the time of the interview.

Inclusion criteria for the focus groups:

1) Nursing and allied health professional staff (e.g. physiotherapists, occupational therapists), professional, administrative and support staff (e.g. hospital teachers, play leaders, secretaries, receptionists, porters, security, cleaners).

2) Over 18 years.

3) Able and willing to give informed consent at the time of the interview.

Exclusion criteria

1) Age <18 years.

2) Does not have a current role as listed above in one of the ten children's hospitals.

4) Unable or unwilling to give informed consent.

Sampling technique

We will work across ten children's hospitals. We aim to recruit participants as described from each site, as per the funder's aim, but are aware that this may not be possible within the available time. Our aim is to work across all ten locations, with a view to accessing some participants from each category in a minimum of six locations. In each organisation, we will sample:

1. 1-3 members of the senior leadership team (one executive and one non-executive director plus any other relevant senior leader depending on the structure of the organisation). All participants will be over 18 years old and recruited based on their professional role. Because of their professional roles, all participants will be English speaking. It is not proposed to exclude on any further criteria. We will recruit a minimum of six and a maximum of 30 people.

2. 1-3 doctors with an interest in reducing health inequalities. All participants will be over 18 years old and recruited based on their professional role. Because of their professional roles, all participants will be English speaking. It is not proposed to exclude on any further criteria. We will recruit a minimum of six and a maximum of 30 people.

For peer review only - http://bmjopen.bmj.com/site/about/guidelines.xhtml

3. 4-8 nurses and allied health professionals (e.g. physiotherapists, occupational therapists). These potential participants will be recruited via their organisations. All participants will be over 18 years old and recruited based on their professional role. Because of their professional roles, all participants will be English speaking. It is not proposed to exclude on any further criteria. We will recruit a minimum of 24 and a maximum of 80 people.

4. 4-8 members of professional, administrative and support staff (e.g. hospital teachers, play leaders, secretaries, receptionists, porters, security, cleaners). These potential participants will be recruited via their organisations. All participants will be over 18 years old and recruited based on their professional role. Because of their professional roles, all participants will be English speaking. It is not proposed to exclude on any further criteria. We will recruit a minimum of 24 and a maximum of 80 people.

For peer review only

Recruitment

Senior leadership team: senior leaders will be identified via our gatekeeper in the National Paediatric Accelerator, who will provide organisational email addresses to the PI. Potential participants will then be provided with a detailed participant information sheet about the study and a copy of the consent form via email, and then asked to participate by Prof Isba as PI, on behalf of the research team. It will be made clear to potential participants that participation is voluntary.

Doctors: Doctors will be identified via our gatekeeper in the National Paediatric Accelerator who will consult each organisation to identify relevant individuals and provide organisational email addresses to the research team. Potential participants will then be provided with a detailed participant information sheet about the study and a copy of the consent form via email, and then asked to participate by one of the research team. It will be made clear to potential participants that participation is voluntary.

Nurses, allied health professionals, professional, administrative and support staff: These staff will be recruited via their organisations. Each children's hospital will be provided with information about the study, and asked to circulate a call for volunteers to participate via their preferred channel (e.g. email lists, newsletters). Potential participants will be asked to email the research team. The research team will then provide a detailed participant information sheet about the study and a copy of the consent form to all interested participants.

No payment will be made to participants.

Consent

Formal written consent supported by a participant information sheet will be sought for the recorded interviews and focus groups. This will be received by the researcher conducting the interview or focus group. On initial agreement by the participant to conduct the interview or focus group they will be provided with a copy of the consent form and participant information sheet. Due to the logistics of the study there will be a period of time between initial agreement and the interview or focus group taking place. This will allow enough time to ensure the participant is entirely happy to be involved and has time to ask questions. Given the study populations, no problems with understanding the consent process are anticipated.

6 ETHICAL AND REGULATORY CONSIDERATIONS

Assessment and management of risk

The research team have all undertaken Good Clinical Practice training, and completed Disclosure and Barring Service checks. As experienced researchers in healthcare, they are experienced in managing safeguarding issues. Limits to confidentiality will be outlined in the participant information sheets,

For peer review only - http://bmjopen.bmj.com/site/about/guidelines.xhtml

stating that if the participant or someone else is at significant risk of harm, the researcher will break confidentiality and speak to a member of the National Paediatric Accelerator Programme. If possible, the researcher will inform the participant before they do this.

All participants will be given a numeric reference/ pseudonym. For the focus groups and interviews, we will anonymise the data (using pseudonyms) in RAP sheets and onwards. Any identifying details (names/ details of incidents) will be anonymised in the report/ any future publications.

Information about confidentiality and its limitations will be included in each Participant Information Sheet, and will be explained to each participant before starting the interview/ focus group. We will verbally remind participants not to share personally identifiable data in focus groups, and not to share focus group content outside the session.

The interviews and focus groups do not cover areas that are potentially sensitive, but the research team are experienced in conducting interviews and focus groups on sensitive topics. We do not anticipate risks to participating in the study. Although participants may find participating interesting, there are no direct benefits in taking part. A Lancaster University Health and Safety assessment has been completed to ensure safety of the research team and participants.

Research Ethics Committee (REC) and other Regulatory review & reports

This study does not require NHS REC approval; however it does require HRA approval via the IRAS system and ethical approval from the Lancaster Faculty of Health and Medicine Ethics Committee. This has been confirmed via the HRA queries helpline.

Before the start of the study, a favourable opinion will be sought from the Lancaster FHM REC for the study protocol, informed consent forms and other relevant documents e.g. advertisements.

Substantial amendments that require review by the REC will not be implemented until that review is in place and other mechanisms are in place to implement at site.

All correspondence with the REC will be retained.

If the study is ended prematurely, the Chief Investigator will notify the REC, including the reasons for the premature termination.

Amendments

Amendments will be managed via the FHM REC application for amendment to previously approved research form. Following approval each site will be informed of the amendment via the research and development manager in each trust.

Amendments will be discussed with the funder and in quarterly oversight meetings.

Amendments will be tracked by keeping an electronic trail of applications and responses to applications for amendments in an access controlled OneDrive folder.

The decision to make an amendment will be taken by the CI with input from the research team.

Protocol compliance

Protocol deviations will be reported in writing to the sponsor (via the Faculty of Health and Medicine REC). Protocol violation will lead to an internal review of protocol with the research team. Recurring protocol deviations will pause the study and lead to substantive changes to the protocol and re-review by the Faculty of Health and Medicine REC.

For peer review only - http://bmjopen.bmj.com/site/about/guidelines.xhtml

End of study

The end of the study is defined as the submission of the final report to the funder.

Data protection and participant confidentiality

All investigators and study site staff will comply with the requirements of the General Data Protection Regulations and the Data Protection Act 2018. All use of personal information during the course of the project will be in accordance with the General Data Protection Regulations and the Data Protection Act 2018. We do not expect to collect any sensitive data.

Personal data (names, job roles, email addresses and phone numbers) will be given by participants when they contact the research team to arrange interviews/ focus groups. This information will only be used to arrange interviews and focus groups, and will be stored in a password-protected spreadsheet in an access-controlled OneDrive folder for the duration of the project. This information will be held separately from the data set. Personal data will only be accessible to the research team. Consent forms will be stored electronically in an access-controlled OneDrive folder, until the end of the project. If consent is taken in paper form, these forms will be scanned and uploaded to the access controlled OneDrive folder, as soon as possible after consent is taken, and originals will be securely shredded.

Lancaster University will be the data controller. For further information about how Lancaster University processes personal data for research purposes, please visit: www.lancaster.ac.uk/research/data-protection

Three sources of data will be gathered and analysed:

1. Interview video recordings.

Because interviews will be conducted via Microsoft Teams, these interviews will be video recorded. It is not possible to only audio record in Teams, so we will need to store video, though these will not be used for analysis. Video recordings will be accessed by another member of the research team, who will listen to the interview and complete a RAP sheet (see 3 below), which will then be stored digitally. Video recordings made on Teams are automatically stored on Lancaster University's Microsoft Stream platform. Only the meeting owner (the researcher/ project administrator) has access to the recording, but will grant access to another member of the research team for analysis (see above). All video recordings will be deleted by the meeting owner within six months of the end of the project (June 2023). From June 2022, automatic storage infrastructure will change, and videos will automatically be stored in OneDrive. The same access restrictions and plans for deletion will apply.

2. Focus group audio recordings. If pandemic protocols mean that focus groups have to be conducted remotely, video recording storage/ collection will apply (see 1 above).

Focus group audio recordings will be copied to the access controlled OneDrive folder and deleted from the capture device on the day of creation or as soon as possible afterwards. This prevents accidental data disclosure and guarantees that the data are backed-up. Digital recordings will be identified with a participant number only. Audio recordings of focus groups will be destroyed within six months of the end of the project. Audio and video recordings will not be transcribed in full, but anonymised quotations identified with a participant number may be transcribed into RAP sheets.

3. RAP sheets, completed by the research team.

RAP (Rapid Appraisal) sheets will be completed electronically in Microsoft Word and stored on the access-controlled OneDrive folder. They will constitute the main source of data from human participants to be retained and will be stored for ten years as per university policy. RAP and RREAL methodologies emerge from a qualitative research tradition that recognises the need for rapid analysis of data to produce meaningful recommendations for practice in a timely manner (Vindrola-Padros et al, 2020). RAP sheets will be used for team debriefs and analysis.

For peer review only - http://bmjopen.bmj.com/site/about/guidelines.xhtml

The researchers may take written notes as an aide-memoire of these focus groups, and of their general impressions and reflections on any informal encounters while onsite, but these notes will not constitute a formal source of data or contain identifiable data. These notes will be destroyed (shredded) following the completion of RAP sheets for the session.

Participants will be informed that they may withdraw from the study at any time before or during the interview/ focus group, and up to two weeks following their interview/ focus group participation without giving any reason. If the request to withdraw comes after data have been anonymised, and incorporated into themes, it might not be possible for it to be withdrawn, though every attempt will be made to extract the data, up to the point of delivering the report to the funder.

Lancaster University will act as the data custodian. Day-to-day data management will be undertaken by the PI. Written/ digital data will be stored in a Onedrive folder with access limited to the project team for ten years after the end of the project. After this, it will be deleted and responsibility for data management will lie with Professor Rachel Isba as principal investigator.

Indemnity

Lancaster University legal liability cover will apply:

- to meet the potential legal liability of the sponsor(s) for harm to participants arising from the management of the research
- to meet the potential legal liability of the sponsor(s) or employer(s) for harm to participants arising from the design of the research
- to meet the potential legal liability of investigators/collaborators arising from harm to participants in the conduct of the research

No arrangement for payment of compensation in the event of harm to the research participants where no legal liability arises has been made.

Personal research devises (laptop, mobile telephone) are insured by the researcher personally.

7 DISSEMINATION POLICY

As a mandatory clause in the consultancy contract, ownership of all intellectual property and data rights for the project will rest with Sheffield Children's Hospital (SCH) (on behalf of all the Trusts participating in the project) and we will not publish any aspect of the research without permission from Sheffield Children's Hospital. SCH agree that permission will not be unreasonably withheld, but data not be disseminated or shared for reuse.

We will produce a report and presentation of findings for the funder. We will discuss possible wider dissemination of selected elements of work via publishing in an academic journal or presenting at a conference with the funder after delivering this report.

8 REFERENCES

Arksey, H., & O'Malley, L. (2005). Scoping studies: towards a methodological framework. International Journal of Social Research Methodology, 8(1), 19–32. https://doi.org/10.1080/1364557032000119616

For peer review only - http://bmjopen.bmj.com/site/about/guidelines.xhtml

Daudt, H. M., van Mossel, C., & Scott, S. J. (2013). Enhancing the scoping study methodology: a large, inter-professional team's experience with Arksey and O'Malley's framework. BMC Medical Research Methodology, 13(1), 48. https://doi.org/10.1186/1471-2288-13-48

Edge Health (2021) "Analysis of inequalities and "was not broughts" for outpatients appointments". (Analysis prepared for the Paediatric Accelerator inequalities group). Unpublished

Khalil, H., Peters, M., Godfrey, C. M., McInerney, P., Soares, C. B., & Parker, D. (2016). An Evidence-Based Approach to Scoping Reviews. Worldviews on Evidence-Based Nursing, 13(2), 118–123. https://doi.org/10.1111/wvn.12144

Kvale, S., & Brinkmann, S. (2014). InterViews. SAGE Publications.

Marmot, M., Allen, J., Goldblatt, P., Herd, E., Morrison, J. (2020). Build Back Fairer: The COVID-19 Marmot Review. The Pandemic, Socioeconomic and Health Inequalities in England. London: Institute of Health Equity

Morse, J. M. (2015). Critical Analysis of Strategies for Determining Rigor in Qualitative Inquiry. Qualitative Health Research, 25(9), 1212–1222. https://doi.org/10.1177/1049732315588501

Peters, M. D., Godfrey, C., McInerney, P., Soares, C. B., Khalil, H., & Parker, D. (2017). Chapter 11: Scoping Reviews. In M. Z. Aromataris E (Ed.), Joanna Briggs Institute Reviewer's Manual. The Joanna Briggs Institute. Retrieved from https://reviewersmanual.joannabriggs.org/

Silverman, D. (2015). Interpreting Qualitative Data. SAGE.

Vindrola-Padros, C., Chisnall, G., Cooper, S., Dowrick, A., Djellouli, N., Symmons, S.M., Martin, S., Singleton, G., Vanderslott, S., Vera, N. and Johnson, G.A., (2020). Carrying out rapid qualitative research during a pandemic: emerging lessons from COVID-19. Qualitative health research, 30(14), pp.2192-2204

Vindrola-Padros, C., (2021) Doing Rapid Qualitative Research. SAGE.

For peer review only - http://bmjopen.bmj.com/site/about/guidelines.xhtml

9. APPENDICES

Appendix 1- Required documentation

- CV R Isba
- CV J Lunn
- CV L Brewster
- CV L Brennan
- Participant Information Sheet interviews
- Participant Information Sheet focus groups
- Consent Form interviews
- Consent Form focus groups
- Email interview invitation
- Focus groups promotional material
- Interview Guide
- Focus group guide
- RAP sheet

Appendix 2 – Schedule of Procedures

- HRA Schedule of Events
- HRA Organisation Information Document
- HRA Is My Study Research Result
- HRA Does My Study Need NHS REC Approval Result

Appendix 3 – Amendment History

No amendments – first submission

Amendment No.	Protocol version no.	Date issued	Author(s) of changes	Details of changes made

For peer review only

For peer review only - http://bmjopen.bmj.com/site/about/guidelines.xhtml

Children's Hospitals Inequalities Research Project

Lancaster University

Interview Schedule

Senior leaders, NEDs and doctors

Welcome and introduction - Seek consent to continue, reminder of audio-recording of interview (/video recording if on Teams), re-cap of project and plan for interview.

The interviews will explore your views on the Trust's policies and practices around health inequalities. We are interested in your perceptions, on the basis of your knowledge and experience. If you do not feel you are able to comment on any area please say so. Do you have any questions before we start?

For peer review only

1. What does the term 'the socio-economic inequalities in paediatric care' mean to you?
    - Prompt: an example of a socio-economic inequality in health is that children from the most deprived or poorest areas have a shorter life expectancy than people in the wealthiest areas.

2. In your experience, does deprivation have an impact on children's health?
    - Prompt: by deprivation we mean things like people living in areas with lower levels of income, employment, higher crime, less education

3. In your experience, does deprivation have an impact on whether or not children access care?
    - Prompt: What stops people from deprived areas from bringing their children to hospital for appointments and care?

4. Do you see health inequalities in the patients using this hospital?
    - What are these? How do they affect care (if not outlined above)

5. What do you see as your role in regard to health inequalities?

6. <u>Doctors only:</u> Are there any processes in place to help you, if you see health inequalities?
    - Prompt: have you received training, are there systems for flagging concerns?
    - Are there any barriers to this? Prompt: E.g. costs of care for patients, timings/ locations of appointments.

7. Does the Trust have a shared organisational view on its role in health inequalities?
    - Prompt: Are you aware of a statement by the board?

8. Are there any organisational policies in place relating to socio-economic barriers to accessing healthcare?

9. Do you think that COVID has affected socioeconomic inequalities?
    - (if yes) What is the Trust doing to address the impacts of COVID?
    - What impact, if any, has COVID had on access and quality at the Trust?

For peer review only - http://bmjopen.bmj.com/site/about/guidelines.xhtml

10. What does the Trust do to reduce health inequalities?
    - What could they do better/ more of?
    - Are there any barriers to this?

11. What would ‘best practice’ in addressing the socio-economic barriers to paediatric care look like?
    - Prompt: Have you seen evidence from elsewhere in the UK or internationally of what works well?

12. <u>Senior leaders only:</u> As an employer, does the Trust have a role in considering in wider society and socio-economic inequalities?

13. <u>Senior leaders only:</u> Any specific questions about hospital policies that have been reviewed for clarification.

Anything not covered?

Is there anything that we haven’t covered in the interview that you think we should know or think about?

Closing and thanks - Thank for their time and contribution.

For peer review only

Children's Hospitals Inequalities Research Project

Lancaster University

Focus Group Schedule

Nurses, Allied Health Professionals

Professional, administrative and support staff

Welcome and introduction - Seek consent to continue, reminder of audio-recording of the group, establish ground rules, re-cap of project and plan for focus group

The focus group will explore your views on the Trust's policies and practices around health inequalities. We are interested in your perceptions, on the basis of your knowledge and experience. Please try not to use any details which might lead someone in the group to be able to identify another staff member/ patient (e.g. their name). If you do not feel you are able to comment on any area please say so. Does anyone have any questions before we start?

1. In your experience, does deprivation have an impact on children's health?
   - Prompt: by deprivation we mean things like people living in areas with lower levels of income, employment, higher crime, less education

2. In your experience, does deprivation have an impact on whether or not children access care?
   - Prompt: What stops people from deprived areas from bringing their children to hospital for appointments and care?

3. Do you see health inequalities in the patients using this hospital?
   - What are these? How do they affect care (if not outlined above)

4. What do you see as your role in regard to health inequalities?

5. Do you know if the Trust has a policy, or a view, on its role in health inequalities?
   - (If yes) What is it? Can you say more?
   - (If no) Why do you think it does not have a policy/ view?

6. Are there any processes in place to help you, if you see health inequalities?
   - Prompt: have you received training, are there systems for flagging concerns?
   - Are there any barriers to this? Prompt: E.g. costs of care for patients, timings/ locations of appointments.

7. What does the Trust do to reduce health inequalities?
   - What could they do better/ more of?

Anything not covered?

Is there anything that we haven't covered in the focus group that you think we should know or think about?

Closing and thanks

Thank for their time and contribution.

For peer review only - http://bmjopen.bmj.com/site/about/guidelines.xhtml

Children's Hospitals Inequalities Research Project

Lancaster University

Study: Children's Hospitals Inequalities Research Project (CHIRP).

Researcher name	
Study location (name of hospital)	
Interview/ focus group (delete as applicable)	If focus group: number of participants =
Participant group (leadership, clinician, other)	
Examples of socio-economic inequalities	
Deprivation: impact on children's health	
Deprivation: impact on access to care	
Deprivation: visibility in their context (hospital)	
Perceived/ outlined role in health inequalities	
Awareness of policies/ policies and organisational views	
Processes in place for support	

For peer review only

Impact of Covid	
What the Trust currently does	
What the Trust might do more of/ better	
What stops the Trust doing more/ better	
Examples of best practice	
Clarification points (policies; SL only)	
Miscellaneous	
Other comments	

For peer review only

Standards for Reporting Qualitative Research (SRQR)*

http://www.equator-network.org/reporting-guidelines/srqr/

Title and abstract

	Page/line no(s).
**Title** - Concise description of the nature and topic of the study Identifying the study as qualitative or indicating the approach (e.g., ethnography, grounded theory) or data collection methods (e.g., interview, focus group) is recommended	1/ 1-3
**Abstract** - Summary of key elements of the study using the abstract format of the intended publication; typically includes background, purpose, methods, results, and conclusions	1/ 5-28

Introduction

**Problem formulation** - Description and significance of the problem/phenomenon studied; review of relevant theory and empirical work; problem statement	2/ 41-84
**Purpose or research question** - Purpose of the study and specific objectives or questions	3/ 85-86

Methods

**Qualitative approach and research paradigm** - Qualitative approach (e.g., ethnography, grounded theory, case study, phenomenology, narrative research) and guiding theory if appropriate; identifying the research paradigm (e.g., postpositivist, constructivist/ interpretivist) is also recommended; rationale**	3/89-100
**Researcher characteristics and reflexivity** - Researchers' characteristics that may influence the research, including personal attributes, qualifications/experience, relationship with participants, assumptions, and/or presuppositions; potential or actual interaction between researchers' characteristics and the research questions, approach, methods, results, and/or transferability	4/ 122-124
**Context** - Setting/site and salient contextual factors; rationale**	3-4/102-120
**Sampling strategy** - How and why research participants, documents, or events were selected; criteria for deciding when no further sampling was necessary (e.g., sampling saturation); rationale**	3-4/102-120 and 4154-158
**Ethical issues pertaining to human subjects** - Documentation of approval by an appropriate ethics review board and participant consent, or explanation for lack thereof; other confidentiality and data security issues	4/143-147
**Data collection methods** - Types of data collected; details of data collection procedures including (as appropriate) start and stop dates of data collection and analysis, iterative process, triangulation of sources/methods, and modification of procedures in response to evolving study findings; rationale**	4/121 - 142

For peer review only - http://bmjopen.bmj.com/site/about/guidelines.xhtml

**Data collection instruments and technologies** - Description of instruments (e.g., interview guides, questionnaires) and devices (e.g., audio recorders) used for data collection; if/how the instrument(s) changed over the course of the study	4/126 and 4/ 136 plus supplementary materials
**Units of study** - Number and relevant characteristics of participants, documents, or events included in the study; level of participation (could be reported in results)	3/108-112
**Data processing** - Methods for processing data prior to and during analysis, including transcription, data entry, data management and security, verification of data integrity, data coding, and anonymization/de-identification of excerpts	4/159-171
**Data analysis** - Process by which inferences, themes, etc., were identified and developed, including the researchers involved in data analysis; usually references a specific paradigm or approach; rationale**	4-5/149-177
**Techniques to enhance trustworthiness** - Techniques to enhance trustworthiness and credibility of data analysis (e.g., member checking, audit trail, triangulation); rationale**	5/172-177

Results/findings

**Synthesis and interpretation** - Main findings (e.g., interpretations, inferences, and themes); might include development of a theory or model, or integration with prior research or theory	5/178-346
**Links to empirical data** - Evidence (e.g., quotes, field notes, text excerpts, photographs) to substantiate analytic findings	5/178-346

Discussion

**Integration with prior work, implications, transferability, and contribution(s) to the field -** Short summary of main findings; explanation of how findings and conclusions connect to, support, elaborate on, or challenge conclusions of earlier scholarship; discussion of scope of application/generalizability; identification of unique contribution(s) to scholarship in a discipline or field	6/347-357 and 6/ 372-385
**Limitations** - Trustworthiness and limitations of findings	6/65-371

Other

**Conflicts of interest** - Potential sources of influence or perceived influence on study conduct and conclusions; how these were managed	Title page
**Funding** - Sources of funding and other support; role of funders in data collection, interpretation, and reporting	Title page

*The authors created the SRQR by searching the literature to identify guidelines, reporting standards, and critical appraisal criteria for qualitative research; reviewing the reference lists of retrieved sources; and contacting experts to gain feedback. The SRQR aims to improve the transparency of all aspects of qualitative research by providing clear standards for reporting qualitative research.

For peer review only

For peer review only - http://bmjopen.bmj.com/site/about/guidelines.xhtml

**The rationale should briefly discuss the justification for choosing that theory, approach, method, or technique rather than other options available, the assumptions and limitations implicit in those choices, and how those choices influence study conclusions and transferability. As appropriate, the rationale for several items might be discussed together.

Reference:

O'Brien BC, Harris IB, Beckman TJ, Reed DA, Cook DA. **Standards for reporting qualitative research: a synthesis of recommendations.** *Academic Medicine*, Vol. 89, No. 9 / Sept 2014 DOI: 10.1097/ACM.0000000000000388

For peer review only

For peer review only - http://bmjopen.bmj.com/site/about/guidelines.xhtml

BMJ Open

Understanding responsibility for health inequalities in children's hospitals in England: a qualitative study with hospital staff

Journal:	*BMJ Open*
Manuscript ID	bmjopen-2023-081056.R1
Article Type:	Original research
Date Submitted by the Author:	12-Dec-2023
Complete List of Authors:	Brewster, Liz; Lancaster University, Lancaster Medical School<br>Brennan, Louise; Lancaster University Faculty of Health and Medicine<br>Hindocha, Avni; Manchester University NHS Foundation Trust, Royal Manchester Children's Hospital; Lancaster University, Lancaster Medical School<br>Lunn, Judith; Lancaster University<br>Isba, Rachel; Lancaster University Faculty of Health and Medicine
**Primary Subject Heading**:	Paediatrics
Secondary Subject Heading:	Health policy, Public health
Keywords:	Hospitals, Health Equity, PAEDIATRICS, PUBLIC HEALTH, QUALITATIVE RESEARCH

SCHOLARONE™
Manuscripts

I, the Submitting Author has the right to grant and does grant on behalf of all authors of the Work (as defined in the below author licence), an exclusive licence and/or a non-exclusive licence for contributions from authors who are: i) UK Crown employees; ii) where BMJ has agreed a CC-BY licence shall apply, and/or iii) in accordance with the terms applicable for US Federal Government officers or employees acting as part of their official duties; on a worldwide, perpetual, irrevocable, royalty-free basis to BMJ Publishing Group Ltd ("BMJ") its licensees and where the relevant Journal is co-owned by BMJ to the co-owners of the Journal, to publish the Work in this journal and any other BMJ products and to exploit all rights, as set out in our licence.

The Submitting Author accepts and understands that any supply made under these terms is made by BMJ to the Submitting Author unless you are acting as an employee on behalf of your employer or a postgraduate student of an affiliated institution which is paying any applicable article publishing charge ("APC") for Open Access articles. Where the Submitting Author wishes to make the Work available on an Open Access basis (and intends to pay the relevant APC), the terms of reuse of such Open Access shall be governed by a Creative Commons licence – details of these licences and which Creative Commons licence will apply to this Work are set out in our licence referred to above.

Other than as permitted in any relevant BMJ Author's Self Archiving Policies, I confirm this Work has not been accepted for publication elsewhere, is not being considered for publication elsewhere and does not duplicate material already published. I confirm all authors consent to publication of this Work and authorise the granting of this licence.

For peer review only

UNDERSTANDING RESPONSIBILITY FOR HEALTH INEQUALITIES IN CHILDREN'S HOSPITALS IN ENGLAND: A QUALITATIVE STUDY WITH HOSPITAL STAFF

ABSTRACT

Objectives: To understand how staff in children's hospitals view their responsibility to reduce health inequalities for the children and young people who access their services.

Design: Exploratory qualitative study.

Setting: Nine children's hospitals in England.

Participants: 217 members of staff contributed via interviews and focus groups conducted January-June 2023. Staff were represented at all levels of the organisations, and all staff who volunteered to contribute were included in the study.

Analysis: RREAL methodology for rapid assessment procedures.

Results: All of the children's hospitals were taking some action to reduce health inequalities. Two key themes were identified. First, it was clear that reducing health inequalities was seen as something that was of vital import and should be part of staff's day-to-day activity, framed as 'everyone's business.' Many staff felt there was an obligation to intervene to ensure that children and young people receiving hospital treatment were not further disadvantaged by, e.g. food poverty. Second, however, the deeply entrenched and intersectional nature of health inequalities sometimes meant that these inequalities were complex to tackle, with no clear impetus to specific actions, and could be framed as 'no-one's responsibility'. Within a complex health and social care system, there were many potential actors who could take responsibility for reducing health inequalities, and staff often questioned whether it was the role of a children's hospital to *lead* these initiatives.

Conclusions: Broadly speaking, senior leaders were clear about their organisational role in reducing health inequalities where they impacted on access and quality of care, but there was some uncertainty about the perceived boundaries of responsibility. This led to fragility in the sustainability of activity, and a lack of joined-up intervention. Most hospitals were forging ahead with activity, considering that it was more important to work to overcome health inequalities rather than debate whose job it was.

STRENGTHS AND WEAKNESSES:

- This is the first study to investigate staff's views on health inequalities in children's hospitals in England.
- The sample size of over 200 participants created a substantial dataset for qualitative analysis, which indicates that the findings are robust and transferrable.
- Participants were self-selecting and so may have volunteered to participate because they had a particular interest in sharing views on health inequalities.

For peer review only

UNDERSTANDING RESPONSIBILITY FOR HEALTH INEQUALITIES IN CHILDREN'S HOSPITALS IN ENGLAND: A QUALITATIVE STUDY WITH HOSPITAL STAFF

INTRODUCTION

Definitions of health inequalities vary, but there is broad agreement that these inequalities are unfair and avoidable (1). These inequalities are also systemic or structural rather than purely individual (2,3). Health inequalities are experienced on a social gradient, with those who are more socioeconomically disadvantaged facing greater health challenges, in experiences of illness and access to healthcare (4). In the UK, Michael Marmot and the Institute of Health Equity have been key contributors to driving forward an agenda that recognises the impact of health inequalities on children and young people (5,6). In 2010, the publication of the Marmot Review 'Fair Society, Healthy Lives' (7) set out a framework for action that emphasised how these inequalities accumulate across the lifespan. The recommendations prioritised children as a population group requiring early and meaningful intervention to prevent health inequalities becoming entrenched. This review was updated in 2020, presenting evidence that the gap in health status and outcomes between affluent and socio-economically deprived areas had increased rather than decreased (8).

Children living in areas of high deprivation are more likely to have worse health outcomes (9) and long-term conditions like asthma (10). Access to care also differs, with those from the most deprived areas being less likely to be brought to outpatient appointments (11). Health inequalities therefore represent a tangible issue that needs to be addressed internationally, and have a direct impact on the effectiveness of health service provision.

In the UK, healthcare is publicly-funded and provided free at the point of use, via the National Health Service (NHS). NHS organisations have a statutory duty with regards to specific equality domains defined by the Equality Act 2010 (12), which outlined protected characteristics (e.g. age, sex, race) but there had not been a specific duty on NHS organisations to intervene with regard to health inequalities caused by socio-economic deprivation until the Health and Social Care Act 2012 (13), which introduced the first legal duties relating to health inequalities. Health inequalities are also part of several subsequent national strategies, e.g. the NHS Long Term Plan (14) and the NHS People Plan (15). These top-level strategies focus on a legal duty to reduce inequalities in access, and outcomes for all without specific guidance on how this may be implemented.

Both socio-economic and health inequalities are thought to have increased since the SARS-CoV-2/COVID-19 pandemic, impacting health outcomes, with longer waiting times for elective care. Greater attention has been given to reducing inequalities because of this increase, and the impact on children and young people has been identified as a particular focus for intervention, via initiatives such as NHS England's 'Core20PLUS5' approach (16). This recognises that inequalities are intersectional, focusing on the most deprived 20% of the population, prioritising inclusion, e.g. of people from ethnic minority communities, and identifying five clinical areas of focus: asthma, diabetes, epilepsy care for those with a learning disability, oral health, and mental health.

Within the NHS, children's hospitals provide specialist paediatric care to children and young people, typically up to the age of 16. This provision can be as a stand-alone hospital for children, or as part of a wider organisational structure (NHS trust). There is great diversity amongst service provision (including amongst the sites in this study) because of these organisational structures, but in the main children's hospitals offer services (e.g. emergency and acute general medicine, community and outpatient care) to their local population, and

For peer review only

then specialist services to patients with more complex medical needs (e.g. paediatric cardiac surgery) across a regional or national catchment. Children and young people and their caregivers may therefore travel a great distance to access these more specialist services.

This study aimed to understand how staff in children's hospitals in England view their responsibility to reduce health inequalities for the children and young people they serve.

METHODS

Qualitative data were collected via interviews and focus groups, working across nine children's hospitals in England, as part of a wider research project (supplementary file 1). The qualitative approach was selected to explore participants' understanding of current policy and practice around socio-economic inequalities and their impact on healthcare, rather than hypothesising about, or predicting, responses (17).

Rapid Research Evaluation and Appraisal (RREAL) methodologies (18) were used to collect and analyse data; these methodologies emerge from a qualitative research tradition that recognises the need for rapid data analysis to produce meaningful recommendations for practice in a timely manner. The full process had four stages, in which data were collected, recorded, synthesised, and analysed to produce key recommendations. Using multiple population groups (senior leaders, doctors, nurses, allied health professionals, and professional/ support staff), multiple data sources (interviews and focus groups), and multiple experienced researchers, allowed us to ensure rigour by triangulating insights (19).

Recruitment and participants

Hospitals sampled are all members of the Children's Hospital Alliance, which commissioned a broader research project to exploring the roles that their member organisations can play in reducing socio-economic barriers to paediatric care, and in understanding and developing best practice to do this. At time of the study, the Children's Hospital Alliance had ten members; it now has eleven. As a national network of specialist NHS trusts focused on paediatric care in the UK, it initially focused on support for pandemic recovery for children and young people. One of the original ten member organisations was unable to participate in this study due to capacity. The hospitals varied in geographic location, percentage of local population living in the most 20% of deprived communities, organisational size and structure, and services offered to the paediatric population.

Recruitment was organised by the nine participating organisations who circulated a call to volunteer to participate and organised access to rooms/ teams on site. In total, 217 members of staff were included across the sites (table 1).

For peer review only

Organisation	Interviews	Focus Groups/ conversations	Total number of participants per site
Site A	9	4 groups, 19 participants	28
Site B	4	4 groups, 29 participants	33
Site C	7	2 groups, 8 participants	15
Site D	10	4 groups, 12 participants	22
Site E	4	2 groups, 15 participants	19
Site F	11	15 ‘walk arounds’; 42 participants	53
Site G	6	1 group, 5 participants	11
Site H	5	2 groups, 15 participants	20
Site I	5	2 groups, 11 participants	16
**TOTAL**	**61**	**39** groups/conversations	**217**

Table 1: Staff participants across nine NHS organisations

Staff were represented at all levels from senior executives to housekeeping staff. Focus groups were composed of staff from similar categories to ensure that those present felt more comfortable to contribute. To maintain anonymity when presenting data we refer to broad three categories of staff:

- Leadership (n=47) - executive and non-executive directors, anyone involved in the management of initiatives concerned with health inequalities;
- Clinical (n=85) - doctors, nurses, and allied health professionals (e.g. physiotherapists, occupational therapists);
- Professional and support staff (n=85) – e.g. receptionists, porters, security, housekeeping.

Data collection

The research team consisted of two academics with expertise in applied qualitative health research, and three research-active public health specialists. We conducted semi-structured interviews with senior leaders and doctors with an interest in health inequalities across nine organisations. Interviews lasted between 30 and 60 minutes. A structured topic guide (supplementary file 2) was used to provide an initial focus and allow for comparability between respondents, while also allowing for flexibility and individuality in the responses given. Interviews were recorded via Microsoft Teams. In total, 61 interviews were completed, and number of interviews per organisation ranged from 4-11.

We conducted focus groups and had informal conversations with clinical, professional and support staff. The majority of focus groups were audio recorded, but for informal conversations, brief written notes with no identifiable details were made and then team members audio-recorded a debrief session to provide an overview of the conversations. One or two focus groups were conducted per organisation, lasting around 60 minutes per group. Number of participants ranged from 3-10 members of staff. A structured topic guide (supplementary file 3) provided focus for discussion. One site advised that informal ‘walk around’ conversations were more appropriate to engage staff; these discussions were also structured using the same topic guide. Written informed consent was taken for participation in interviews and focus groups, and verbal consent was taken for ‘walk around’ discussions. All nine organisations were visited for one or two days, meaning additional debrief

For peer review only

recordings were often comprehensive and detailed. In total, 39 focus groups and informal discussions were completed. Data were collected between January and June 2023.

Patient and Public Involvement

As part of our wider ongoing programme of work, we have engaged with children and young people and their families to understand barriers to engagement with research around health inequalities (20). This engagement work was conducted concurrently with the study reported here.

Ethical approvals

Ethical approval was granted by FHM Research Ethics Committee Lancaster University on 16 June 2022 (ref: FHM-2022-0844-RECR-3). Health Research Authority approval was granted on 24 August 2022 (ref: IRAS315113 and 22/HRA/3123) and capacity and capability to participate was confirmed by each organisation

Data analysis

Data analysis was conducted in several stages, informed by the RREAL methodology for rapid assessment procedures (RAP) (21).The RAP process is considered an appropriate method to conduct rigorous and robust qualitative data analysis in a short amount of time. It is predominantly team-based and collaborative in nature, and conducted as an iterative process alongside data collection. Data are independently reviewed by multiple team members, before being brought together in a group session for further analysis. RAP is particularly suitable for this type of research, which aims to synthesise diverse perspectives on a defined topic, and is focused on generating useful learning that can be actioned. While it was felt that 'data saturation' (22) was being reached at organisations, it is evident that results are intrinsically linked with the sample of people that we spoke to at each hospital.

Following the first stage of data collection, three further stages (multiple RAP sheet generation, individual consolidation and group confirmation) completed the RREAL process. After each interview, focus group, or debrief, a structured RAP sheet (supplementary file 4) outlining the main themes was completed by the researcher leading the data collection. One or more members of the research team then listened to the recording, and independently completed a RAP sheet. RAP sheet generation occurred simultaneously with data collection, with discussion occurring after data collection had been completed. A minimum of two standardised RAP sheets was completed for each interview/focus group, and used to guide our reflexive analysis process. In total, 171 RAP sheets were generated. Researchers then read and reviewed all the RAP sheets generated, and completed independent informal notes to support synthesis. Two members of the team independently generated further 'site-specific' RAP sheets, providing an overview of key themes at each organisation. This synthesis process focused on considering the specific context of each organisation as well as the broader project research questions.

We then conducted several day-long group debriefing and analysis sessions, in which we focused on identifying key themes from RAP sheets. This process led to the creation of brief data summaries that were then used iteratively for cross-case comparison. The whole team contributed to these sessions, which aimed to establish consensus, while still being sensitive to diversity of views and experiences in different organisations. Each analysis session ended with confirmation and agreement of key themes.

RESULTS

From our qualitative dataset, including the perspectives of 217 staff across nine organisations, two main themes were identified that help to understand how children's hospitals in England view their responsibility to reduce health inequalities for the children

For peer review only

and young people using their services. The first was that staff saw reducing health inequalities as 'everyone's business' and that no child should experience inequalities in care or outcomes because of their socio-economic status. This was particularly visible in terms of the activities that hospitals were undertaking in this space, regardless of national steer and guidance.

However, the second theme – whilst also recognising that health inequalities were deeply entrenched and intersectional – suggested that these inequalities were seen as 'no-one's responsibility' or 'not part of core business' for a children's hospital. Within a complex health and social care system, there were many potential actors who could take responsibility for reducing health inequalities, and staff often questioned whether it was the role of a children's hospital to lead these initiatives rather than treating the presenting illness. This led to some fragility in the sustainability of initiatives.

Nevertheless, all of the children's hospitals were taking some action to reduce health inequalities. Many staff felt there was an obligation to intervene to ensure that children and young people receiving hospital treatment (and their caregivers) were not further disadvantaged by, e.g. food poverty.

Health inequalities as 'everyone's business'

The theme of preventing health inequalities as something that should be 'everyone's business' was observed in discussion of the strategy and day-to-day activities of each organisation. Presentation of this theme considers how 'responsibility' was understood in relation to organisational priorities, and the challenges to taking up this responsibility. We pay particular attention to communication, formal and informal support, and the impact of the pandemic on providing additional evidence of the impact of these inequalities, which built the case for intervention.

All participants were asked about their knowledge of organisational policies and strategies that related to health inequalities. Over half of the nine organisations already had a health inequalities strategy in place, although for larger organisations who also offered adult provision, this was an organisation-wide strategy rather than child-focused. The remaining organisations all discussed making progress towards a strategy. The majority of organisations had a health inequalities steering group and/or forum. More than half had a data dashboard, or made reference to data analysis activity, and this was used to try to identify patients at risk of not attending appointments. This activity was led at an organisational level, rather than linked to national strategy.

Some participants were clear about the organisational strategy around health inequalities, but others were less certain what the overall vision was, or what activities connected to it. In some organisations, there was a named individual with responsibility for health inequalities, but this was not universal, and often, people working on the 'shop floor' were less familiar with the strategic direction. We found that awareness varied in terms of staff knowledge of strategy and policy around health inequalities. While this finding was heavily dependent upon the sample asked, each site had some staff who were unaware of strategy or activity. It became clear that there was a lack of clear communication about work on health inequalities.

> *'It's frustrating as a clinician… I asked my leads and they said they didn't know, there's all these layers of people who didn't know… eventually I found out about a steering group. But it's not affecting my practice what they're doing [about reducing inequalities].* (Clinical, site B)

For peer review only

This lack of communication was one example of how work to reduce health inequalities was fragile. It was often led by keen individuals, and so while health inequalities were seen as something that should be a focus for activities ('everyone's business'), this could be more at the level of a vision that informed activities rather than something with concrete key performance indicators .

Consideration of reduction of health inequalities was clearly visible in interventions led by the organisations. There were several common forms of support across organisations, almost all of these focused on meeting day-to-day basic needs. These included cheap food offers, free food for breastfeeding mothers, free sanitary products, parking/travel reimbursement and hospital transport for specific identified groups, accommodation, and interpreters. While available in the majority of organisations, these basic offers were not universal across all sites and specialties. Other initiatives which occurred less frequently included food vouchers, a universal patient transport offer, and free food on wards for household members.

As well as strategic direction and organisationally-initiated formal support for families, a second, more hidden layer of support was present in many of the organisations. This informal system was largely driven by individuals, often at nursing or domestic support level, and showed how reducing health inequalities permeated into every level of work. Stories of housekeeping staff taking washing from families, buying caregivers coffees and food, and giving leftover food to families were frequent. Similarly, nursing staff discussed ways to order additional food when they noticed someone was in need. In some hospitals, ward managers had access to a budget that funded food vouchers and taxis. This support was partly dependent upon motivated and caring individuals.

> *'I think the people that I speak to, and I speak to a broad range, it's actually part of their core value rather than something that they think would make a good headline. And I think that resonates throughout the entire organization that they would be doing this even if no one was watching them do it.'* (Leadership, site F)

Some staff felt that there needed to be key performance indicators around health inequalities to encourage prioritisation of resource to this area of activity. With no current metrics around health inequalities, it becomes one of the 'nice to haves' rather than an essential.

> *'We don't have standards for the level of support for our families and we should do shouldn't we?'* (Leadership, site D)

> *'We can't allow health inequalities to become a luxury activity for organisations that happen to have the resources.'* (Leadership, site G)

Some staff explained that while health inequalities were not new, the pandemic had highlighted inequalities in outcomes and provided evidence (and therefore understanding) of the health inequalities that passionate members of staff had been trying to explain previously.

> *'I'm sure if you'd have talked about some of this stuff five years ago people wouldn't have understood as much as they do now.'* (Clinical, site A)

It was felt that the pandemic had also shone a focus on inequalities, kickstarting projects such as analysis of data on potential inequalities.

For peer review only

'There's variation in how long certain groups are waiting for care… we've observed in our analysis those with the lowest income wait on average six years longer. Children and young people with a learning disability wait longer to complete their treatment. Both of those factors were there before Covid, but now we have done the analysis and are looking at how we can address it.' (Leadership, site B)

Most of the leadership and clinical staff we interviewed were vocal about the way that health inequalities were their business, and affected their day-to-day provision of healthcare.

'Because if they don't have a good start, they're not gonna have a long and happy life. So they will have poor diet. Doesn't matter what people tell you. It is quite expensive to eat well, so they will have increased diabetes. They will have increased obesity. They'll have all the pitfalls of living in a cold, damp house. So they'll have worse asthma.' (Leadership, site F)

'Making people realise that being curious about why a certain child keeps coming back is just as much my responsibility as a doctor as it is to prescribe the Ventolin Inhaler.' (Clinical, site G)

This idea of 'prevention rather than cure' was epitomised in several examples offered of activity to reduce health inequalities. For example, one clinician discussed how the impacts of having a domestic violence prevention service piloted in their Paediatric Emergency Department might have wide-ranging impacts for children and young people attending the department, but that those effects might be seen in educational attainment in a generation's time rather than being immediately visible.

Health inequalities as 'no-one's responsibility'

In contrast to the above discussion of health inequalities as a priority area when inequalities impacted access and quality of care, this theme highlights how staff varied in their articulation of this priority in practice. Unclear boundaries between the hospital and other services, a lack of a national steer, challenges around wider system connections, concerns about potential cost and a lack of 'embeddedness' to make tackling inequalities part of core work were seen as challenges.

Although, broadly speaking, members of leadership teams were clear about their role in overcoming socio-economic inequalities where they impacted on access and quality of care, there was some uncertainty about the perceived boundaries of responsibility with other institutions (e.g. local government, other healthcare services). Some staff saw that leading the health inequalities agenda was not the role of the hospital, and there was an awareness that other services such as local authorities, public health, schools, community care, and general practice also had a role to play.

'So whose responsibility is it to do this? One answer is everyone's responsibility. Another answer is not ours. And our job is here to see and treat people in front of us. That is one response. And you think well, there are public health teams around. There's lots of stuff. Should we be out there in the city centre running our campaigns about how to brush teeth? Or should we put a dentist in theatre doing the dental extractions? That's the choice.' (Leadership, site F)

For peer review only

Staff at all levels felt that a key barrier to addressing health inequalities action was the lack of national and local priority given to the subject. Senior executives were honest that priority was often given to the national targets such as waiting lists and finances.

> *'It's not the Government's priority. No government is interested in long-term gain. They're interested in waiting lists and how they got better during their jurisdiction.'* (Leadership, site D)

Although some senior staff spoke about working as part of a system, staff members at all levels, and across all organisations, spoke about their frustrations in not feeling part of the system. This was seen as a barrier to advancing the inequalities agenda for children and young people.

> *'It's being holistic as a system that we're not very good at doing. Because we are separate to social services and we're separate to schools, we don't think of 'children's' as a system yet.'* (Leadership, site D)

This was compounded by the inability of IT systems to link up the health and social care system.

> *'No one has time or capacity to make these sort of links happen. It is just not at the top of their agenda to try and get everyone equitable access in one area. You can see a massive discrepancy across the region and actually it does have an impact on death rates in our service.'* (Clinical, site I)

For some organisations, their status within the community as a 'trusted brand' meant that they felt it was within their remit to intervene, regardless of their 'responsibility.' Children's hospitals were seen as well-placed to lead on and communicate upon health inequalities to the wider public.

> *'We've got a trusted brand and … it's well known, it's well understood. And if we went walking up and down the high street with donation boxes, people go oh yeah, I'll give money to the children's hospital.'* (Leadership, Site F)

The picture was more mixed in terms of the views of the broader staff cohort. Some staff saw support for overcoming health inequalities as part of their role, but others felt it was not a priority, or felt less confident in asking questions about deprivation and need. Most health inequalities work was being driven by passionate individuals, making it unsustainable if these people leave. These champions were a positive, in terms of driving action, but their impact was limited by the way in which health inequalities were not part of core business or communicated within their organisations, and often went unnoticed by staff at leadership level. The lack of embeddedness meant that even if the aim was for every member of staff to take responsibility for tackling the health inequalities they encountered, they were not held responsible for delivering this outcome.

A smaller number of staff were concerned about the overall expense of initiatives such as free food and transport, particularly if they were promoted widely. They did not see support for patients as a key role for the organisations, and highlighted the potential for initiatives to be inappropriately accessed by people who did not need the support. They also recognised the impact of increased workload, for example if families were fed alongside patients, and noted that this could be time-consuming. It became clear that for inequalities to become part of core business, a significant shift was required.

DISCUSSION

This paper has presented findings from a large-scale qualitative study aiming to understand how staff in children's hospitals in England view their responsibility to reduce health inequalities for the children and young people who access their services. Key findings were that while reducing health inequalities was broadly seen as important to these organisations, there were a number of barriers to activity, including a lack of agreement about the scope and remit of intervention, and whose priority it was to lead on these interventions. There was little specific national guidance to support decisions around how to manage inequalities in access and outcomes, but organisations could see the consequences of a lack of intervention on a daily basis. Although it was a legal duty, it was one of many competing priorities. Therefore, discussions about when, where, and how to intervene often centred on questions around best use of available resource, and how impact of resource use could not be evidenced or tied to organisational key priorities.

Strengths of this study are that it is, to our knowledge, the first study to investigate staff's views on health inequalities in children's hospitals in England or elsewhere. The sample size of over 200 participants created a substantial dataset for qualitative analysis, which indicates that the findings are robust and transferrable.

Weaknesses include that participants were self-selecting and so may have volunteered to participate because they had a particular interest in sharing views on health inequalities. Rapid qualitative methods with selected transcription and analysis directly from audio recording were used, rather than full transcription of all interviews/ focus groups (21). This may be considered a weakness, but has been accounted for in the rigorous process conducted by the team, where recordings were reviewed multiple times by different researchers and a structured recording form was used to capture data of interest. As all data were recorded, there was opportunity to return to the transcripts as needed throughout the analysis process. Due to the remit of the funding for this research, it was not possible to involve patients or the public in the design, conduct, reporting, or dissemination plans of our research, though our wider research has a strong commitment to this aim. This meant we were also not able to speak to children, young people and families as part of this research, meaning their voices are represented by others within this dataset, which is a weakness of the study. Future research should address these questions around how health inequalities impact on healthcare from the perspective of children and young people in particular.

As identified in a recent scoping review protocol (23), and the review itself (24) there are few academic journal papers that outline activities and interventions conducted by children's hospitals to reduce health inequalities, and more transferrable evidence can be found in the grey literature (defined as sources of literature that have not been formally published). This is partly due to the way that applied intervention and improvement work is often shared in the healthcare sector. Despite the acknowledged importance of reducing health inequalities for children and young people, little research has been conducted in the UK, and this is the first that has sought to understand how staff within NHS organisations perceive their role. This paper therefore makes an important contribution to understanding barriers and challenges around activities to reduce health inequalities and how hospital staff at all levels may contribute to these activities.

A recent review (25) aimed to outline 'the importance of healthcare workers advocating for structural and high-level policy change to address the deep-rooted societal problems that cause child poverty' by proposing a framework to address health inequalities experienced by children. Its broad conclusions support our more detailed account of the entrenched and intersectional nature of health inequalities and how they might be addressed within a

For peer review only - http://bmjopen.bmj.com/site/about/guidelines.xhtml

children's hospital context. In a US context, similar questions are being raised about the potential role of children's hospitals in reducing health inequalities more usually addressed in a community setting (26). A first next step may be to work directly with children and young people in future research to understand more about the challenges they face in relation to these health inequalities as experienced in everyday life.

CONCLUSION AND RECOMMENDATIONS

While there is some clarity for organisations about their role in reducing health inequalities where they impacted on access and quality of care, there remains uncertainty about tackling health inequalities around the wider determinants of health, and the perceived boundaries of responsibility with other organisations within the wider health and social care system. In this study, most hospitals were forging ahead with activity on selected wider determinants, considering that it was more important to work to overcome health inequalities rather than debate whose job it was. While this is laudable, and the contribution of passionate individuals should not be underestimated, it does not position health inequalities clearly as part of children's hospitals' everyday work and so the sustainability of interventions is not guaranteed. The needs of children and young people require much greater attention and stronger, clearer policy at a national level.

Contributorship statement

Isba, Brewster, Brennan and Lunn conceptualised and designed the work. Pearcy was responsible for acquisition of data. Isba, Brewster, Brennan, Broad, Hindocha, Lunn McCreddin and Patel collected the data. Isba, Brewster, Brennan, Hindocha and Lunn analysed the data. Brewster prepared the original draft. Brennan and Isba critically reviewed and edited the work. All authors agreed the final version and are accountable for the accuracy and integrity of the work.

Competing interests

The authors declare that they have no known competing financial interests or personal relationships that could have appeared to influence the work reported in this paper. The views expressed are those of the author(s) and not necessarily those of the Children's Hospital Alliance.

Funding

This work was supported by the National Paediatric Accelerator Programme, now the Children's Hospital Alliance, contracted by Sheffield Children's Hospital, grant number SCH5628.

Data sharing statement

All data relevant to the study are included in the article or uploaded as supplementary information.

CONTRIBUTORS

Eleanor Broad, Dumfries and Galloway Royal Infirmary, UK

Nicola McCreddin, Manchester Royal Infirmary, UK

Pallavi Patel, St Helens and Knowsley Teaching Hospitals NHS Trust, UK

Julie Pearcy, Lancaster Medical School, UK

For peer review only

Acknowledgements

Thank you to everyone at the hospital trusts who helped us arrange interviews and focus groups, and everyone who kindly gave up their time to contribute to our research. Special thanks to Anne Marie Davies, Children's Hospital Alliance and Alder Hey Children's Hospital, Rekel Kerr, Children's Hospital Alliance and Birmingham Children's Hospital and Alexandra Norrish, Children's Hospital Alliance and Sheffield Children's Hospital. Thanks to Tracy Briggs, Heather Catt, Fiona Egboko, Imelda Mayor, Nicola McCreddin, Pallavi Patel, Dora Pestotnik Stres, Esther Primrose and Andrew Rowland for contributions to the wider project.

REFERENCES

1. McCartney G, Popham F, McMaster R, Cumbers A. Defining health and health inequalities. Public Health [Internet]. 2019;172:22–30. Available from: https://www.sciencedirect.com/science/article/pii/S0033350619301076

2. Whitehead M. The concepts and principles of equity and health. Int J Health Serv. 1992;22(3):429–45.

3. Graham H. Social Determinants and Their Unequal Distribution: Clarifying Policy Understandings. Milbank Q [Internet]. 2004 Mar 1;82(1):101–24. Available from: https://doi.org/10.1111/j.0887-378X.2004.00303.x

4. Donkin AJM. Social Gradient. In: The Wiley Blackwell Encyclopedia of Health, Illness, Behavior, and Society [Internet]. 2014. p. 2172–8. Available from: https://doi.org/10.1002/9781118410868.wbehibs530

5. Marmot M. The health gap: The challenge of an unequal world. London: Bloomsbury; 2015.

6. Marmot M. Social determinants of health inequalities. Lancet. 2005;365(9464):1099–104.

7. Marmot M, Allen J, Goldblatt P, Boyce T, McNeish D, Grady M, et al. Fair society, healthy lives: The Marmot Review [Internet]. Vol. 0. London; 2010. Available from: http://dx.doi.org/10.1016/j.puhe.2012.05.014

8. Marmot M, Allen J, Boyce T, Goldblatt P, Morrison J. Health Equity in England : The Marmot Review Ten Years on. London; 2020.

9. Lai ETC, Wickham S, Law C, Whitehead M, Barr B, Taylor-Robinson D. Poverty dynamics and health in late childhood in the UK: evidence from the Millennium Cohort Study. Arch Dis Child [Internet]. 2019 Nov 1;104(11):1049 LP – 1055. Available from: http://adc.bmj.com/content/104/11/1049.abstract

10. Wickham S, Anwar E, Barr B, Law C, Taylor-Robinson D. Poverty and child health in the UK: using evidence for action. Arch Dis Child. 2016 Aug;101(8):759–66.

11. French LR, Turner KM, Morley H, Goldsworthy L, Sharp DJ, Hamilton-Shield J. Characteristics of children who do not attend their hospital appointments, and GPs' response: a mixed methods study in primary and secondary care. Br J Gen Pract. 2017 Jul;67(660):e483–9.

12. HM Government. Equality Act [Internet]. 2010. Available from: https://www.legislation.gov.uk/ukpga/2010/15/contents

13. HM Government. Health and Social Care Act 2012 [Internet]. 2012. Available from: https://www.legislation.gov.uk/ukpga/2012/7/contents/enacted

14. NHS England. The NHS Long Term Plan [Internet]. 2019. Available from: https://www.longtermplan.nhs.uk/publication/nhs-long-term-plan/

For peer review only

15. NHS England. WE ARE THE NHS: People Plan for 2020/2021 - action for us all [Internet]. London; 2020. Available from: https://www.england.nhs.uk/ournhspeople/

16. NHS England. Core20PLUS5 – An approach to reducing health inequalities for children and young people [Internet]. 2023 [cited 2023 Aug 9]. Available from: https://www.england.nhs.uk/about/equality/equality-hub/national-healthcare-inequalities-improvement-programme/core20plus5/core20plus5-cyp/

17. Kvale S, Brinkmann S. InterViews: learning the craft of qualitative research interviewing [2nd edition]. London: Sage; 2009.

18. Vindrola-Padros C, Chisnall G, Cooper S, Dowrick A, Djellouli N, Symmons SM, et al. Carrying Out Rapid Qualitative Research During a Pandemic: Emerging Lessons From COVID-19. Qual Health Res. 2020 Dec;30(14):2192–204.

19. Morse JM. Critical Analysis of Strategies for Determining Rigor in Qualitative Inquiry. Qual Health Res. 2015 Sep;25(9):1212–22.

20. Hindocha A, Brennan L, Brewster L, Lunn J, Isba R. The wider health and wellbeing needs of those accessing paediatric care in England: engaging with the hidden voices of children and young people. Lancet [Internet]. 2023;402:S50. Available from: https://www.sciencedirect.com/science/article/pii/S0140673623021049

21. Vindrola-Padros C. Doing Rapid Qualitative Research. London: Sage Publication; 2021.

22. Charmaz K. Constructing Grounded Theory: a practical guide through qualitative analysis. London: Sage; 2006.

23. Brennan L, Brewster L, Lunn J, Egboko F, Stres DP, Patel P, et al. How do hospitals address health inequalities experienced by children and young people: a grey literature scoping review protocol. BMJ Open [Internet]. 2023 Apr 1;13(4):e071682. Available from: http://bmjopen.bmj.com/content/13/4/e071682.abstract

24. Brennan L, Pestotnik Stres D, Egboko F, Broad E, Brewster L, Lunn J, et al. How do children's hospitals address health inequalities: a grey literature scoping review. BMJ Open. 2023;

25. Lee AR, Kingdon CC, Davie M, Hawcutt D, Sinha IP. Child poverty and health inequalities in the UK: A guide for paediatricians. Arch Dis Child. 2022;108(2):94–101.

26. Franz B, Cronin CE. Are Children's Hospitals Unique in the Community Benefits They Provide? Exploring Decisions to Prioritize Community Health Needs Among U.S. Children's and General Hospitals. Front Public Heal. 2020;8(February):1–10.

For peer review only

FULL/LONG TITLE OF THE STUDY

Children's Hospitals Inequalities Research Project

SHORT STUDY TITLE / ACRONYM

CHIRP

PROTOCOL VERSION NUMBER AND DATE

V1.0 21 April 2022

RESEARCH REFERENCE NUMBERS

**IRAS Number:**	315113
**FUNDERS Number:**	SCH5628 Sheffield Children's Hospitals NHS Foundation Trust (SCH)<br>National Paediatric Accelerator

For peer review only

For peer review only - http://bmjopen.bmj.com/site/about/guidelines.xhtml

STUDY PROTOCOL

Children's Hospitals Inequalities Research Project

1 BACKGROUND

This research will investigate how the ten children's hospitals in the Children's Hospital Alliance (England) view their responsibility to reduce socio-economic health inequalities (e.g. income, location, employment, education, cultural and religious barriers) and how these translate into actions. As part of the National Paediatric Accelerator Programme, we have been asked to look at organisational policies (collect documents) and talk to different groups of staff (in interviews and focus groups) about inequalities experienced by patients, how these affect care (e.g. by being able to attend appointments) and to identify recommendations to develop good practice to ultimately reduce the number of 'Was not Broughts' (the children's equivalent of "Did not attend"), and improve care.

This study will collect qualitative data via interviews and focus groups, which will take place online or at the participants' workplaces. NHS staff participants will be recruited via their organisations. They will include members of the senior leadership team (e.g. executive/ non-executive director plus any other relevant senior leader depending on the structure of the organisation), doctors with an interest in health inequalities, nursing and allied health professional staff (e.g. physiotherapists, occupational therapists), and professional, administrative and support staff (e.g. hospital teachers, play leaders, secretaries, receptionists, porters, security, cleaners).

2 RATIONALE

The ten children's hospitals involved in the study have been collaborating through the National Paediatric Accelerator Programme to support elective recovery, share best practice and reduce health inequalities. As a group, the organisations are seeking to develop understanding as to how acute trusts working together, can improve access to quality paediatric healthcare. The work has included looking at the inequitable impact that the SARS-CoV-2/COVID pandemic has had on the health and wellbeing of children from the poorest backgrounds. In particular, focus has been on the disproportionate impact that deprivation has had on the ability of children from different socioeconomic backgrounds to access healthcare. Children from the most deprived decile in the index of multiple deprivation are more than twice as likely to not be brought to their outpatients' appointments (Edge Health, 2021) than those from the least deprived decile.

It is relatively new for NHS trusts to think about, and seek to take action on, health inequalities relating to deprivation. In considering its role in inequalities, the NHS is most familiar with its statutory role with regards to some specific equality domains. The Equality Act 2010 defines several protected characteristics (i.e. age, sex, race, disability, sexual orientation, gender reassignment, marriage and civil partnerships, pregnancy and maternity, religion or belief discrimination). By comparison with the protected characteristics, there is no specific duty on NHS organisations to intervene with regard to deprivation. However, the Health and Social Care Act 2012 introduced the first legal duties about health inequalities. Health inequalities are also part of several national strategies, such as the NHS Long Term Plan and the NHS People Plan. Initial analysis suggests that that socio-economic factors can have significant impacts on the ability of children and their parents, and recent analysis suggests that the situation has worsened during the pandemic (Marmot et al, 2020).

This research is part of the National Paediatric Accelerator Programme, which includes the remit to reduce the barriers to families preventing them from attending appointments with a view to ultimately reducing the number of 'Was not Broughts', which will support the elective recovery plan for Paediatrics. The research project will explore the roles that these ten trusts can play in reducing socio-economic barriers to paediatric care, and in understanding and developing best practice to do this.

Literature review

As part of the research project, we will conduct a literature-based analysis of the variation in governance approaches and policies around inequalities. This will include documents e.g. policies

For peer review only - http://bmjopen.bmj.com/site/about/guidelines.xhtml

from the ten partner hospitals, published literature including how inequalities are experienced by children and young people in the UK, regional variation, impact of the SARS-CoV-2/COVID19 pandemic, grey literature around inequalities experienced by CYP, including identification of any global exemplars of how to reduce health inequalities.

3 RESEARCH QUESTION/AIM(S)

- How do the ten children's hospitals in the Children's Hospital Alliance (England) view their responsibility to reduce socio-economic health inequalities (e.g. income, location, employment, education, cultural and religious barriers)?
- How does this view of the potential responsibility to reduce socio-economic health inequalities translate into actions in the ten children's hospitals in the Children's Hospital Alliance (England)?
- Is there variation within governance approaches and policies across the ten children's hospitals in the Children's Hospital Alliance (England)?
- Are there areas where there are unmet needs for initiatives to reduce socio-economic health inequalities, either generally or due to changes in service delivery since the pandemic?
- How do the ten children's hospitals in the Children's Hospital Alliance (England) understand their role in relation to socio-economic inequality (e.g. inequalities in income, location, employment, education) where this impacts healthcare access and quality?
- How do the ten children's hospitals in the Children's Hospital Alliance (England) seek to mitigate the impacts of socio-economic inequality on healthcare?
- What are the barriers and enablers to initiatives that mitigate the impacts of socio-economic inequality on healthcare?
- What is perceived as best practice in attempts to mitigate the impacts of socio-economic inequality on healthcare?

Objectives

The objectives identified for the project are:

- Identify variation within governance approaches and policies across the ten children's hospitals in the Children's Hospital Alliance (England), summarising to help facilitate shared learning, by analysing organisational policies.
- Identify areas where there is unmet need for initiatives to reduce socio-economic health inequalities, either generally or due to changes in service delivery since the pandemic, by analysing organisational policies.
- Explore how the ten children's hospitals in the Children's Hospital Alliance (England) understand their role in relation to socio-economic inequality (e.g. inequalities in income, location, employment, education) where this impacts healthcare access and quality, through interviews and/or focus groups with various groups of staff.
- Consider how the ten children's hospitals in the Children's Hospital Alliance (England) are seeking to mitigate the impacts of socio-economic inequality on healthcare, including exploring what barriers and enablers exist, and what is perceived as best practice, through interviews and/or focus groups with various groups of staff.

For peer review only - http://bmjopen.bmj.com/site/about/guidelines.xhtml

Outcome

The primary outcome will be a report to the funder, which contains practical recommendations about the roles that the ten children's hospitals in the Children's Hospital Alliance (England) can play in reducing socio-economic barriers to paediatric care, and in understanding and developing best practice to do this.

4 STUDY DESIGN and METHODS of DATA COLLECTION AND DATA ANALYIS

This project will collect qualitative data via interviews and focus groups, and conduct a scoping review of literature. We will work across the ten partner children's hospitals in the Children's Hospital Alliance (England). We aim to recruit participants as described from each site, as per the funder's aim, but are aware that this may not be possible within the available time. Our aim is to work across all ten locations, with a view to accessing some participants from each category in a minimum of six locations.

The qualitative approach has been selected in order to describe participants' understanding of current policy and practice around socio-economic inequalities and their impact on healthcare, rather than hypothesising about or predicting responses (Kvale & Brinkman, 2014). The qualitative study will gather information and key themes by using semi-structured interviews and focus groups to explore the participants' knowledge of how socio-economic inequalities are considered and managed within their organisation. Rapid Research Evaluation and Appraisal (RREAL) methodologies (Vindrola-Padros, 2021) will be used to analyse data; these methodologies emerge from a qualitative research tradition that recognises the need for rapid analysis of data to produce meaningful recommendations for practice in a timely manner.

Scoping Review: an analysis of the variation in governance approaches and policies around inequalities in children's hospitals to identify areas of best practice.

Academic and grey literature will be accessed by a rapid scoping review. Scoping reviews are used "to map rapidly the key concepts underpinning a research area and the main sources and types of evidence available" (Arksey & O'Malley, 2005) and can be particularly useful when the literature is disparate and has not been previously mapped (Daudt, van Mossel, & Scott, 2013). Recent work has clarified best practices for scoping reviews (Khalil et al., 2016; Peters et al., 2017). From this review, key works will be identified which will be used in analysis of data. This will include key policy documents from the trusts, alongside a grey literature search for national and international secondary healthcare providers to identify areas of best practice.

Qualitative approach: interviews

One interview of approximately 30-40 minutes will take place with each participant (members of the senior leadership team and doctors with an interest in reducing health inequalities) to address the research questions. Informed consent will be given at the start of the interview. We will aim to interview a minimum of six members of the senior leadership team and a minimum of six doctors. We will interview a maximum of thirty members of the senior leadership team and a maximum of thirty doctors. We expect a realistic sample size to be 10-15 members of the senior leadership team and 10-15 doctors.

The interviews will be conducted via telephone or online via Microsoft Teams. If preferred by participants and feasible for the research team, interviews may be conducted face to face. All participants taking part in the interviews must be able and willing to give informed consent.

A draft topic guide for the interviews has been created to guide discussion and maximise relevance and appropriateness. The topic guide will be used in the interviews to provide an initial structure and allow for comparability between respondents, while also allowing for flexibility and individuality in the responses given.

For peer review only - http://bmjopen.bmj.com/site/about/guidelines.xhtml

Interviews will be recorded, and RAP (Rapid Appraisal) sheets will be completed electronically by the researcher undertaking the interview, and one other member of the research team.

Qualitative approach: focus groups

The aim will be to run two focus groups in each children's hospital, each lasting approx. 40-60 minutes. One focus group will be with 4-8 nurses and allied health professionals (e.g. physiotherapists, occupational therapists) and one with 4-8 members of professional, administrative and support staff (e.g. hospital teachers, play leaders, secretaries, receptionists, porters, security, cleaners). Again, our aim is to work across all ten locations, with a view to accessing some participants from each category in a minimum of six locations. This means that we will recruit a minimum of 24 and a maximum of 80 nurses and allied health professionals, and a minimum of 24 and a maximum of 80 professional, administrative and support staff to join focus groups and address the research questions.

The focus groups will be conducted face to face, unless prevented by pandemic infection prevention and control protocols. In this instance, focus groups will be conducted online via Microsoft Teams. All participants taking part in the focus groups must be able and willing to give informed consent.

A draft topic guide for the focus groups has been created to guide discussion and maximise relevance and appropriateness. Focus groups will be recorded, and RAP (Rapid Appraisal) sheets will be completed electronically by the researcher undertaking the interview, and one other member of the research team.

Data Analysis

Rapid assessment procedures aim to produce a rapid but rigorous qualitative analysis (Vindrola-Padros et al, 2020). It is predominantly team based and collaborative in nature, and conducted as an iterative process alongside data collection (Vindrola-Padros, 2021). RAP sheets will be used as the basis for debriefing sessions. Depending on team capacity, one or two RAP sheets will be completed for each interview or focus group (the aim will be two for each where possible). These standardised and structured data collection forms will guide our reflexive analysis process. In group debriefing sessions, we will focus on identifying key themes from these RAP sheets, creating brief summaries of data in tables that will then be used iteratively for cross-case comparison.

Using multiple population groups (senior leaders, doctors, nurses, allied health professionals and professional/ support staff), multiple data sources (published and grey literature, interviews and focus groups) and multiple experienced researchers means that we will be able to triangulate insights (Morse, 2015). Our aim is to produce a targeted analysis that leads to actionable recommendations and enables cross-case comparison between sites to identify good practices (Silverman, 2015).

5 STUDY SETTING

This is a multi-centre research project. The sites are:

- Sheffield Children's NHS Foundation Trust
- Alder Hey Children's Hospital
- Great Ormond Street Hospital
- Birmingham Women's and Children's NHS Foundation Trust
- Royal Manchester Children's Hospital
- Leeds Children's Hospital
- Bristol Royal Hospital for Children
- Evelina London Children's Hospital
- Southampton Children's Hospital
- The Oxford Children's Hospital

For peer review only - http://bmjopen.bmj.com/site/about/guidelines.xhtml

The ten children's hospitals in the Children's Hospital Alliance (England) have been collaborating through the National Paediatric Accelerator Programme to support elective recovery, share best-practice and reduce health inequalities. As a group, these organisations are seeking to develop understanding as to how acute trusts working together, can improve access to quality paediatric healthcare.

6 SAMPLE AND RECRUITMENT

Eligibility Criteria

All participants will have a current role in one of the ten children's hospitals in the Children's Hospital Alliance (England).

Inclusion criteria for the interviews:

1) A member of the senior leadership team (e.g. executive/ non-executive director plus any other relevant senior leader depending on the structure of the organisation) or a doctor with an interest in health inequalities.

2) Over 18 years.

3) Able and willing to give informed consent at the time of the interview.

Inclusion criteria for the focus groups:

1) Nursing and allied health professional staff (e.g. physiotherapists, occupational therapists), professional, administrative and support staff (e.g. hospital teachers, play leaders, secretaries, receptionists, porters, security, cleaners).

2) Over 18 years.

3) Able and willing to give informed consent at the time of the interview.

Exclusion criteria

1) Age <18 years.

2) Does not have a current role as listed above in one of the ten children's hospitals.

4) Unable or unwilling to give informed consent.

Sampling technique

We will work across ten children's hospitals. We aim to recruit participants as described from each site, as per the funder's aim, but are aware that this may not be possible within the available time. Our aim is to work across all ten locations, with a view to accessing some participants from each category in a minimum of six locations. In each organisation, we will sample:

1. 1-3 members of the senior leadership team (one executive and one non-executive director plus any other relevant senior leader depending on the structure of the organisation). All participants will be over 18 years old and recruited based on their professional role. Because of their professional roles, all participants will be English speaking. It is not proposed to exclude on any further criteria. We will recruit a minimum of six and a maximum of 30 people.

2. 1-3 doctors with an interest in reducing health inequalities. All participants will be over 18 years old and recruited based on their professional role. Because of their professional roles, all participants will be English speaking. It is not proposed to exclude on any further criteria. We will recruit a minimum of six and a maximum of 30 people.

For peer review only - http://bmjopen.bmj.com/site/about/guidelines.xhtml

3. 4-8 nurses and allied health professionals (e.g. physiotherapists, occupational therapists). These potential participants will be recruited via their organisations. All participants will be over 18 years old and recruited based on their professional role. Because of their professional roles, all participants will be English speaking. It is not proposed to exclude on any further criteria. We will recruit a minimum of 24 and a maximum of 80 people.

4. 4-8 members of professional, administrative and support staff (e.g. hospital teachers, play leaders, secretaries, receptionists, porters, security, cleaners). These potential participants will be recruited via their organisations. All participants will be over 18 years old and recruited based on their professional role. Because of their professional roles, all participants will be English speaking. It is not proposed to exclude on any further criteria. We will recruit a minimum of 24 and a maximum of 80 people.

Recruitment

Senior leadership team: senior leaders will be identified via our gatekeeper in the National Paediatric Accelerator, who will provide organisational email addresses to the PI. Potential participants will then be provided with a detailed participant information sheet about the study and a copy of the consent form via email, and then asked to participate by Prof Isba as PI, on behalf of the research team. It will be made clear to potential participants that participation is voluntary.

Doctors: Doctors will be identified via our gatekeeper in the National Paediatric Accelerator who will consult each organisation to identify relevant individuals and provide organisational email addresses to the research team. Potential participants will then be provided with a detailed participant information sheet about the study and a copy of the consent form via email, and then asked to participate by one of the research team. It will be made clear to potential participants that participation is voluntary.

Nurses, allied health professionals, professional, administrative and support staff: These staff will be recruited via their organisations. Each children's hospital will be provided with information about the study, and asked to circulate a call for volunteers to participate via their preferred channel (e.g. email lists, newsletters). Potential participants will be asked to email the research team. The research team will then provide a detailed participant information sheet about the study and a copy of the consent form to all interested participants.

No payment will be made to participants.

Consent

Formal written consent supported by a participant information sheet will be sought for the recorded interviews and focus groups. This will be received by the researcher conducting the interview or focus group. On initial agreement by the participant to conduct the interview or focus group they will be provided with a copy of the consent form and participant information sheet. Due to the logistics of the study there will be a period of time between initial agreement and the interview or focus group taking place. This will allow enough time to ensure the participant is entirely happy to be involved and has time to ask questions. Given the study populations, no problems with understanding the consent process are anticipated.

6 ETHICAL AND REGULATORY CONSIDERATIONS

Assessment and management of risk

The research team have all undertaken Good Clinical Practice training, and completed Disclosure and Barring Service checks. As experienced researchers in healthcare, they are experienced in managing safeguarding issues. Limits to confidentiality will be outlined in the participant information sheets,

For peer review only - http://bmjopen.bmj.com/site/about/guidelines.xhtml

stating that if the participant or someone else is at significant risk of harm, the researcher will break confidentiality and speak to a member of the National Paediatric Accelerator Programme. If possible, the researcher will inform the participant before they do this.

All participants will be given a numeric reference/ pseudonym. For the focus groups and interviews, we will anonymise the data (using pseudonyms) in RAP sheets and onwards. Any identifying details (names/ details of incidents) will be anonymised in the report/ any future publications.

Information about confidentiality and its limitations will be included in each Participant Information Sheet, and will be explained to each participant before starting the interview/ focus group. We will verbally remind participants not to share personally identifiable data in focus groups, and not to share focus group content outside the session.

The interviews and focus groups do not cover areas that are potentially sensitive, but the research team are experienced in conducting interviews and focus groups on sensitive topics. We do not anticipate risks to participating in the study. Although participants may find participating interesting, there are no direct benefits in taking part. A Lancaster University Health and Safety assessment has been completed to ensure safety of the research team and participants.

Research Ethics Committee (REC) and other Regulatory review & reports

This study does not require NHS REC approval; however it does require HRA approval via the IRAS system and ethical approval from the Lancaster Faculty of Health and Medicine Ethics Committee. This has been confirmed via the HRA queries helpline.

Before the start of the study, a favourable opinion will be sought from the Lancaster FHM REC for the study protocol, informed consent forms and other relevant documents e.g. advertisements.

Substantial amendments that require review by the REC will not be implemented until that review is in place and other mechanisms are in place to implement at site.

All correspondence with the REC will be retained.

If the study is ended prematurely, the Chief Investigator will notify the REC, including the reasons for the premature termination.

Amendments

Amendments will be managed via the FHM REC application for amendment to previously approved research form. Following approval each site will be informed of the amendment via the research and development manager in each trust.

Amendments will be discussed with the funder and in quarterly oversight meetings.

Amendments will be tracked by keeping an electronic trail of applications and responses to applications for amendments in an access controlled OneDrive folder.

The decision to make an amendment will be taken by the CI with input from the research team.

Protocol compliance

Protocol deviations will be reported in writing to the sponsor (via the Faculty of Health and Medicine REC). Protocol violation will lead to an internal review of protocol with the research team. Recurring protocol deviations will pause the study and lead to substantive changes to the protocol and re-review by the Faculty of Health and Medicine REC.

For peer review only - http://bmjopen.bmj.com/site/about/guidelines.xhtml

End of study

The end of the study is defined as the submission of the final report to the funder.

Data protection and participant confidentiality

All investigators and study site staff will comply with the requirements of the General Data Protection Regulations and the Data Protection Act 2018. All use of personal information during the course of the project will be in accordance with the General Data Protection Regulations and the Data Protection Act 2018. We do not expect to collect any sensitive data.

Personal data (names, job roles, email addresses and phone numbers) will be given by participants when they contact the research team to arrange interviews/ focus groups. This information will only be used to arrange interviews and focus groups, and will be stored in a password-protected spreadsheet in an access-controlled OneDrive folder for the duration of the project. This information will be held separately from the data set. Personal data will only be accessible to the research team. Consent forms will be stored electronically in an access-controlled OneDrive folder, until the end of the project. If consent is taken in paper form, these forms will be scanned and uploaded to the access controlled OneDrive folder, as soon as possible after consent is taken, and originals will be securely shredded.

Lancaster University will be the data controller. For further information about how Lancaster University processes personal data for research purposes, please visit: www.lancaster.ac.uk/research/data-protection

Three sources of data will be gathered and analysed:

1. Interview video recordings.

Because interviews will be conducted via Microsoft Teams, these interviews will be video recorded. It is not possible to only audio record in Teams, so we will need to store video, though these will not be used for analysis. Video recordings will be accessed by another member of the research team, who will listen to the interview and complete a RAP sheet (see 3 below), which will then be stored digitally. Video recordings made on Teams are automatically stored on Lancaster University's Microsoft Stream platform. Only the meeting owner (the researcher/ project administrator) has access to the recording, but will grant access to another member of the research team for analysis (see above). All video recordings will be deleted by the meeting owner within six months of the end of the project (June 2023). From June 2022, automatic storage infrastructure will change, and videos will automatically be stored in OneDrive. The same access restrictions and plans for deletion will apply.

2. Focus group audio recordings. If pandemic protocols mean that focus groups have to be conducted remotely, video recording storage/ collection will apply (see 1 above).

Focus group audio recordings will be copied to the access controlled OneDrive folder and deleted from the capture device on the day of creation or as soon as possible afterwards. This prevents accidental data disclosure and guarantees that the data are backed-up. Digital recordings will be identified with a participant number only. Audio recordings of focus groups will be destroyed within six months of the end of the project. Audio and video recordings will not be transcribed in full, but anonymised quotations identified with a participant number may be transcribed into RAP sheets.

3. RAP sheets, completed by the research team.

RAP (Rapid Appraisal) sheets will be completed electronically in Microsoft Word and stored on the access-controlled OneDrive folder. They will constitute the main source of data from human participants to be retained and will be stored for ten years as per university policy. RAP and RREAL methodologies emerge from a qualitative research tradition that recognises the need for rapid analysis of data to produce meaningful recommendations for practice in a timely manner (Vindrola-Padros et al, 2020). RAP sheets will be used for team debriefs and analysis.

For peer review only - http://bmjopen.bmj.com/site/about/guidelines.xhtml

The researchers may take written notes as an aide-memoire of these focus groups, and of their general impressions and reflections on any informal encounters while onsite, but these notes will not constitute a formal source of data or contain identifiable data. These notes will be destroyed (shredded) following the completion of RAP sheets for the session.

Participants will be informed that they may withdraw from the study at any time before or during the interview/ focus group, and up to two weeks following their interview/ focus group participation without giving any reason. If the request to withdraw comes after data have been anonymised, and incorporated into themes, it might not be possible for it to be withdrawn, though every attempt will be made to extract the data, up to the point of delivering the report to the funder.

Lancaster University will act as the data custodian. Day-to-day data management will be undertaken by the PI. Written/ digital data will be stored in a Onedrive folder with access limited to the project team for ten years after the end of the project. After this, it will be deleted and responsibility for data management will lie with Professor Rachel Isba as principal investigator.

Indemnity

Lancaster University legal liability cover will apply:

- to meet the potential legal liability of the sponsor(s) for harm to participants arising from the management of the research
- to meet the potential legal liability of the sponsor(s) or employer(s) for harm to participants arising from the design of the research
- to meet the potential legal liability of investigators/collaborators arising from harm to participants in the conduct of the research

No arrangement for payment of compensation in the event of harm to the research participants where no legal liability arises has been made.

Personal research devises (laptop, mobile telephone) are insured by the researcher personally.

7 DISSEMINATION POLICY

As a mandatory clause in the consultancy contract, ownership of all intellectual property and data rights for the project will rest with Sheffield Children's Hospital (SCH) (on behalf of all the Trusts participating in the project) and we will not publish any aspect of the research without permission from Sheffield Children's Hospital. SCH agree that permission will not be unreasonably withheld, but data not be disseminated or shared for reuse.

We will produce a report and presentation of findings for the funder. We will discuss possible wider dissemination of selected elements of work via publishing in an academic journal or presenting at a conference with the funder after delivering this report.

8 REFERENCES

Arksey, H., & O'Malley, L. (2005). Scoping studies: towards a methodological framework. International Journal of Social Research Methodology, 8(1), 19–32. https://doi.org/10.1080/1364557032000119616

For peer review only - http://bmjopen.bmj.com/site/about/guidelines.xhtml

Daudt, H. M., van Mossel, C., & Scott, S. J. (2013). Enhancing the scoping study methodology: a large, inter-professional team's experience with Arksey and O'Malley's framework. BMC Medical Research Methodology, 13(1), 48. https://doi.org/10.1186/1471-2288-13-48

Edge Health (2021) "Analysis of inequalities and "was not broughts" for outpatients appointments". (Analysis prepared for the Paediatric Accelerator inequalities group). Unpublished

Khalil, H., Peters, M., Godfrey, C. M., McInerney, P., Soares, C. B., & Parker, D. (2016). An Evidence-Based Approach to Scoping Reviews. Worldviews on Evidence-Based Nursing, 13(2), 118–123. https://doi.org/10.1111/wvn.12144

Kvale, S., & Brinkmann, S. (2014). InterViews. SAGE Publications.

Marmot, M., Allen, J., Goldblatt, P., Herd, E., Morrison, J. (2020). Build Back Fairer: The COVID-19 Marmot Review. The Pandemic, Socioeconomic and Health Inequalities in England. London: Institute of Health Equity

Morse, J. M. (2015). Critical Analysis of Strategies for Determining Rigor in Qualitative Inquiry. Qualitative Health Research, 25(9), 1212–1222. https://doi.org/10.1177/1049732315588501

Peters, M. D., Godfrey, C., McInerney, P., Soares, C. B., Khalil, H., & Parker, D. (2017). Chapter 11: Scoping Reviews. In M. Z. Aromataris E (Ed.), Joanna Briggs Institute Reviewer's Manual. The Joanna Briggs Institute. Retrieved from https://reviewersmanual.joannabriggs.org/

Silverman, D. (2015). Interpreting Qualitative Data. SAGE.

Vindrola-Padros, C., Chisnall, G., Cooper, S., Dowrick, A., Djellouli, N., Symmons, S.M., Martin, S., Singleton, G., Vanderslott, S., Vera, N. and Johnson, G.A., (2020). Carrying out rapid qualitative research during a pandemic: emerging lessons from COVID-19. Qualitative health research, 30(14), pp.2192-2204

Vindrola-Padros, C., (2021) Doing Rapid Qualitative Research. SAGE.

For peer review only

For peer review only - http://bmjopen.bmj.com/site/about/guidelines.xhtml

9. APPENDICES

Appendix 1- Required documentation

- CV R Isba
- CV J Lunn
- CV L Brewster
- CV L Brennan
- Participant Information Sheet interviews
- Participant Information Sheet focus groups
- Consent Form interviews
- Consent Form focus groups
- Email interview invitation
- Focus groups promotional material
- Interview Guide
- Focus group guide
- RAP sheet

Appendix 2 – Schedule of Procedures

- HRA Schedule of Events
- HRA Organisation Information Document
- HRA Is My Study Research Result
- HRA Does My Study Need NHS REC Approval Result

Appendix 3 – Amendment History

No amendments – first submission

Amendment No.	Protocol version no.	Date issued	Author(s) of changes	Details of changes made

For peer review only

For peer review only - http://bmjopen.bmj.com/site/about/guidelines.xhtml

Children's Hospitals Inequalities Research Project

Lancaster University

Interview Schedule

Senior leaders, NEDs and doctors

Welcome and introduction - Seek consent to continue, reminder of audio-recording of interview (/video recording if on Teams), re-cap of project and plan for interview.

The interviews will explore your views on the Trust's policies and practices around health inequalities. We are interested in your perceptions, on the basis of your knowledge and experience. If you do not feel you are able to comment on any area please say so. Do you have any questions before we start?

1. What does the term 'the socio-economic inequalities in paediatric care' mean to you?
    - Prompt: an example of a socio-economic inequality in health is that children from the most deprived or poorest areas have a shorter life expectancy than people in the wealthiest areas.
2. In your experience, does deprivation have an impact on children's health?
    - Prompt: by deprivation we mean things like people living in areas with lower levels of income, employment, higher crime, less education
3. In your experience, does deprivation have an impact on whether or not children access care?
    - Prompt: What stops people from deprived areas from bringing their children to hospital for appointments and care?
4. Do you see health inequalities in the patients using this hospital?
    - What are these? How do they affect care (if not outlined above)
5. What do you see as your role in regard to health inequalities?
6. Doctors only: Are there any processes in place to help you, if you see health inequalities?
    - Prompt: have you received training, are there systems for flagging concerns?
    - Are there any barriers to this? Prompt: E.g. costs of care for patients, timings/ locations of appointments.
7. Does the Trust have a shared organisational view on its role in health inequalities?
    - Prompt: Are you aware of a statement by the board?
8. Are there any organisational policies in place relating to socio-economic barriers to accessing healthcare?
9. Do you think that COVID has affected socioeconomic inequalities?
    - (if yes) What is the Trust doing to address the impacts of COVID?
    - What impact, if any, has COVID had on access and quality at the Trust?

For peer review only - http://bmjopen.bmj.com/site/about/guidelines.xhtml

10. What does the Trust do to reduce health inequalities?
    - What could they do better/ more of?
    - Are there any barriers to this?

11. What would ‘best practice’ in addressing the socio-economic barriers to paediatric care look like?
    - Prompt: Have you seen evidence from elsewhere in the UK or internationally of what works well?

12. Senior leaders only: As an employer, does the Trust have a role in considering in wider society and socio-economic inequalities?

13. Senior leaders only: Any specific questions about hospital policies that have been reviewed for clarification.

Anything not covered?

Is there anything that we haven’t covered in the interview that you think we should know or think about?

Closing and thanks - Thank for their time and contribution.

For peer review only

Children's Hospitals Inequalities Research Project

Lancaster University

Focus Group Schedule

Nurses, Allied Health Professionals

Professional, administrative and support staff

Welcome and introduction - Seek consent to continue, reminder of audio-recording of the group, establish ground rules, re-cap of project and plan for focus group

The focus group will explore your views on the Trust's policies and practices around health inequalities. We are interested in your perceptions, on the basis of your knowledge and experience. Please try not to use any details which might lead someone in the group to be able to identify another staff member/ patient (e.g. their name). If you do not feel you are able to comment on any area please say so. Does anyone have any questions before we start?

1. In your experience, does deprivation have an impact on children's health?
   - Prompt: by deprivation we mean things like people living in areas with lower levels of income, employment, higher crime, less education

2. In your experience, does deprivation have an impact on whether or not children access care?
   - Prompt: What stops people from deprived areas from bringing their children to hospital for appointments and care?

3. Do you see health inequalities in the patients using this hospital?
   - What are these? How do they affect care (if not outlined above)

4. What do you see as your role in regard to health inequalities?

5. Do you know if the Trust has a policy, or a view, on its role in health inequalities?
   - (If yes) What is it? Can you say more?
   - (If no) Why do you think it does not have a policy/ view?

6. Are there any processes in place to help you, if you see health inequalities?
   - Prompt: have you received training, are there systems for flagging concerns?
   - Are there any barriers to this? Prompt: E.g. costs of care for patients, timings/ locations of appointments.

7. What does the Trust do to reduce health inequalities?
   - What could they do better/ more of?

Anything not covered?

Is there anything that we haven't covered in the focus group that you think we should know or think about?

Closing and thanks

Thank for their time and contribution.

For peer review only - http://bmjopen.bmj.com/site/about/guidelines.xhtml

For peer review only

Children's Hospitals Inequalities Research Project

Lancaster University

Study: Children's Hospitals Inequalities Research Project (CHIRP).

Researcher name	
Study location (name of hospital)	
Interview/ focus group (delete as applicable)	If focus group: number of participants =
Participant group (leadership, clinician, other)	
Examples of socio-economic inequalities	
Deprivation: impact on children's health	
Deprivation: impact on access to care	
Deprivation: visibility in their context (hospital)	
Perceived/ outlined role in health inequalities	
Awareness of policies/ policies and organisational views	
Processes in place for support	

For peer review only

Impact of Covid	
What the Trust currently does	
What the Trust might do more of/ better	
What stops the Trust doing more/ better	
Examples of best practice	
Clarification points (policies; <u>SL only</u>)	
Miscellaneous	
Other comments	

For peer review only

Standards for Reporting Qualitative Research (SRQR)*

http://www.equator-network.org/reporting-guidelines/srqr/

Title and abstract

	Page/line no(s).
**Title** - Concise description of the nature and topic of the study Identifying the study as qualitative or indicating the approach (e.g., ethnography, grounded theory) or data collection methods (e.g., interview, focus group) is recommended	1/ 1-3
**Abstract** - Summary of key elements of the study using the abstract format of the intended publication; typically includes background, purpose, methods, results, and conclusions	1/ 5-28

Introduction

**Problem formulation** - Description and significance of the problem/phenomenon studied; review of relevant theory and empirical work; problem statement	2/ 41-84
**Purpose or research question** - Purpose of the study and specific objectives or questions	3/ 85-86

Methods

**Qualitative approach and research paradigm** - Qualitative approach (e.g., ethnography, grounded theory, case study, phenomenology, narrative research) and guiding theory if appropriate; identifying the research paradigm (e.g., postpositivist, constructivist/ interpretivist) is also recommended; rationale**	3/89-100
**Researcher characteristics and reflexivity** - Researchers' characteristics that may influence the research, including personal attributes, qualifications/experience, relationship with participants, assumptions, and/or presuppositions; potential or actual interaction between researchers' characteristics and the research questions, approach, methods, results, and/or transferability	4/ 122-124
**Context** - Setting/site and salient contextual factors; rationale**	3-4/102-120
**Sampling strategy** - How and why research participants, documents, or events were selected; criteria for deciding when no further sampling was necessary (e.g., sampling saturation); rationale**	3-4/102-120 and 4154-158
**Ethical issues pertaining to human subjects** - Documentation of approval by an appropriate ethics review board and participant consent, or explanation for lack thereof; other confidentiality and data security issues	4/143-147
**Data collection methods** - Types of data collected; details of data collection procedures including (as appropriate) start and stop dates of data collection and analysis, iterative process, triangulation of sources/methods, and modification of procedures in response to evolving study findings; rationale**	4/121 - 142

For peer review only - http://bmjopen.bmj.com/site/about/guidelines.xhtml

**Data collection instruments and technologies** - Description of instruments (e.g., interview guides, questionnaires) and devices (e.g., audio recorders) used for data collection; if/how the instrument(s) changed over the course of the study	4/126 and 4/ 136 plus supplementary materials
**Units of study** - Number and relevant characteristics of participants, documents, or events included in the study; level of participation (could be reported in results)	3/108-112
**Data processing** - Methods for processing data prior to and during analysis, including transcription, data entry, data management and security, verification of data integrity, data coding, and anonymization/de-identification of excerpts	4/159-171
**Data analysis** - Process by which inferences, themes, etc., were identified and developed, including the researchers involved in data analysis; usually references a specific paradigm or approach; rationale**	4-5/149-177
**Techniques to enhance trustworthiness** - Techniques to enhance trustworthiness and credibility of data analysis (e.g., member checking, audit trail, triangulation); rationale**	5/172-177

Results/findings

**Synthesis and interpretation** - Main findings (e.g., interpretations, inferences, and themes); might include development of a theory or model, or integration with prior research or theory	5/178-346
**Links to empirical data** - Evidence (e.g., quotes, field notes, text excerpts, photographs) to substantiate analytic findings	5/178-346

Discussion

**Integration with prior work, implications, transferability, and contribution(s) to the field -** Short summary of main findings; explanation of how findings and conclusions connect to, support, elaborate on, or challenge conclusions of earlier scholarship; discussion of scope of application/generalizability; identification of unique contribution(s) to scholarship in a discipline or field	6/347-357 and 6/ 372-385
**Limitations** - Trustworthiness and limitations of findings	6/65-371

Other

**Conflicts of interest** - Potential sources of influence or perceived influence on study conduct and conclusions; how these were managed	Title page
**Funding** - Sources of funding and other support; role of funders in data collection, interpretation, and reporting	Title page

*The authors created the SRQR by searching the literature to identify guidelines, reporting standards, and critical appraisal criteria for qualitative research; reviewing the reference lists of retrieved sources; and contacting experts to gain feedback. The SRQR aims to improve the transparency of all aspects of qualitative research by providing clear standards for reporting qualitative research.

For peer review only

For peer review only - http://bmjopen.bmj.com/site/about/guidelines.xhtml

**The rationale should briefly discuss the justification for choosing that theory, approach, method, or technique rather than other options available, the assumptions and limitations implicit in those choices, and how those choices influence study conclusions and transferability. As appropriate, the rationale for several items might be discussed together.

Reference:

O'Brien BC, Harris IB, Beckman TJ, Reed DA, Cook DA. **Standards for reporting qualitative research: a synthesis of recommendations.** *Academic Medicine*, Vol. 89, No. 9 / Sept 2014 DOI: 10.1097/ACM.0000000000000388

For peer review only

Understanding responsibility for health inequalities in children's hospitals in England: a qualitative study with hospital staff

Journal:	*BMJ Open*
Manuscript ID	bmjopen-2023-081056.R2
Article Type:	Original research
Date Submitted by the Author:	21-Feb-2024
Complete List of Authors:	Brewster, Liz; Lancaster University, Lancaster Medical School<br>Brennan, Louise; Lancaster University Faculty of Health and Medicine<br>Hindocha, Avni; Manchester University NHS Foundation Trust, Royal Manchester Children's Hospital; Lancaster University, Lancaster Medical School<br>Lunn, Judith; Lancaster University<br>Isba, Rachel; Lancaster University Faculty of Health and Medicine
**Primary Subject Heading**:	Paediatrics
Secondary Subject Heading:	Health policy, Public health
Keywords:	Hospitals, Health Equity, PAEDIATRICS, PUBLIC HEALTH, QUALITATIVE RESEARCH

I, the Submitting Author has the right to grant and does grant on behalf of all authors of the Work (as defined in the below author licence), an exclusive licence and/or a non-exclusive licence for contributions from authors who are: i) UK Crown employees; ii) where BMJ has agreed a CC-BY licence shall apply, and/or iii) in accordance with the terms applicable for US Federal Government officers or employees acting as part of their official duties; on a worldwide, perpetual, irrevocable, royalty-free basis to BMJ Publishing Group Ltd ("BMJ") its licensees and where the relevant Journal is co-owned by BMJ to the co-owners of the Journal, to publish the Work in this journal and any other BMJ products and to exploit all rights, as set out in our licence.

The Submitting Author accepts and understands that any supply made under these terms is made by BMJ to the Submitting Author unless you are acting as an employee on behalf of your employer or a postgraduate student of an affiliated institution which is paying any applicable article publishing charge ("APC") for Open Access articles. Where the Submitting Author wishes to make the Work available on an Open Access basis (and intends to pay the relevant APC), the terms of reuse of such Open Access shall be governed by a Creative Commons licence – details of these licences and which Creative Commons licence will apply to this Work are set out in our licence referred to above.

Other than as permitted in any relevant BMJ Author's Self Archiving Policies, I confirm this Work has not been accepted for publication elsewhere, is not being considered for publication elsewhere and does not duplicate material already published. I confirm all authors consent to publication of this Work and authorise the granting of this licence.

For peer review only

UNDERSTANDING RESPONSIBILITY FOR HEALTH INEQUALITIES IN CHILDREN'S HOSPITALS IN ENGLAND: A QUALITATIVE STUDY WITH HOSPITAL STAFF

ABSTRACT

Objectives: To understand how staff in children's hospitals view their responsibility to reduce health inequalities for the children and young people who access their services.

Design: Exploratory qualitative study.

Setting: Nine children's hospitals in England.

Participants: 217 members of staff contributed via interviews and focus groups conducted January-June 2023. Staff were represented at all levels of the organisations, and all staff who volunteered to contribute were included in the study.

Analysis: RREAL methodology for rapid assessment procedures.

Results: All of the children's hospitals were taking some action to reduce health inequalities. Two key themes were identified. First, it was clear that reducing health inequalities was seen as something that was of vital import and should be part of staff's day-to-day activity, framed as 'everyone's business.' Many staff felt there was an obligation to intervene to ensure that children and young people receiving hospital treatment were not further disadvantaged by, e.g. food poverty. Second, however, the deeply entrenched and intersectional nature of health inequalities sometimes meant that these inequalities were complex to tackle, with no clear impetus to specific actions, and could be framed as 'no-one's responsibility'. Within a complex health and social care system, there were many potential actors who could take responsibility for reducing health inequalities, and staff often questioned whether it was the role of a children's hospital to *lead* these initiatives.

Conclusions: Broadly speaking, senior leaders were clear about their organisational role in reducing health inequalities where they impacted on access and quality of care, but there was some uncertainty about the perceived boundaries of responsibility. This led to fragility in the sustainability of activity, and a lack of joined-up intervention. Most hospitals were forging ahead with activity, considering that it was more important to work to overcome health inequalities rather than debate whose job it was.

STRENGTHS AND WEAKNESSES:

- This is the first study to investigate staff's views on health inequalities in children's hospitals in England.
- The sample size of over 200 participants created a substantial dataset for qualitative analysis, which indicates that the findings are robust and transferrable.
- Participants were self-selecting and so may have volunteered to participate because they had a particular interest in sharing views on health inequalities.

For peer review only

UNDERSTANDING RESPONSIBILITY FOR HEALTH INEQUALITIES IN CHILDREN'S HOSPITALS IN ENGLAND: A QUALITATIVE STUDY WITH HOSPITAL STAFF

INTRODUCTION

Definitions of health inequalities vary, but there is broad agreement that these inequalities are unfair and avoidable (1). These inequalities are also systemic or structural rather than purely individual (2,3). Health inequalities are experienced on a social gradient, with those who are more socioeconomically disadvantaged facing greater health challenges, in experiences of illness and access to healthcare (4). In the UK, Michael Marmot and the Institute of Health Equity have been key contributors to driving forward an agenda that recognises the impact of health inequalities on children and young people (5,6). In 2010, the publication of the Marmot Review 'Fair Society, Healthy Lives' (7) set out a framework for action that emphasised how these inequalities accumulate across the lifespan. The recommendations prioritised children as a population group requiring early and meaningful intervention to prevent health inequalities becoming entrenched. This review was updated in 2020, presenting evidence that the gap in health status and outcomes between affluent and socio-economically deprived areas had increased rather than decreased (8).

Children living in areas of high deprivation are more likely to have worse health outcomes (9) and long-term conditions like asthma (10). Access to care also differs, with those from the most deprived areas being less likely to be brought to outpatient appointments (11). Health inequalities therefore represent a tangible issue that needs to be addressed internationally, and have a direct impact on the effectiveness of health service provision.

In the UK, healthcare is publicly-funded and provided free at the point of use, via the National Health Service (NHS). NHS organisations have a statutory duty with regards to specific equality domains defined by the Equality Act 2010 (12), which outlined protected characteristics (e.g. age, sex, race) but there had not been a specific duty on NHS organisations to intervene with regard to health inequalities caused by socio-economic deprivation until the Health and Social Care Act 2012 (13), which introduced the first legal duties relating to health inequalities. Health inequalities are also part of several subsequent national strategies, e.g. the NHS Long Term Plan (14) and the NHS People Plan (15). These top-level strategies focus on a legal duty to reduce inequalities in access, and outcomes for all without specific guidance on how this may be implemented.

Both socio-economic and health inequalities are thought to have increased since the SARS-CoV-2/COVID-19 pandemic, impacting health outcomes, with longer waiting times for elective care. Greater attention has been given to reducing inequalities because of this increase, and the impact on children and young people has been identified as a particular focus for intervention, via initiatives such as NHS England's 'Core20PLUS5' approach (16). This recognises that inequalities are intersectional, focusing on the most deprived 20% of the population, prioritising inclusion, e.g. of people from ethnic minority communities, and identifying five clinical areas of focus: asthma, diabetes, epilepsy care for those with a learning disability, oral health, and mental health.

Within the NHS, children's hospitals provide specialist paediatric care to children and young people, typically up to the age of 16. This provision can be as a stand-alone hospital for children, or as part of a wider organisational structure (NHS trust). There is great diversity amongst service provision (including amongst the sites in this study) because of these organisational structures, but in the main children's hospitals offer services (e.g. emergency and acute general medicine, community and outpatient care) to their local population, and

For peer review only

then specialist services to patients with more complex medical needs (e.g. paediatric cardiac surgery) across a regional or national catchment. Children and young people and their caregivers may therefore travel a great distance to access these more specialist services.

This study aimed to understand how staff in children's hospitals in England view their responsibility to reduce health inequalities for the children and young people they serve.

METHODS

Qualitative data were collected via interviews and focus groups, working across nine children's hospitals in England, as part of a wider research project (supplementary file 1). The qualitative approach was selected to explore participants' understanding of current policy and practice around socio-economic inequalities and their impact on healthcare, rather than hypothesising about, or predicting, responses (17).

Rapid Research Evaluation and Appraisal (RREAL) methodologies (18) were used to collect and analyse data; these methodologies emerge from a qualitative research tradition that recognises the need for rapid data analysis to produce meaningful recommendations for practice in a timely manner. The full process had four stages, in which data were collected, recorded, synthesised, and analysed to produce key recommendations. Using multiple population groups (senior leaders, doctors, nurses, allied health professionals, and professional/ support staff), multiple data sources (interviews and focus groups), and multiple experienced researchers, allowed us to ensure rigour by triangulating insights (19).

Recruitment and participants

Hospitals sampled are all members of the Children's Hospital Alliance, which commissioned a broader research project to exploring the roles that their member organisations can play in reducing socio-economic barriers to paediatric care, and in understanding and developing best practice to do this. At time of the study, the Children's Hospital Alliance had ten members; it now has eleven. As a national network of specialist NHS trusts focused on paediatric care in the UK, it initially focused on support for pandemic recovery for children and young people. One of the original ten member organisations was unable to participate in this study due to capacity. The hospitals varied in geographic location, percentage of local population living in the most 20% of deprived communities, organisational size and structure, and services offered to the paediatric population.

Recruitment was organised by the nine participating organisations who circulated a call to volunteer to participate and organised access to rooms/ teams on site. In total, 217 members of staff were included across the sites (table 1).

For peer review only

Organisation	Number of participants in interviews	Number of focus groups/ conversations and number of participants	Total number of participants per site
Site A	9	4 groups, 19 participants	28
Site B	4	4 groups, 29 participants	33
Site C	7	2 groups, 8 participants	15
Site D	10	4 groups, 12 participants	22
Site E	4	2 groups, 15 participants	19
Site F	11	15 'walk arounds'; 42 participants	53
Site G	6	1 group, 5 participants	11
Site H	5	2 groups, 15 participants	20
Site I	5	2 groups, 11 participants	16
**TOTAL number of participants across sites**	**61**	**39** groups/conversations	**217**

Table 1: Staff participants across nine NHS organisations

Staff were represented at all levels from senior executives to housekeeping staff. Focus groups were composed of staff from similar categories to ensure that those present felt more comfortable to contribute. To maintain anonymity when presenting data we refer to broad three categories of staff:

- Leadership (n=47) - executive and non-executive directors, anyone involved in the management of initiatives concerned with health inequalities;
- Clinical (n=85) - doctors, nurses, and allied health professionals (e.g. physiotherapists, occupational therapists);
- Professional and support staff (n=85) – e.g. receptionists, porters, security, housekeeping.

Data collection

The research team consisted of two academics with expertise in applied qualitative health research, and three research-active public health specialists. We conducted semi-structured interviews with senior leaders and doctors with an interest in health inequalities across nine organisations. Interviews lasted between 30 and 60 minutes. A structured topic guide (supplementary file 2) was used to provide an initial focus and allow for comparability between respondents, while also allowing for flexibility and individuality in the responses given. Interviews were recorded via Microsoft Teams. In total, 61 interviews were completed, and number of interviews per organisation ranged from 4-11.

We conducted focus groups and had informal conversations with clinical, professional and support staff. The majority of focus groups were audio recorded, but for informal conversations, brief written notes with no identifiable details were made and then team members audio-recorded a debrief session to provide an overview of the conversations. One or two focus groups were conducted per organisation, lasting around 60 minutes per group. Number of participants ranged from 3-10 members of staff. A structured topic guide (supplementary file 3) provided focus for discussion. One site advised that informal 'walk around' conversations were more appropriate to engage staff; these discussions were also

For peer review only

structured using the same topic guide. Written informed consent was taken for participation in interviews and focus groups, and verbal consent was taken for ‘walk around’ discussions. All nine organisations were visited for one or two days, meaning additional debrief recordings were often comprehensive and detailed. In total, 39 focus groups and informal discussions were completed. Data were collected between January and June 2023.

Patient and Public Involvement

As part of our wider ongoing programme of work, we have engaged with children and young people and their families to understand barriers to engagement with research around health inequalities (20). This engagement work was conducted concurrently with the study reported here but did not inform data collection or analysis.

Ethical approvals

Ethical approval was granted by FHM Research Ethics Committee Lancaster University on 16 June 2022 (ref: FHM-2022-0844-RECR-3). Health Research Authority approval was granted on 24 August 2022 (ref: IRAS315113 and 22/HRA/3123) and capacity and capability to participate was confirmed by each organisation

Data analysis

Data analysis was conducted in several stages, informed by the RREAL methodology for rapid assessment procedures (RAP) (21).The RAP process is considered an appropriate method to conduct rigorous and robust qualitative data analysis in a short amount of time. It is predominantly team-based and collaborative in nature, and conducted as an iterative process alongside data collection. Data are independently reviewed by multiple team members, before being brought together in a group session for further analysis. RAP is particularly suitable for this type of research, which aims to synthesise diverse perspectives on a defined topic, and is focused on generating useful learning that can be actioned. While it was felt that ‘data saturation’ (22) was being reached at organisations, it is evident that results are intrinsically linked with the sample of people that we spoke to at each hospital.

Following the first stage of data collection, three further stages (multiple RAP sheet generation, individual consolidation and group confirmation) completed the RREAL process. After each interview, focus group, or debrief, a structured RAP sheet (supplementary file 4) outlining the main themes was completed by the researcher leading the data collection. One or more members of the research team then listened to the recording, and independently completed a RAP sheet. RAP sheet generation occurred simultaneously with data collection, with discussion occurring after data collection had been completed. A minimum of two standardised RAP sheets was completed for each interview/focus group, and used to guide our reflexive analysis process. In total, 171 RAP sheets were generated. Researchers then read and reviewed all the RAP sheets generated, and completed independent informal notes to support synthesis. Two members of the team independently generated further ‘site-specific’ RAP sheets, providing an overview of key themes at each organisation. This synthesis process focused on considering the specific context of each organisation as well as the broader project research questions.

We then conducted several day-long group debriefing and analysis sessions, in which we focused on identifying key themes from RAP sheets. This process led to the creation of brief data summaries that were then used iteratively for cross-case comparison. The whole team contributed to these sessions, which aimed to establish consensus, while still being sensitive to diversity of views and experiences in different organisations. Each analysis session ended with confirmation and agreement of key themes.

For peer review only

RESULTS

From our qualitative dataset, including the perspectives of 217 staff across nine organisations, two main themes were identified that help to understand how staff in children's hospitals in England view their responsibility to reduce health inequalities for the children and young people using their services. The first was that staff saw reducing health inequalities as 'everyone's business' and that no child should experience inequalities in care or outcomes because of their socio-economic status. This was particularly visible in terms of the activities that hospitals were undertaking in this space, regardless of national steer and guidance.

However, the second theme – whilst also recognising that health inequalities were deeply entrenched and intersectional – suggested that these inequalities were seen as 'no-one's responsibility' or 'not part of core business' for a children's hospital. Within a complex health and social care system, there were many potential actors who could take responsibility for reducing health inequalities, and staff often questioned whether it was the role of a children's hospital to lead these initiatives rather than treating the presenting illness. This led to some fragility in the sustainability of initiatives.

Nevertheless, all of the children's hospitals were taking some action to reduce health inequalities. Many staff felt there was an obligation to intervene to ensure that children and young people receiving hospital treatment (and their caregivers) were not further disadvantaged by, e.g. food poverty.

Health inequalities as 'everyone's business'

The theme of preventing health inequalities as something that should be 'everyone's business' was observed in discussion of the strategy and day-to-day activities of each organisation. Presentation of this theme considers how 'responsibility' was understood in relation to organisational priorities, and the challenges to taking up this responsibility. We pay particular attention to communication, formal and informal support, and the impact of the pandemic on providing additional evidence of the impact of these inequalities, which built the case for intervention.

All participants were asked about their knowledge of organisational policies and strategies that related to health inequalities. Over half of the nine organisations already had a health inequalities strategy in place, although for larger organisations who also offered adult provision, this was an organisation-wide strategy rather than child-focused. The remaining organisations all discussed making progress towards a strategy. The majority of organisations had a health inequalities steering group and/or forum. More than half had a data dashboard, or made reference to data analysis activity, and this was used to try to identify patients at risk of not attending appointments. This activity was led at an organisational level, rather than linked to national strategy.

Some participants were clear about the organisational strategy around health inequalities, but others were less certain what the overall vision was, or what activities connected to it. In some organisations, there was a named individual with responsibility for health inequalities, but this was not universal, and often, people working on the 'shop floor' were less familiar with the strategic direction. We found that awareness varied in terms of staff knowledge of strategy and policy around health inequalities. While this finding was heavily dependent upon the sample asked, each site had some staff who were unaware of strategy or activity. It became clear that there was a lack of clear communication about work on health inequalities.

For peer review only

> *'It's frustrating as a clinician… I asked my leads and they said they didn't know, there's all these layers of people who didn't know… eventually I found out about a steering group. But it's not affecting my practice what they're doing [about reducing inequalities].* (Clinical, site B)

This lack of communication was one example of how work to reduce health inequalities was fragile. It was often led by keen individuals, and so while health inequalities were seen as something that should be a focus for activities ('everyone's business'), this could be more at the level of a vision that informed activities rather than something with concrete key performance indicators .

Consideration of reduction of health inequalities was clearly visible in interventions led by the organisations. There were several common forms of support across organisations, almost all of these focused on meeting day-to-day basic needs. These included cheap food offers, free food for breastfeeding mothers, free sanitary products, parking/travel reimbursement and hospital transport for specific identified groups, accommodation, and interpreters. While available in the majority of organisations, these basic offers were not universal across all sites and specialties. Other initiatives which occurred less frequently included food vouchers, a universal patient transport offer, and free food on wards for household members.

As well as strategic direction and organisationally-initiated formal support for families, a second, more hidden layer of support was present in many of the organisations. This informal system was largely driven by individuals, often at nursing or domestic support level, and showed how reducing health inequalities permeated into every level of work. Stories of housekeeping staff taking washing from families, buying caregivers coffees and food, and giving leftover food to families were frequent. Similarly, nursing staff discussed ways to order additional food when they noticed someone was in need. In some hospitals, ward managers had access to a budget that funded food vouchers and taxis. This support was partly dependent upon motivated and caring individuals.

> *'I think the people that I speak to, and I speak to a broad range, it's actually part of their core value rather than something that they think would make a good headline. And I think that resonates throughout the entire organization that they would be doing this even if no one was watching them do it.'* (Leadership, site F)

Some staff felt that there needed to be key performance indicators around health inequalities to encourage prioritisation of resource to this area of activity. With no current metrics around health inequalities, it becomes one of the 'nice to haves' rather than an essential.

> *'We don't have standards for the level of support for our families and we should do shouldn't we?'* (Leadership, site D)

> *'We can't allow health inequalities to become a luxury activity for organisations that happen to have the resources.'* (Leadership, site G)

Some staff explained that while health inequalities were not new, the pandemic had highlighted inequalities in outcomes and provided evidence (and therefore understanding) of the health inequalities that passionate members of staff had been trying to explain previously.

> *'I'm sure if you'd have talked about some of this stuff five years ago people wouldn't have understood as much as they do now.'* (Clinical, site A)

For peer review only

It was felt that the pandemic had also shone a focus on inequalities, kickstarting projects such as analysis of data on potential inequalities.

> *'There's variation in how long certain groups are waiting for care… we've observed in our analysis those with the lowest income wait on average six years longer. Children and young people with a learning disability wait longer to complete their treatment. Both of those factors were there before Covid, but now we have done the analysis and are looking at how we can address it.'* (Leadership, site B)

Most of the leadership and clinical staff we interviewed were vocal about the way that health inequalities were their business, and affected their day-to-day provision of healthcare.

> *'Because if they don't have a good start, they're not gonna have a long and happy life. So they will have poor diet. Doesn't matter what people tell you. It is quite expensive to eat well, so they will have increased diabetes. They will have increased obesity. They'll have all the pitfalls of living in a cold, damp house. So they'll have worse asthma.'* (Leadership, site F)

> *'Making people realise that being curious about why a certain child keeps coming back is just as much my responsibility as a doctor as it is to prescribe the Ventolin Inhaler.'* (Clinical, site G)

This idea of 'prevention rather than cure' was epitomised in several examples offered of activity to reduce health inequalities. For example, one clinician discussed how the impacts of having a domestic violence prevention service piloted in their Paediatric Emergency Department might have wide-ranging impacts for children and young people attending the department, but that those effects might be seen in educational attainment in a generation's time rather than being immediately visible.

Health inequalities as 'no-one's responsibility'

In contrast to the above discussion of health inequalities as a priority area when inequalities impacted access and quality of care, this theme highlights how staff varied in their articulation of this priority in practice. Unclear boundaries between the hospital and other services, a lack of a national steer, challenges around wider system connections, concerns about potential cost and a lack of 'embeddedness' to make tackling inequalities part of core work were seen as challenges.

Although, broadly speaking, members of leadership teams were clear about their role in overcoming socio-economic inequalities where they impacted on access and quality of care, there was some uncertainty about the perceived boundaries of responsibility with other institutions (e.g. local government, other healthcare services). Some staff saw that leading the health inequalities agenda was not the role of the hospital, and there was an awareness that other services such as local authorities, public health, schools, community care, and general practice also had a role to play.

> *'So whose responsibility is it to do this? One answer is everyone's responsibility. Another answer is not ours. And our job is here to see and treat people in front of us. That is one response. And you think well, there are public health teams around. There's lots of stuff. Should we be out there in the city centre running our campaigns about how to brush teeth? Or should we put a dentist in theatre doing the dental extractions? That's the choice.'* (Leadership, site F)

For peer review only

Staff at all levels felt that a key barrier to addressing health inequalities action was the lack of national and local priority given to the subject. Senior executives were honest that priority was often given to the national targets such as waiting lists and finances.

> *'It's not the Government's priority. No government is interested in long-term gain. They're interested in waiting lists and how they got better during their jurisdiction.'* (Leadership, site D)

Although some senior staff spoke about working as part of a system, staff members at all levels, and across all organisations, spoke about their frustrations in not feeling part of the system. This was seen as a barrier to advancing the inequalities agenda for children and young people.

> *'It's being holistic as a system that we're not very good at doing. Because we are separate to social services and we're separate to schools, we don't think of 'children's' as a system yet.'* (Leadership, site D)

This was compounded by the inability of IT systems to link up the health and social care system.

> *'No one has time or capacity to make these sort of links happen. It is just not at the top of their agenda to try and get everyone equitable access in one area. You can see a massive discrepancy across the region and actually it does have an impact on death rates in our service.'* (Clinical, site I)

For some organisations, their status within the community as a 'trusted brand' meant that they felt it was within their remit to intervene, regardless of their 'responsibility.' Children's hospitals were seen as well-placed to lead on and communicate upon health inequalities to the wider public.

> *'We've got a trusted brand and … it's well known, it's well understood. And if we went walking up and down the high street with donation boxes, people go oh yeah, I'll give money to the children's hospital.'* (Leadership, Site F)

The picture was more mixed in terms of the views of the broader staff cohort. Some staff saw support for overcoming health inequalities as part of their role, but others felt it was not a priority, or felt less confident in asking questions about deprivation and need. Most health inequalities work was being driven by passionate individuals, making it unsustainable if these people leave. These champions were a positive, in terms of driving action, but their impact was limited by the way in which health inequalities were not part of core business or communicated within their organisations, and often went unnoticed by staff at leadership level. The lack of embeddedness meant that even if the aim was for every member of staff to take responsibility for tackling the health inequalities they encountered, they were not held responsible for delivering this outcome.

A smaller number of staff were concerned about the overall expense of initiatives such as free food and transport, particularly if they were promoted widely. They did not see support for patients as a key role for the organisations, and highlighted the potential for initiatives to be inappropriately accessed by people who did not need the support. They also recognised the impact of increased workload, for example if families were fed alongside patients, and noted that this could be time-consuming. It became clear that for inequalities to become part of core business, a significant shift was required.

For peer review only

DISCUSSION

This paper has presented findings from a large-scale qualitative study aiming to understand how staff in children's hospitals in England view their responsibility to reduce health inequalities for the children and young people who access their services. Key findings were that while reducing health inequalities was broadly seen as important to these organisations, there were a number of barriers to activity, including a lack of agreement about the scope and remit of intervention, and whose priority it was to lead on these interventions. There was little specific national guidance to support decisions around how to manage inequalities in access and outcomes, but organisations could see the consequences of a lack of intervention on a daily basis. Although it was a legal duty, it was one of many competing priorities. Therefore, discussions about when, where, and how to intervene often centred on questions around best use of available resource, and how impact of resource use could not be evidenced or tied to organisational key priorities.

Strengths of this study are that it is, to our knowledge, the first study to investigate staff's views on health inequalities in children's hospitals in England or elsewhere. The sample size of over 200 participants created a substantial dataset for qualitative analysis, which indicates that the findings are robust and transferrable.

Weaknesses include that participants were self-selecting and so may have volunteered to participate because they had a particular interest in sharing views on health inequalities. Rapid qualitative methods with selected transcription and analysis directly from audio recording were used, rather than full transcription of all interviews/ focus groups (21). This may be considered a weakness, but has been accounted for in the rigorous process conducted by the team, where recordings were reviewed multiple times by different researchers and a structured recording form was used to capture data of interest. As all data were recorded, there was opportunity to return to the transcripts as needed throughout the analysis process. Due to the remit of the funding for this research, it was not possible to involve patients or the public in the design, conduct, reporting, or dissemination plans of our research, though our wider research has a strong commitment to this aim. This meant we were also not able to speak to children, young people and families as part of this research, meaning their voices are represented by others within this dataset, which is a weakness of the study. Future research should address these questions around how health inequalities impact on healthcare from the perspective of children and young people in particular.

As identified in a recent scoping review protocol (23), and the review itself (24) there are few academic journal papers that outline activities and interventions conducted by children's hospitals to reduce health inequalities, and more transferrable evidence can be found in the grey literature (defined as sources of literature that have not been formally published). This is partly due to the more informal way that applied intervention and improvement work is often shared in the healthcare sector. Despite the acknowledged importance of reducing health inequalities for children and young people, little research has been conducted in the UK, and this is the first that has sought to understand how staff within NHS organisations perceive their role. This paper therefore makes an important contribution to understanding barriers and challenges around activities to reduce health inequalities and how hospital staff at all levels may contribute to these activities.

A recent review (25) aimed to outline 'the importance of healthcare workers advocating for structural and high-level policy change to address the deep-rooted societal problems that cause child poverty' by proposing a framework to address health inequalities experienced by children. Its broad conclusions support our more detailed account of the entrenched and intersectional nature of health inequalities and how they might be addressed within a

For peer review only

children's hospital context. In a US context, similar questions are being raised about the potential role of children's hospitals in reducing health inequalities more usually addressed in a community setting (26). A first next step may be to work directly with children and young people in future research to understand more about the challenges they face in relation to these health inequalities as experienced in everyday life.

CONCLUSION AND RECOMMENDATIONS

While there is some clarity for organisations about their role in reducing health inequalities where they impacted on access and quality of care, there remains uncertainty about tackling health inequalities around the wider determinants of health, and the perceived boundaries of responsibility with other organisations within the wider health and social care system. In this study, most hospitals were forging ahead with activity on selected wider determinants, considering that it was more important to work to overcome health inequalities rather than debate whose job it was. While this is laudable, and the contribution of passionate individuals should not be underestimated, it does not position health inequalities clearly as part of children's hospitals' everyday work and so the sustainability of interventions is not guaranteed. The needs of children and young people require much greater attention and stronger, clearer policy at a national level.

Contributorship statement

Isba, Brewster, Brennan and Lunn conceptualised and designed the work. Pearcy was responsible for acquisition of data. Isba, Brewster, Brennan, Broad, Hindocha, Lunn McCreddin and Patel collected the data. Isba, Brewster, Brennan, Hindocha and Lunn analysed the data. Brewster prepared the original draft. Brennan and Isba critically reviewed and edited the work. All authors agreed the final version and are accountable for the accuracy and integrity of the work.

Competing interests

The authors declare that they have no known competing financial interests or personal relationships that could have appeared to influence the work reported in this paper. The views expressed are those of the author(s) and not necessarily those of the Children's Hospital Alliance.

Funding

This work was supported by the National Paediatric Accelerator Programme, now the Children's Hospital Alliance, contracted by Sheffield Children's Hospital, grant number SCH5628.

Data sharing statement

All data relevant to the study are included in the article or uploaded as supplementary information.

CONTRIBUTORS

Eleanor Broad, Dumfries and Galloway Royal Infirmary, UK

Nicola McCreddin, Manchester Royal Infirmary, UK

Pallavi Patel, St Helens and Knowsley Teaching Hospitals NHS Trust, UK

Julie Pearcy, Lancaster Medical School, UK

For peer review only

Acknowledgements

Thank you to everyone at the hospital trusts who helped us arrange interviews and focus groups, and everyone who kindly gave up their time to contribute to our research. Special thanks to Anne Marie Davies, Children's Hospital Alliance and Alder Hey Children's Hospital, Rekel Kerr, Children's Hospital Alliance and Birmingham Children's Hospital and Alexandra Norrish, Children's Hospital Alliance and Sheffield Children's Hospital. Thanks to Tracy Briggs, Heather Catt, Fiona Egboko, Imelda Mayor, Nicola McCreddin, Pallavi Patel, Dora Pestotnik Stres, Esther Primrose and Andrew Rowland for contributions to the wider project.

REFERENCES

1. McCartney G, Popham F, McMaster R, Cumbers A. Defining health and health inequalities. Public Health [Internet]. 2019;172:22–30. Available from: https://www.sciencedirect.com/science/article/pii/S0033350619301076

2. Whitehead M. The concepts and principles of equity and health. Int J Health Serv. 1992;22(3):429–45.

3. Graham H. Social Determinants and Their Unequal Distribution: Clarifying Policy Understandings. Milbank Q [Internet]. 2004 Mar 1;82(1):101–24. Available from: https://doi.org/10.1111/j.0887-378X.2004.00303.x

4. Donkin AJM. Social Gradient. In: The Wiley Blackwell Encyclopedia of Health, Illness, Behavior, and Society [Internet]. 2014. p. 2172–8. Available from: https://doi.org/10.1002/9781118410868.wbehibs530

5. Marmot M. The health gap: The challenge of an unequal world. London: Bloomsbury; 2015.

6. Marmot M. Social determinants of health inequalities. Lancet. 2005;365(9464):1099–104.

7. Marmot M, Allen J, Goldblatt P, Boyce T, McNeish D, Grady M, et al. Fair society, healthy lives: The Marmot Review [Internet]. Vol. 0. London; 2010. Available from: http://dx.doi.org/10.1016/j.puhe.2012.05.014

8. Marmot M, Allen J, Boyce T, Goldblatt P, Morrison J. Health Equity in England : The Marmot Review Ten Years on. London; 2020.

9. Lai ETC, Wickham S, Law C, Whitehead M, Barr B, Taylor-Robinson D. Poverty dynamics and health in late childhood in the UK: evidence from the Millennium Cohort Study. Arch Dis Child [Internet]. 2019 Nov 1;104(11):1049 LP – 1055. Available from: http://adc.bmj.com/content/104/11/1049.abstract

10. Wickham S, Anwar E, Barr B, Law C, Taylor-Robinson D. Poverty and child health in the UK: using evidence for action. Arch Dis Child. 2016 Aug;101(8):759–66.

11. French LR, Turner KM, Morley H, Goldsworthy L, Sharp DJ, Hamilton-Shield J. Characteristics of children who do not attend their hospital appointments, and GPs' response: a mixed methods study in primary and secondary care. Br J Gen Pract. 2017 Jul;67(660):e483–9.

12. HM Government. Equality Act [Internet]. 2010. Available from: https://www.legislation.gov.uk/ukpga/2010/15/contents

13. HM Government. Health and Social Care Act 2012 [Internet]. 2012. Available from: https://www.legislation.gov.uk/ukpga/2012/7/contents/enacted

14. NHS England. The NHS Long Term Plan [Internet]. 2019. Available from: https://www.longtermplan.nhs.uk/publication/nhs-long-term-plan/

For peer review only

15. NHS England. WE ARE THE NHS: People Plan for 2020/2021 - action for us all [Internet]. London; 2020. Available from: https://www.england.nhs.uk/ournhspeople/

16. NHS England. Core20PLUS5 – An approach to reducing health inequalities for children and young people [Internet]. 2023 [cited 2023 Aug 9]. Available from: https://www.england.nhs.uk/about/equality/equality-hub/national-healthcare-inequalities-improvement-programme/core20plus5/core20plus5-cyp/

17. Kvale S, Brinkmann S. InterViews: learning the craft of qualitative research interviewing [2nd edition]. London: Sage; 2009.

18. Vindrola-Padros C, Chisnall G, Cooper S, Dowrick A, Djellouli N, Symmons SM, et al. Carrying Out Rapid Qualitative Research During a Pandemic: Emerging Lessons From COVID-19. Qual Health Res. 2020 Dec;30(14):2192–204.

19. Morse JM. Critical Analysis of Strategies for Determining Rigor in Qualitative Inquiry. Qual Health Res. 2015 Sep;25(9):1212–22.

20. Hindocha A, Brennan L, Brewster L, Lunn J, Isba R. The wider health and wellbeing needs of those accessing paediatric care in England: engaging with the hidden voices of children and young people. Lancet [Internet]. 2023;402:S50. Available from: https://www.sciencedirect.com/science/article/pii/S0140673623021049

21. Vindrola-Padros C. Doing Rapid Qualitative Research. London: Sage Publication; 2021.

22. Charmaz K. Constructing Grounded Theory: a practical guide through qualitative analysis. London: Sage; 2006.

23. Brennan L, Brewster L, Lunn J, Egboko F, Stres DP, Patel P, et al. How do hospitals address health inequalities experienced by children and young people: a grey literature scoping review protocol. BMJ Open [Internet]. 2023 Apr 1;13(4):e071682. Available from: http://bmjopen.bmj.com/content/13/4/e071682.abstract

24. Brennan L, Pestotnik Stres D, Egboko F, Broad E, Brewster L, Lunn J, et al. How do children's hospitals address health inequalities: a grey literature scoping review. BMJ Open. 2023;

25. Lee AR, Kingdon CC, Davie M, Hawcutt D, Sinha IP. Child poverty and health inequalities in the UK: A guide for paediatricians. Arch Dis Child. 2022;108(2):94–101.

26. Franz B, Cronin CE. Are Children's Hospitals Unique in the Community Benefits They Provide? Exploring Decisions to Prioritize Community Health Needs Among U.S. Children's and General Hospitals. Front Public Heal. 2020;8(February):1–10.

For peer review only

FULL/LONG TITLE OF THE STUDY
Children's Hospitals Inequalities Research Project

SHORT STUDY TITLE / ACRONYM
CHIRP

PROTOCOL VERSION NUMBER AND DATE
V1.0 21 April 2022

RESEARCH REFERENCE NUMBERS

**IRAS Number:**	315113
**FUNDERS Number:**	SCH5628 Sheffield Children's Hospitals NHS Foundation Trust (SCH)<br>National Paediatric Accelerator

For peer review only

For peer review only - http://bmjopen.bmj.com/site/about/guidelines.xhtml

STUDY PROTOCOL

Children's Hospitals Inequalities Research Project

1 BACKGROUND

This research will investigate how the ten children's hospitals in the Children's Hospital Alliance (England) view their responsibility to reduce socio-economic health inequalities (e.g. income, location, employment, education, cultural and religious barriers) and how these translate into actions. As part of the National Paediatric Accelerator Programme, we have been asked to look at organisational policies (collect documents) and talk to different groups of staff (in interviews and focus groups) about inequalities experienced by patients, how these affect care (e.g. by being able to attend appointments) and to identify recommendations to develop good practice to ultimately reduce the number of 'Was not Broughts' (the children's equivalent of "Did not attend"), and improve care.

This study will collect qualitative data via interviews and focus groups, which will take place online or at the participants' workplaces. NHS staff participants will be recruited via their organisations. They will include members of the senior leadership team (e.g. executive/ non-executive director plus any other relevant senior leader depending on the structure of the organisation), doctors with an interest in health inequalities, nursing and allied health professional staff (e.g. physiotherapists, occupational therapists), and professional, administrative and support staff (e.g. hospital teachers, play leaders, secretaries, receptionists, porters, security, cleaners).

2 RATIONALE

The ten children's hospitals involved in the study have been collaborating through the National Paediatric Accelerator Programme to support elective recovery, share best practice and reduce health inequalities. As a group, the organisations are seeking to develop understanding as to how acute trusts working together, can improve access to quality paediatric healthcare. The work has included looking at the inequitable impact that the SARS-CoV-2/COVID pandemic has had on the health and wellbeing of children from the poorest backgrounds. In particular, focus has been on the disproportionate impact that deprivation has had on the ability of children from different socioeconomic backgrounds to access healthcare. Children from the most deprived decile in the index of multiple deprivation are more than twice as likely to not be brought to their outpatients' appointments (Edge Health, 2021) than those from the least deprived decile.

It is relatively new for NHS trusts to think about, and seek to take action on, health inequalities relating to deprivation. In considering its role in inequalities, the NHS is most familiar with its statutory role with regards to some specific equality domains. The Equality Act 2010 defines several protected characteristics (i.e. age, sex, race, disability, sexual orientation, gender reassignment, marriage and civil partnerships, pregnancy and maternity, religion or belief discrimination). By comparison with the protected characteristics, there is no specific duty on NHS organisations to intervene with regard to deprivation. However, the Health and Social Care Act 2012 introduced the first legal duties about health inequalities. Health inequalities are also part of several national strategies, such as the NHS Long Term Plan and the NHS People Plan. Initial analysis suggests that that socio-economic factors can have significant impacts on the ability of children and their parents, and recent analysis suggests that the situation has worsened during the pandemic (Marmot et al, 2020).

This research is part of the National Paediatric Accelerator Programme, which includes the remit to reduce the barriers to families preventing them from attending appointments with a view to ultimately reducing the number of 'Was not Broughts', which will support the elective recovery plan for Paediatrics. The research project will explore the roles that these ten trusts can play in reducing socio-economic barriers to paediatric care, and in understanding and developing best practice to do this.

Literature review

As part of the research project, we will conduct a literature-based analysis of the variation in governance approaches and policies around inequalities. This will include documents e.g. policies

For peer review only - http://bmjopen.bmj.com/site/about/guidelines.xhtml

from the ten partner hospitals, published literature including how inequalities are experienced by children and young people in the UK, regional variation, impact of the SARS-CoV-2/COVID19 pandemic, grey literature around inequalities experienced by CYP, including identification of any global exemplars of how to reduce health inequalities.

3 RESEARCH QUESTION/AIM(S)

- How do the ten children's hospitals in the Children's Hospital Alliance (England) view their responsibility to reduce socio-economic health inequalities (e.g. income, location, employment, education, cultural and religious barriers)?
- How does this view of the potential responsibility to reduce socio-economic health inequalities translate into actions in the ten children's hospitals in the Children's Hospital Alliance (England)?
- Is there variation within governance approaches and policies across the ten children's hospitals in the Children's Hospital Alliance (England)?
- Are there areas where there are unmet needs for initiatives to reduce socio-economic health inequalities, either generally or due to changes in service delivery since the pandemic?
- How do the ten children's hospitals in the Children's Hospital Alliance (England) understand their role in relation to socio-economic inequality (e.g. inequalities in income, location, employment, education) where this impacts healthcare access and quality?
- How do the ten children's hospitals in the Children's Hospital Alliance (England) seek to mitigate the impacts of socio-economic inequality on healthcare?
- What are the barriers and enablers to initiatives that mitigate the impacts of socio-economic inequality on healthcare?
- What is perceived as best practice in attempts to mitigate the impacts of socio-economic inequality on healthcare?

Objectives

The objectives identified for the project are:

- Identify variation within governance approaches and policies across the ten children's hospitals in the Children's Hospital Alliance (England), summarising to help facilitate shared learning, by analysing organisational policies.
- Identify areas where there is unmet need for initiatives to reduce socio-economic health inequalities, either generally or due to changes in service delivery since the pandemic, by analysing organisational policies.
- Explore how the ten children's hospitals in the Children's Hospital Alliance (England) understand their role in relation to socio-economic inequality (e.g. inequalities in income, location, employment, education) where this impacts healthcare access and quality, through interviews and/or focus groups with various groups of staff.
- Consider how the ten children's hospitals in the Children's Hospital Alliance (England) are seeking to mitigate the impacts of socio-economic inequality on healthcare, including exploring what barriers and enablers exist, and what is perceived as best practice, through interviews and/or focus groups with various groups of staff.

For peer review only - http://bmjopen.bmj.com/site/about/guidelines.xhtml

Outcome

The primary outcome will be a report to the funder, which contains practical recommendations about the roles that the ten children's hospitals in the Children's Hospital Alliance (England) can play in reducing socio-economic barriers to paediatric care, and in understanding and developing best practice to do this.

4 STUDY DESIGN and METHODS of DATA COLLECTION AND DATA ANALYIS

This project will collect qualitative data via interviews and focus groups, and conduct a scoping review of literature. We will work across the ten partner children's hospitals in the Children's Hospital Alliance (England). We aim to recruit participants as described from each site, as per the funder's aim, but are aware that this may not be possible within the available time. Our aim is to work across all ten locations, with a view to accessing some participants from each category in a minimum of six locations.

The qualitative approach has been selected in order to describe participants' understanding of current policy and practice around socio-economic inequalities and their impact on healthcare, rather than hypothesising about or predicting responses (Kvale & Brinkman, 2014). The qualitative study will gather information and key themes by using semi-structured interviews and focus groups to explore the participants' knowledge of how socio-economic inequalities are considered and managed within their organisation. Rapid Research Evaluation and Appraisal (RREAL) methodologies (Vindrola-Padros, 2021) will be used to analyse data; these methodologies emerge from a qualitative research tradition that recognises the need for rapid analysis of data to produce meaningful recommendations for practice in a timely manner.

Scoping Review: an analysis of the variation in governance approaches and policies around inequalities in children's hospitals to identify areas of best practice.

Academic and grey literature will be accessed by a rapid scoping review. Scoping reviews are used "to map rapidly the key concepts underpinning a research area and the main sources and types of evidence available" (Arksey & O'Malley, 2005) and can be particularly useful when the literature is disparate and has not been previously mapped (Daudt, van Mossel, & Scott, 2013). Recent work has clarified best practices for scoping reviews (Khalil et al., 2016; Peters et al., 2017). From this review, key works will be identified which will be used in analysis of data. This will include key policy documents from the trusts, alongside a grey literature search for national and international secondary healthcare providers to identify areas of best practice.

Qualitative approach: interviews

One interview of approximately 30-40 minutes will take place with each participant (members of the senior leadership team and doctors with an interest in reducing health inequalities) to address the research questions. Informed consent will be given at the start of the interview. We will aim to interview a minimum of six members of the senior leadership team and a minimum of six doctors. We will interview a maximum of thirty members of the senior leadership team and a maximum of thirty doctors. We expect a realistic sample size to be 10-15 members of the senior leadership team and 10-15 doctors.

The interviews will be conducted via telephone or online via Microsoft Teams. If preferred by participants and feasible for the research team, interviews may be conducted face to face. All participants taking part in the interviews must be able and willing to give informed consent.

A draft topic guide for the interviews has been created to guide discussion and maximise relevance and appropriateness. The topic guide will be used in the interviews to provide an initial structure and allow for comparability between respondents, while also allowing for flexibility and individuality in the responses given.

For peer review only - http://bmjopen.bmj.com/site/about/guidelines.xhtml

Interviews will be recorded, and RAP (Rapid Appraisal) sheets will be completed electronically by the researcher undertaking the interview, and one other member of the research team.

Qualitative approach: focus groups

The aim will be to run two focus groups in each children's hospital, each lasting approx. 40-60 minutes. One focus group will be with 4-8 nurses and allied health professionals (e.g. physiotherapists, occupational therapists) and one with 4-8 members of professional, administrative and support staff (e.g. hospital teachers, play leaders, secretaries, receptionists, porters, security, cleaners). Again, our aim is to work across all ten locations, with a view to accessing some participants from each category in a minimum of six locations. This means that we will recruit a minimum of 24 and a maximum of 80 nurses and allied health professionals, and a minimum of 24 and a maximum of 80 professional, administrative and support staff to join focus groups and address the research questions.

The focus groups will be conducted face to face, unless prevented by pandemic infection prevention and control protocols. In this instance, focus groups will be conducted online via Microsoft Teams. All participants taking part in the focus groups must be able and willing to give informed consent.

A draft topic guide for the focus groups has been created to guide discussion and maximise relevance and appropriateness. Focus groups will be recorded, and RAP (Rapid Appraisal) sheets will be completed electronically by the researcher undertaking the interview, and one other member of the research team.

Data Analysis

Rapid assessment procedures aim to produce a rapid but rigorous qualitative analysis (Vindrola-Padros et al, 2020). It is predominantly team based and collaborative in nature, and conducted as an iterative process alongside data collection (Vindrola-Padros, 2021). RAP sheets will be used as the basis for debriefing sessions. Depending on team capacity, one or two RAP sheets will be completed for each interview or focus group (the aim will be two for each where possible). These standardised and structured data collection forms will guide our reflexive analysis process. In group debriefing sessions, we will focus on identifying key themes from these RAP sheets, creating brief summaries of data in tables that will then be used iteratively for cross-case comparison.

Using multiple population groups (senior leaders, doctors, nurses, allied health professionals and professional/ support staff), multiple data sources (published and grey literature, interviews and focus groups) and multiple experienced researchers means that we will be able to triangulate insights (Morse, 2015). Our aim is to produce a targeted analysis that leads to actionable recommendations and enables cross-case comparison between sites to identify good practices (Silverman, 2015).

5 STUDY SETTING

This is a multi-centre research project.

For peer review only

For peer review only - http://bmjopen.bmj.com/site/about/guidelines.xhtml

The ten children's hospitals in the Children's Hospital Alliance (England) have been collaborating through the National Paediatric Accelerator Programme to support elective recovery, share best-practice and reduce health inequalities. As a group, these organisations are seeking to develop understanding as to how acute trusts working together, can improve access to quality paediatric healthcare.

6 SAMPLE AND RECRUITMENT

Eligibility Criteria

All participants will have a current role in one of the ten children's hospitals in the Children's Hospital Alliance (England).

Inclusion criteria for the interviews:

1) A member of the senior leadership team (e.g. executive/ non-executive director plus any other relevant senior leader depending on the structure of the organisation) or a doctor with an interest in health inequalities.

2) Over 18 years.

3) Able and willing to give informed consent at the time of the interview.

Inclusion criteria for the focus groups:

1) Nursing and allied health professional staff (e.g. physiotherapists, occupational therapists), professional, administrative and support staff (e.g. hospital teachers, play leaders, secretaries, receptionists, porters, security, cleaners).

2) Over 18 years.

3) Able and willing to give informed consent at the time of the interview.

Exclusion criteria

1) Age <18 years.

2) Does not have a current role as listed above in one of the ten children's hospitals.

4) Unable or unwilling to give informed consent.

Sampling technique

We will work across ten children's hospitals. We aim to recruit participants as described from each site, as per the funder's aim, but are aware that this may not be possible within the available time. Our aim is to work across all ten locations, with a view to accessing some participants from each category in a minimum of six locations. In each organisation, we will sample:

1. 1-3 members of the senior leadership team (one executive and one non-executive director plus any other relevant senior leader depending on the structure of the organisation). All participants will be over 18 years old and recruited based on their professional role. Because of their professional roles, all participants will be English speaking. It is not proposed to exclude on any further criteria. We will recruit a minimum of six and a maximum of 30 people.

2. 1-3 doctors with an interest in reducing health inequalities. All participants will be over 18 years old and recruited based on their professional role. Because of their professional roles, all participants will be English speaking. It is not proposed to exclude on any further criteria. We will recruit a minimum of six and a maximum of 30 people.

For peer review only - http://bmjopen.bmj.com/site/about/guidelines.xhtml

3. 4-8 nurses and allied health professionals (e.g. physiotherapists, occupational therapists). These potential participants will be recruited via their organisations. All participants will be over 18 years old and recruited based on their professional role. Because of their professional roles, all participants will be English speaking. It is not proposed to exclude on any further criteria. We will recruit a minimum of 24 and a maximum of 80 people.

4. 4-8 members of professional, administrative and support staff (e.g. hospital teachers, play leaders, secretaries, receptionists, porters, security, cleaners). These potential participants will be recruited via their organisations. All participants will be over 18 years old and recruited based on their professional role. Because of their professional roles, all participants will be English speaking. It is not proposed to exclude on any further criteria. We will recruit a minimum of 24 and a maximum of 80 people.

Recruitment

Senior leadership team: senior leaders will be identified via our gatekeeper in the National Paediatric Accelerator, who will provide organisational email addresses to the PI. Potential participants will then be provided with a detailed participant information sheet about the study and a copy of the consent form via email, and then asked to participate by Prof Isba as PI, on behalf of the research team. It will be made clear to potential participants that participation is voluntary.

Doctors: Doctors will be identified via our gatekeeper in the National Paediatric Accelerator who will consult each organisation to identify relevant individuals and provide organisational email addresses to the research team. Potential participants will then be provided with a detailed participant information sheet about the study and a copy of the consent form via email, and then asked to participate by one of the research team. It will be made clear to potential participants that participation is voluntary.

Nurses, allied health professionals, professional, administrative and support staff: These staff will be recruited via their organisations. Each children's hospital will be provided with information about the study, and asked to circulate a call for volunteers to participate via their preferred channel (e.g. email lists, newsletters). Potential participants will be asked to email the research team. The research team will then provide a detailed participant information sheet about the study and a copy of the consent form to all interested participants.

No payment will be made to participants.

Consent

Formal written consent supported by a participant information sheet will be sought for the recorded interviews and focus groups. This will be received by the researcher conducting the interview or focus group. On initial agreement by the participant to conduct the interview or focus group they will be provided with a copy of the consent form and participant information sheet. Due to the logistics of the study there will be a period of time between initial agreement and the interview or focus group taking place. This will allow enough time to ensure the participant is entirely happy to be involved and has time to ask questions. Given the study populations, no problems with understanding the consent process are anticipated.

6 ETHICAL AND REGULATORY CONSIDERATIONS

Assessment and management of risk

The research team have all undertaken Good Clinical Practice training, and completed Disclosure and Barring Service checks. As experienced researchers in healthcare, they are experienced in managing safeguarding issues. Limits to confidentiality will be outlined in the participant information sheets,

For peer review only - http://bmjopen.bmj.com/site/about/guidelines.xhtml

stating that if the participant or someone else is at significant risk of harm, the researcher will break confidentiality and speak to a member of the National Paediatric Accelerator Programme. If possible, the researcher will inform the participant before they do this.

All participants will be given a numeric reference/ pseudonym. For the focus groups and interviews, we will anonymise the data (using pseudonyms) in RAP sheets and onwards. Any identifying details (names/ details of incidents) will be anonymised in the report/ any future publications.

Information about confidentiality and its limitations will be included in each Participant Information Sheet, and will be explained to each participant before starting the interview/ focus group. We will verbally remind participants not to share personally identifiable data in focus groups, and not to share focus group content outside the session.

The interviews and focus groups do not cover areas that are potentially sensitive, but the research team are experienced in conducting interviews and focus groups on sensitive topics. We do not anticipate risks to participating in the study. Although participants may find participating interesting, there are no direct benefits in taking part. A Lancaster University Health and Safety assessment has been completed to ensure safety of the research team and participants.

Research Ethics Committee (REC) and other Regulatory review & reports

This study does not require NHS REC approval; however it does require HRA approval via the IRAS system and ethical approval from the Lancaster Faculty of Health and Medicine Ethics Committee. This has been confirmed via the HRA queries helpline.

Before the start of the study, a favourable opinion will be sought from the Lancaster FHM REC for the study protocol, informed consent forms and other relevant documents e.g. advertisements.

Substantial amendments that require review by the REC will not be implemented until that review is in place and other mechanisms are in place to implement at site.

All correspondence with the REC will be retained.

If the study is ended prematurely, the Chief Investigator will notify the REC, including the reasons for the premature termination.

Amendments

Amendments will be managed via the FHM REC application for amendment to previously approved research form. Following approval each site will be informed of the amendment via the research and development manager in each trust.

Amendments will be discussed with the funder and in quarterly oversight meetings.

Amendments will be tracked by keeping an electronic trail of applications and responses to applications for amendments in an access controlled OneDrive folder.

The decision to make an amendment will be taken by the CI with input from the research team.

Protocol compliance

Protocol deviations will be reported in writing to the sponsor (via the Faculty of Health and Medicine REC). Protocol violation will lead to an internal review of protocol with the research team. Recurring protocol deviations will pause the study and lead to substantive changes to the protocol and re-review by the Faculty of Health and Medicine REC.

For peer review only - http://bmjopen.bmj.com/site/about/guidelines.xhtml

End of study

The end of the study is defined as the submission of the final report to the funder.

Data protection and participant confidentiality

All investigators and study site staff will comply with the requirements of the General Data Protection Regulations and the Data Protection Act 2018. All use of personal information during the course of the project will be in accordance with the General Data Protection Regulations and the Data Protection Act 2018. We do not expect to collect any sensitive data.

Personal data (names, job roles, email addresses and phone numbers) will be given by participants when they contact the research team to arrange interviews/ focus groups. This information will only be used to arrange interviews and focus groups, and will be stored in a password-protected spreadsheet in an access-controlled OneDrive folder for the duration of the project. This information will be held separately from the data set. Personal data will only be accessible to the research team. Consent forms will be stored electronically in an access-controlled OneDrive folder, until the end of the project. If consent is taken in paper form, these forms will be scanned and uploaded to the access controlled OneDrive folder, as soon as possible after consent is taken, and originals will be securely shredded.

Lancaster University will be the data controller. For further information about how Lancaster University processes personal data for research purposes, please visit: www.lancaster.ac.uk/research/data-protection

Three sources of data will be gathered and analysed:

1. Interview video recordings.

Because interviews will be conducted via Microsoft Teams, these interviews will be video recorded. It is not possible to only audio record in Teams, so we will need to store video, though these will not be used for analysis. Video recordings will be accessed by another member of the research team, who will listen to the interview and complete a RAP sheet (see 3 below), which will then be stored digitally. Video recordings made on Teams are automatically stored on Lancaster University's Microsoft Stream platform. Only the meeting owner (the researcher/ project administrator) has access to the recording, but will grant access to another member of the research team for analysis (see above). All video recordings will be deleted by the meeting owner within six months of the end of the project (June 2023). From June 2022, automatic storage infrastructure will change, and videos will automatically be stored in OneDrive. The same access restrictions and plans for deletion will apply.

2. Focus group audio recordings. If pandemic protocols mean that focus groups have to be conducted remotely, video recording storage/ collection will apply (see 1 above).

Focus group audio recordings will be copied to the access controlled OneDrive folder and deleted from the capture device on the day of creation or as soon as possible afterwards. This prevents accidental data disclosure and guarantees that the data are backed-up. Digital recordings will be identified with a participant number only. Audio recordings of focus groups will be destroyed within six months of the end of the project. Audio and video recordings will not be transcribed in full, but anonymised quotations identified with a participant number may be transcribed into RAP sheets.

3. RAP sheets, completed by the research team.

RAP (Rapid Appraisal) sheets will be completed electronically in Microsoft Word and stored on the access-controlled OneDrive folder. They will constitute the main source of data from human participants to be retained and will be stored for ten years as per university policy. RAP and RREAL methodologies emerge from a qualitative research tradition that recognises the need for rapid analysis of data to produce meaningful recommendations for practice in a timely manner (Vindrola-Padros et al, 2020). RAP sheets will be used for team debriefs and analysis.

For peer review only - http://bmjopen.bmj.com/site/about/guidelines.xhtml

The researchers may take written notes as an aide-memoire of these focus groups, and of their general impressions and reflections on any informal encounters while onsite, but these notes will not constitute a formal source of data or contain identifiable data. These notes will be destroyed (shredded) following the completion of RAP sheets for the session.

Participants will be informed that they may withdraw from the study at any time before or during the interview/ focus group, and up to two weeks following their interview/ focus group participation without giving any reason. If the request to withdraw comes after data have been anonymised, and incorporated into themes, it might not be possible for it to be withdrawn, though every attempt will be made to extract the data, up to the point of delivering the report to the funder.

Lancaster University will act as the data custodian. Day-to-day data management will be undertaken by the PI. Written/ digital data will be stored in a Onedrive folder with access limited to the project team for ten years after the end of the project. After this, it will be deleted and responsibility for data management will lie with Professor Rachel Isba as principal investigator.

Indemnity

Lancaster University legal liability cover will apply:

- to meet the potential legal liability of the sponsor(s) for harm to participants arising from the management of the research
- to meet the potential legal liability of the sponsor(s) or employer(s) for harm to participants arising from the design of the research
- to meet the potential legal liability of investigators/collaborators arising from harm to participants in the conduct of the research

No arrangement for payment of compensation in the event of harm to the research participants where no legal liability arises has been made.

Personal research devises (laptop, mobile telephone) are insured by the researcher personally.

7 DISSEMINATION POLICY

As a mandatory clause in the consultancy contract, ownership of all intellectual property and data rights for the project will rest with Sheffield Children's Hospital (SCH) (on behalf of all the Trusts participating in the project) and we will not publish any aspect of the research without permission from Sheffield Children's Hospital. SCH agree that permission will not be unreasonably withheld, but data not be disseminated or shared for reuse.

We will produce a report and presentation of findings for the funder. We will discuss possible wider dissemination of selected elements of work via publishing in an academic journal or presenting at a conference with the funder after delivering this report.

8 REFERENCES

Arksey, H., & O'Malley, L. (2005). Scoping studies: towards a methodological framework. International Journal of Social Research Methodology, 8(1), 19–32. https://doi.org/10.1080/1364557032000119616

For peer review only - http://bmjopen.bmj.com/site/about/guidelines.xhtml

Daudt, H. M., van Mossel, C., & Scott, S. J. (2013). Enhancing the scoping study methodology: a large, inter-professional team's experience with Arksey and O'Malley's framework. BMC Medical Research Methodology, 13(1), 48. https://doi.org/10.1186/1471-2288-13-48

Edge Health (2021) "Analysis of inequalities and "was not broughts" for outpatients appointments". (Analysis prepared for the Paediatric Accelerator inequalities group). Unpublished

Khalil, H., Peters, M., Godfrey, C. M., McInerney, P., Soares, C. B., & Parker, D. (2016). An Evidence-Based Approach to Scoping Reviews. Worldviews on Evidence-Based Nursing, 13(2), 118–123. https://doi.org/10.1111/wvn.12144

Kvale, S., & Brinkmann, S. (2014). InterViews. SAGE Publications.

Marmot, M., Allen, J., Goldblatt, P., Herd, E., Morrison, J. (2020). Build Back Fairer: The COVID-19 Marmot Review. The Pandemic, Socioeconomic and Health Inequalities in England. London: Institute of Health Equity

Morse, J. M. (2015). Critical Analysis of Strategies for Determining Rigor in Qualitative Inquiry. Qualitative Health Research, 25(9), 1212–1222. https://doi.org/10.1177/1049732315588501

Peters, M. D., Godfrey, C., McInerney, P., Soares, C. B., Khalil, H., & Parker, D. (2017). Chapter 11: Scoping Reviews. In M. Z. Aromataris E (Ed.), Joanna Briggs Institute Reviewer's Manual. The Joanna Briggs Institute. Retrieved from https://reviewersmanual.joannabriggs.org/

Silverman, D. (2015). Interpreting Qualitative Data. SAGE.

Vindrola-Padros, C., Chisnall, G., Cooper, S., Dowrick, A., Djellouli, N., Symmons, S.M., Martin, S., Singleton, G., Vanderslott, S., Vera, N. and Johnson, G.A., (2020). Carrying out rapid qualitative research during a pandemic: emerging lessons from COVID-19. Qualitative health research, 30(14), pp.2192-2204

Vindrola-Padros, C., (2021) Doing Rapid Qualitative Research. SAGE.

For peer review only

For peer review only - http://bmjopen.bmj.com/site/about/guidelines.xhtml

9. APPENDICES

Appendix 1- Required documentation

- CV R Isba
- CV J Lunn
- CV L Brewster
- CV L Brennan
- Participant Information Sheet interviews
- Participant Information Sheet focus groups
- Consent Form interviews
- Consent Form focus groups
- Email interview invitation
- Focus groups promotional material
- Interview Guide
- Focus group guide
- RAP sheet

Appendix 2 – Schedule of Procedures

- HRA Schedule of Events
- HRA Organisation Information Document
- HRA Is My Study Research Result
- HRA Does My Study Need NHS REC Approval Result

Appendix 3 – Amendment History

No amendments – first submission

Amendment No.	Protocol version no.	Date issued	Author(s) of changes	Details of changes made

For peer review only

For peer review only - http://bmjopen.bmj.com/site/about/guidelines.xhtml

Children's Hospitals Inequalities Research Project

Lancaster University

Interview Schedule

Senior leaders, NEDs and doctors

Welcome and introduction - Seek consent to continue, reminder of audio-recording of interview (/video recording if on Teams), re-cap of project and plan for interview.

The interviews will explore your views on the Trust's policies and practices around health inequalities. We are interested in your perceptions, on the basis of your knowledge and experience. If you do not feel you are able to comment on any area please say so. Do you have any questions before we start?

1. What does the term 'the socio-economic inequalities in paediatric care' mean to you?
   - Prompt: an example of a socio-economic inequality in health is that children from the most deprived or poorest areas have a shorter life expectancy than people in the wealthiest areas.
2. In your experience, does deprivation have an impact on children's health?
   - Prompt: by deprivation we mean things like people living in areas with lower levels of income, employment, higher crime, less education
3. In your experience, does deprivation have an impact on whether or not children access care?
   - Prompt: What stops people from deprived areas from bringing their children to hospital for appointments and care?
4. Do you see health inequalities in the patients using this hospital?
   - What are these? How do they affect care (if not outlined above)
5. What do you see as your role in regard to health inequalities?
6. <u>Doctors only:</u> Are there any processes in place to help you, if you see health inequalities?
   - Prompt: have you received training, are there systems for flagging concerns?
   - Are there any barriers to this? Prompt: E.g. costs of care for patients, timings/ locations of appointments.
7. Does the Trust have a shared organisational view on its role in health inequalities?
   - Prompt: Are you aware of a statement by the board?
8. Are there any organisational policies in place relating to socio-economic barriers to accessing healthcare?
9. Do you think that COVID has affected socioeconomic inequalities?
   - (if yes) What is the Trust doing to address the impacts of COVID?
   - What impact, if any, has COVID had on access and quality at the Trust?

For peer review only - http://bmjopen.bmj.com/site/about/guidelines.xhtml

10. What does the Trust do to reduce health inequalities?
    - What could they do better/ more of?
    - Are there any barriers to this?

11. What would ‘best practice’ in addressing the socio-economic barriers to paediatric care look like?
    - Prompt: Have you seen evidence from elsewhere in the UK or internationally of what works well?

12. Senior leaders only: As an employer, does the Trust have a role in considering in wider society and socio-economic inequalities?

13. Senior leaders only: Any specific questions about hospital policies that have been reviewed for clarification.

Anything not covered?

Is there anything that we haven’t covered in the interview that you think we should know or think about?

Closing and thanks - Thank for their time and contribution.

For peer review only

Children's Hospitals Inequalities Research Project

Lancaster University

Focus Group Schedule

Nurses, Allied Health Professionals

Professional, administrative and support staff

Welcome and introduction - Seek consent to continue, reminder of audio-recording of the group, establish ground rules, re-cap of project and plan for focus group

The focus group will explore your views on the Trust's policies and practices around health inequalities. We are interested in your perceptions, on the basis of your knowledge and experience. Please try not to use any details which might lead someone in the group to be able to identify another staff member/ patient (e.g. their name). If you do not feel you are able to comment on any area please say so. Does anyone have any questions before we start?

1. In your experience, does deprivation have an impact on children's health?
   - Prompt: by deprivation we mean things like people living in areas with lower levels of income, employment, higher crime, less education
2. In your experience, does deprivation have an impact on whether or not children access care?
   - Prompt: What stops people from deprived areas from bringing their children to hospital for appointments and care?
3. Do you see health inequalities in the patients using this hospital?
   - What are these? How do they affect care (if not outlined above)
4. What do you see as your role in regard to health inequalities?
5. Do you know if the Trust has a policy, or a view, on its role in health inequalities?
   - (If yes) What is it? Can you say more?
   - (If no) Why do you think it does not have a policy/ view?
6. Are there any processes in place to help you, if you see health inequalities?
   - Prompt: have you received training, are there systems for flagging concerns?
   - Are there any barriers to this? Prompt: E.g. costs of care for patients, timings/ locations of appointments.
7. What does the Trust do to reduce health inequalities?
   - What could they do better/ more of?

Anything not covered?

Is there anything that we haven't covered in the focus group that you think we should know or think about?

Closing and thanks

Thank for their time and contribution.

For peer review only - http://bmjopen.bmj.com/site/about/guidelines.xhtml

Children's Hospitals Inequalities Research Project

Lancaster University

Study: Children's Hospitals Inequalities Research Project (CHIRP).

Researcher name	
Study location (name of hospital)	
Interview/ focus group (delete as applicable)	If focus group: number of participants =
Participant group (leadership, clinician, other)	
Examples of socio-economic inequalities	
Deprivation: impact on children's health	
Deprivation: impact on access to care	
Deprivation: visibility in their context (hospital)	
Perceived/ outlined role in health inequalities	
Awareness of policies/ policies and organisational views	
Processes in place for support	

For peer review only

Impact of Covid	
What the Trust currently does	
What the Trust might do more of/ better	
What stops the Trust doing more/ better	
Examples of best practice	
Clarification points (policies; SL only)	
Miscellaneous	
Other comments	

For peer review only

Standards for Reporting Qualitative Research (SRQR)*

http://www.equator-network.org/reporting-guidelines/srqr/

Title and abstract

	Page/line no(s).
**Title** - Concise description of the nature and topic of the study Identifying the study as qualitative or indicating the approach (e.g., ethnography, grounded theory) or data collection methods (e.g., interview, focus group) is recommended	1/ 1-3
**Abstract** - Summary of key elements of the study using the abstract format of the intended publication; typically includes background, purpose, methods, results, and conclusions	1/ 5-28

Introduction

**Problem formulation** - Description and significance of the problem/phenomenon studied; review of relevant theory and empirical work; problem statement	2/ 41-84
**Purpose or research question** - Purpose of the study and specific objectives or questions	3/ 85-86

Methods

**Qualitative approach and research paradigm** - Qualitative approach (e.g., ethnography, grounded theory, case study, phenomenology, narrative research) and guiding theory if appropriate; identifying the research paradigm (e.g., postpositivist, constructivist/ interpretivist) is also recommended; rationale**	3/89-100
**Researcher characteristics and reflexivity** - Researchers' characteristics that may influence the research, including personal attributes, qualifications/experience, relationship with participants, assumptions, and/or presuppositions; potential or actual interaction between researchers' characteristics and the research questions, approach, methods, results, and/or transferability	4/ 122-124
**Context** - Setting/site and salient contextual factors; rationale**	3-4/102-120
**Sampling strategy** - How and why research participants, documents, or events were selected; criteria for deciding when no further sampling was necessary (e.g., sampling saturation); rationale**	3-4/102-120 and 4154-158
**Ethical issues pertaining to human subjects** - Documentation of approval by an appropriate ethics review board and participant consent, or explanation for lack thereof; other confidentiality and data security issues	4/143-147
**Data collection methods** - Types of data collected; details of data collection procedures including (as appropriate) start and stop dates of data collection and analysis, iterative process, triangulation of sources/methods, and modification of procedures in response to evolving study findings; rationale**	4/121 - 142

For peer review only - http://bmjopen.bmj.com/site/about/guidelines.xhtml

**Data collection instruments and technologies** - Description of instruments (e.g., interview guides, questionnaires) and devices (e.g., audio recorders) used for data collection; if/how the instrument(s) changed over the course of the study	4/126 and 4/ 136 plus supplementary materials
**Units of study** - Number and relevant characteristics of participants, documents, or events included in the study; level of participation (could be reported in results)	3/108-112
**Data processing** - Methods for processing data prior to and during analysis, including transcription, data entry, data management and security, verification of data integrity, data coding, and anonymization/de-identification of excerpts	4/159-171
**Data analysis** - Process by which inferences, themes, etc., were identified and developed, including the researchers involved in data analysis; usually references a specific paradigm or approach; rationale**	4-5/149-177
**Techniques to enhance trustworthiness** - Techniques to enhance trustworthiness and credibility of data analysis (e.g., member checking, audit trail, triangulation); rationale**	5/172-177

Results/findings

**Synthesis and interpretation** - Main findings (e.g., interpretations, inferences, and themes); might include development of a theory or model, or integration with prior research or theory	5/178-346
**Links to empirical data** - Evidence (e.g., quotes, field notes, text excerpts, photographs) to substantiate analytic findings	5/178-346

Discussion

**Integration with prior work, implications, transferability, and contribution(s) to the field -** Short summary of main findings; explanation of how findings and conclusions connect to, support, elaborate on, or challenge conclusions of earlier scholarship; discussion of scope of application/generalizability; identification of unique contribution(s) to scholarship in a discipline or field	6/347-357 and 6/ 372-385
**Limitations** - Trustworthiness and limitations of findings	6/65-371

Other

**Conflicts of interest** - Potential sources of influence or perceived influence on study conduct and conclusions; how these were managed	Title page
**Funding** - Sources of funding and other support; role of funders in data collection, interpretation, and reporting	Title page

*The authors created the SRQR by searching the literature to identify guidelines, reporting standards, and critical appraisal criteria for qualitative research; reviewing the reference lists of retrieved sources; and contacting experts to gain feedback. The SRQR aims to improve the transparency of all aspects of qualitative research by providing clear standards for reporting qualitative research.

For peer review only - http://bmjopen.bmj.com/site/about/guidelines.xhtml

For peer review only

**The rationale should briefly discuss the justification for choosing that theory, approach, method, or technique rather than other options available, the assumptions and limitations implicit in those choices, and how those choices influence study conclusions and transferability. As appropriate, the rationale for several items might be discussed together.

Reference:

O'Brien BC, Harris IB, Beckman TJ, Reed DA, Cook DA. **Standards for reporting qualitative research: a synthesis of recommendations.** *Academic Medicine*, Vol. 89, No. 9 / Sept 2014 DOI: 10.1097/ACM.0000000000000388

For peer review only

For peer review only - http://bmjopen.bmj.com/site/about/guidelines.xhtml
